# Variable temperature *in situ* TEM mapping of the thermodynamically stable element distribution in bimetallic Pt–Rh nanoparticles†

**DOI:** 10.1039/d3na00448a

**Published:** 2023-08-24

**Authors:** Martin Jensen, Walace Kierulf-Vieira, Patricia J. Kooyman, Anja O. Sjåstad

**Affiliations:** a Center for Materials Science and Nanotechnology, Department of Chemistry, University of Oslo P.O. Box 1033 Blindern N-0315 Oslo Norway a.o.sjastad@kjemi.uio.no martin.jensen@kjemi.uio.no; b Catalysis Institute, Department of Chemical Engineering, University of Cape Town Private Bag X3 Rondebosch 7701 South Africa

## Abstract

We report here the first variable temperature *in situ* transmission electron microscopy (TEM) study on smaller Pt–Rh nanoparticles (≤24 nm) under vacuum conditions. Well-defined 50 at% Pt/50 at% Rh Pt–Rh solid solution and Rh(core)–Pt(shell) nanoparticles, obtained *via* colloidal synthesis routes, were investigated between room temperature and 650 °C to elucidate the tendency of elemental mixing/segregation. Key findings are that Pt–Rh nanoparticles <13 nm are stable in a solid solution configuration over the entire studied temperature range, whereas nanoparticles >13 nm tend to segregate upon cooling. Such a cross-over in element distribution with nanoparticle size has not been reported for the Pt–Rh system previously. The results demonstrate the technique's ability to extract valuable information concerning the intricate dynamic processes that take place in the bimetallic Pt–Rh system at the nanoscale, which may be indispensable when optimizing, *e.g.*, the metal composition in catalytically active materials.

## Abstract 2

Variable temperature *in situ* transmission electron microscopy (TEM) study to unravel the thermodynamically stable element distribution in Pt–Rh nanoparticles under vacuum.

## Graphical abstract

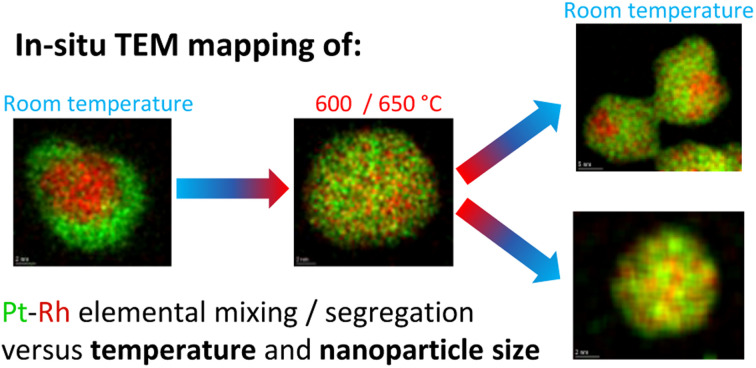

## Introduction

In the industrial scale production of synthetic nitrogen-based fertilizers, bimetallic Pt–Rh alloys in the form of wires, knitted or woven into gauzes, are widely used as catalysts for the ammonia (NH_3_) oxidation step.^1^ This makes bimetallic Pt–Rh nanoparticles candidate catalysts for efficient abatement of NH_3_ slip from the maritime sector by selective oxidation to nitrogen (N_2_). Such catalysis is already utilized in a two-step process for emission abatement in diesel engines, and with the first engines suited for NH_3_ combustion to be expected in 2025 (ref. 2 and 3) the establishment of even more robust NH_3_ abatement catalysts is becoming a pressing need. Notably, product selectivity toward N_2_, NO, and N_2_O depends on the Pt–Rh alloying in the surface atomic layers of the catalyst, reaction temperature, gas composition, and pressure.^4^ Thus, full control of the Pt–Rh alloying as well as knowledge of how the element distribution is dictated by process temperature and gas atmosphere are prerequisites for the design of optimized nanostructured Pt–Rh nanoparticles for selective NH_3_ oxidation.

In recent years, the collective competence in the field of colloidal nanoparticle synthesis has developed to yield well-defined bi- or multielement monodisperse nanoparticles with controlled faceting and element distribution.^5,6^ The as-synthesized nanoparticles can be trapped in certain metastable element distribution configurations, hindered by kinetic limitations to reach the thermodynamically preferred state.^7^ Consequently, the nanoparticles may reconstruct and even undergo chemical reactions (*e.g.*, oxidation) when exposed to the intended operational temperature-pressure conditions.^8^ Fundamental insight into the chemical state and elemental nano-structuring is therefore indispensable in understanding the catalytic reaction mechanisms and optimizing catalysts for applications. An excellent aid to unravel the elemental configuration and chemical state of such particles is *in situ* or operando TEM.

Based on theoretical modelling, Raub *et al.*^9^ predicted an immiscibility gap below 760 °C in the binary bulk Pt–Rh phase diagram. More recent reports^10–12^ (and references therein) conclude that instead of a miscibility gap, ordered low temperature (<−23 °C) structures occur.^10^ Notably, corresponding binary phase diagrams are not readily available for the nanoscale due to the complexity of the thermodynamic driving forces in play, *e.g.*, relative surface energies of the elemental constituents in question, presence of interfaces, nanoparticle size effects, faceting, and stabilizing ligands. Different surface energies of the two metals in a bimetallic nanoparticle can cause the metal with the lowest surface energy to migrate to the surface.^13^ Likewise, strain effects due to mismatch in atomic size may lead to segregation of the metal with the smallest atomic size to occupy parts of the nanoparticle under compressive strain.^13^ Furthermore, these effects can compete, as exemplified in the Ni–Rh system where Rh has a 20% higher surface energy than Ni but also an 8% larger atomic radius.^14^ The effect of strain energy on surface segregation is temperature dependent. For the bimetallic Ni–Rh, Au–Pt, Au–Pd, Cu–Pt and Ag–Pd systems the effect weakens with increasing temperature, see the ref. 14 and references therein. The particle faceting is also strongly correlated to the strain and affects the surface-to-volume ratio.^13^ Faceting is thus an important parameter. The various driving forces scale differently with particle size, implying the nanoparticle size itself is a parameter that affects the thermodynamic equilibrium. A Monte Carlo simulation based study of the size-dependence of low-temperature order–disorder transitions in the Pt–Rh system (7.8 nm, 4.3 nm, and 3.1 nm) has been reported by Pohl *et al.*^15^ Compared to the bulk, the disorder-order transition temperature was found to decrease as the nanoparticle size decreased.^15^ The literature also addresses several examples of nanoparticle or grain-size-dependent thermodynamic shifts for some bimetallic systems. For example, for the binary Ag–Sn system, a solid solution is stabilized for grains smaller than a critical size of 8 nm,^16^ whereas for Ag–Ni and Ag–Bi the corresponding critical nanoparticle/grain sizes for stabilizing a solid solution are reported to be 7 and 6 nm, respectively.^17,18^ Notably, for the binary Ag–Ni and Ag–Bi systems, computational simulations support the experimental findings.

A thorough literature review has not yielded any publication focused on the understanding of alloying properties of Pt–Rh nanoparticles in vacuum at variable temperatures by means of transmission electron microscopy (TEM). Inspired by the need to evaluate the suitability of bimetallic Pt–Rh nanoparticles as selective catalysts for NH_3_ slip abatement, we report here the first variable temperature *in situ* TEM study on the Pt–Rh nanoparticle system at vacuum conditions. Firstly, well-defined ∼50–50 at% Pt–Rh solid solution and Rh(core)–Pt(shell) nanoparticles were synthesized by means of colloidal routes using PVP as surface stabilizing agent. The *in situ* TEM experiments were carried out between room temperature and 650 °C to elucidate the tendency of elemental mixing/segregation of well-defined Pt–Rh solid solution and Rh(core)–Pt(shell) nanoparticles with respect to nanoparticle size and temperature. The scope of this work is limited to the investigation of nanoparticles (≤24 nm) unaffected by agglomeration and sintering effects.

## Experimental

Ethylene glycol (EG, ≥99%), 1,4-butanediol (99%), polyvinylpyrrolidone (PVP, average molar mass 10 000 g mol^−1^), [Pt(acac)_2_] (97%), and [Rh(acac)_3_] (97%) were obtained from Sigma-Aldrich. Acetone (100.0%) and methanol (100.0%) were purchased from VWR. All chemicals were used as received.

### Synthesis of Pt–Rh nanoparticles

In the current work, we followed the sample preparation procedures as first published in ref. 6. They are summarized here.

### Rh(core)–Pt(shell) nanoparticle synthesis

The Rh(core)–Pt(shell) nanoparticles were synthesized *via* a two-step sequential reduction reaction to grow a Pt-shell on Rh seeds. The Rh seeds were produced by first mixing 0.50 mmol (monomer unit) of PVP in 20 mL 1,4-butanediol, followed by removal of water through heating at 150 °C for 15 min under Ar-flow. After cooling to 100 °C, 0.050 mmol Rh(acac)_3_ was added and dissolved. A condenser was connected before the temperature was increased to 220 °C by switching to a higher temperature pre-heated heating block. The system was kept inert under Ar-flow during the reaction. After 120 min reaction time the system was allowed to cool to room temperature.

To grow the Pt-shell onto the pre-formed Rh seeds, 0.10 mmol of Pt(acac)_2_ and 0.50 mmol (monomer unit) of PVP were transferred to the reaction mixture containing the pre-formed Rh-seeds before the temperature was increased to 100 °C again under Ar-flow. The solution was stirred for 10 min to dissolve the Pt-precursor and PVP before heating to 190 °C for 18 h, followed by cooling to room temperature.

### Pt–Rh solid solution nanoparticle synthesis

Single-phase solid-solution Pt–Rh nanoparticles were synthesized *via* co-reduction of the metal precursors. Firstly, a solution containing 2.0 mmol (monomer unit) of PVP in 20 mL EG was dried by heating at 150 °C for 15 min under Ar-flow. The solution was cooled to 100 °C before the addition of the metal precursors; 0.10 mmol Rh(acac)_3_ and 0.30 mmol Pt(acac)_2_, which is expected to give particles with 50–50 at% Pt–Rh.^6^ A condenser was connected before the temperature was increased to 195 °C by switching to a higher temperature pre-heated heating block. The system was kept inert (Ar) and quenched to room temperature after 15 min of reaction time.

All nanoparticle samples were isolated by the nanoparticle washing procedure described in ref. 6 and re-dispersed in methanol in preparation for electron microscopy experiments.

### *In situ* TEM characterization

Scanning Transmission Electron Microscopy (STEM) images using a High-Angle Annular Dark Field (HAADF) detector, and Energy Dispersive X-ray Spectroscopy (EDS) maps, were acquired on a FEI Titan G2 60–300 kV equipped with a CEOS DCOR probe-corrector and Super-X-EDS detectors. The Field Emission Gun (FEG) electron source was operated at an acceleration voltage of 300 kV. For the *in situ* electron microscopy characterization, a Protochips Fusion Select® system was used with a silicon nitride type Heating E-chip with nine windows. The temperature accuracy and uniformity of the system were >95% and 99.5%, respectively.^19^ The heating/cooling (quenching) rates were >140 °C s^−1^. All EDS analyses were performed using the Velox® software package (Version 3).

According to the literature, the electron beam can cause redistribution of elements within a nanoparticle sample, and the effect is exponentially correlated to temperature.^20^

To avoid effects from the electron beam on the element distribution dynamics during the *in situ* TEM experiments, we therefore took several precautions. As electron beam effects occur as a result of electron dose,^20^ we used a low electron beam current (screen current of 50 pA), made sure that none of the windows in the E-chip were exposed to the beam before the experiment started, and we kept track of which particles were exposed to the beam during the propagation of the experiment. In addition, HAADF-STEM images and corresponding EDS maps were only acquired on particles previously unexposed to the beam. Prior to data analysis, the sequence of frames obtained during the EDS-scan was scrutinized to determine when beam effects started to be detected. Consequently, only the first few frames in each of the EDS elemental map data sets, without detectable beam effects, were used - excluding beam effects from the data analysis. This implies that the sole external input to affect the observed dynamics in the elemental distribution within the individual nanoparticles is the temperature set for the experiment.

## Results

### Thermal stability of Pt–Rh nanoparticles

The scope of the current study is to explore the dynamics in element distribution of bimetallic Pt–Rh nanoparticles (≤24 nm), unaffected by agglomeration and sintering effects, at variable temperature in vacuum. Both core–shell and solid solution Pt–Rh nanoparticles were utilized, and the average composition of the core–shell batch 1, core–shell batch 2, and the solid solution nanoparticles was approximately 50 at% Pt and Rh (see ESI sections 1–3† for details). Three different experiments were carried out in the temperature range from 25 to 650 °C. However, to prevent distortion of data and conclusions due to electron beam effects, high-resolution HAADF-STEM imaging and EDS mapping were limited to a maximum temperature of 600 °C.

#### 1. Combined *in situ* TEM and TEM characterization of Rh(core)–Pt(shell) nanoparticles heated to 650 °C

**Fig. 1:**
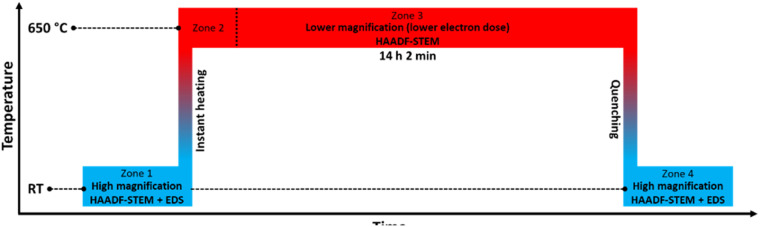
Temperature program used in the *in situ* TEM heating experiments of the Rh(core)–Pt(shell) nanoparticles (*D*_avg_ = 9.0 ± 2.5 nm) heated to 650 °C. The experiment is divided into 4 zones.

In this experiment, Rh(core)–Pt(shell) nanoparticles (batch 1) were instantly heated from room temperature to 650 °C and annealed for 14 h before quenching, see the temperature program and the four zones of data acquisition in Fig. 1.

**Fig. 2:**
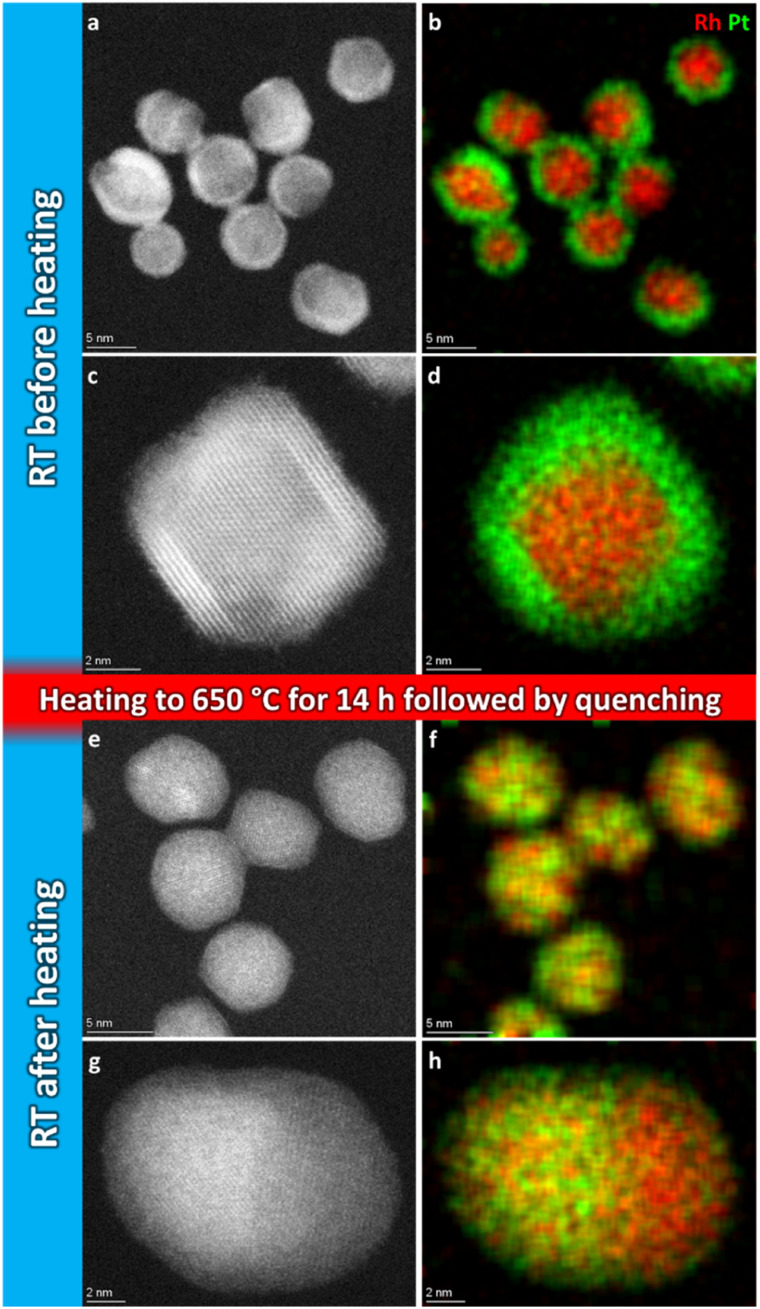
Representative selection of HAADF-STEM images and EDS elemental maps of Rh(core)–Pt(shell) nanoparticles before (a–d) and quenched after (e–h) heat treatment for 14 h 2 min at 650 °C.

Fig. 2a–d show representative HAADF-STEM images and corresponding EDS elemental maps of the Rh(core)–Pt(shell) nanoparticles prior to heating, zone 1 in Fig. 1 (see also ESI, section 1†). The sample is well-defined in terms of element distribution, and from the high-resolution HAADF image (Fig. 2c), a sharp separation between the two constituents, the Pt-rich shell and the Rh-rich core, is clear. This is in line with our previous findings.^6^

Initially, we attempted to carry out high-magnification *in situ* HAADF-STEM imaging and EDS mapping at 650 °C (zone 2, Fig. 1), but electron beam induced damage occurred too fast at this temperature (see Experimental section for explanation). Therefore, we report high magnification HAADF-STEM images and EDS elemental maps collected on nanoparticles unexposed to the electron beam after quenching from 650 °C (zone 4, Fig. 1); see Fig. 2e–h. From an analysis of 34 nanoparticles (average size *D*_avg_ = 9.0 ± 2.5 nm, zone 4), 29 particles formed a Pt–Rh solid solution, four were partly segregated, and one was fully segregated. Interestingly, the solid solution particles were significantly smaller with average size *D*_avg_ = 8.4 ± 1.7 nm (size range 6–13 nm) than the fully segregated one at 16 nm. The partially mixed particles had *D*_avg_ = 11.3 ± 4.0 nm, in the size range 7–17 nm. We take this as an indication that the preferred element distribution at 650 °C for smaller nanoparticles is solid solution.

**Fig. 3:**
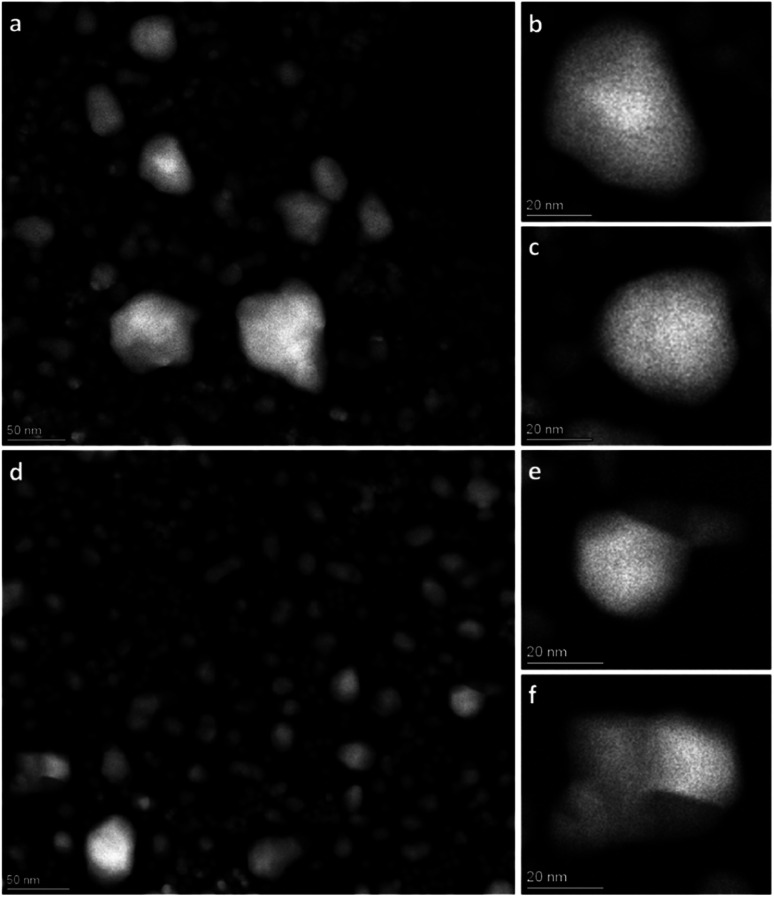
Overview HAADF-STEM images of Rh(core)–Pt(shell) nanoparticles at 650 °C after 1 h 19 min (a–c) and after 13 h 9 min (d–f).

In an endeavor to overcome the electron beam damage at 650 °C (zone 2, Fig. 1) and to collect *in situ* images, we continued the experiment by working at lower magnification and limiting the characterization to HAADF-STEM imaging only, to significantly lower the electron dose (zone 3, Fig. 1). The TEM results collected in zone 3 are reported in Fig. 3. From the results, qualitatively we see that after 1 h 19 min at 650 °C (Fig. 3a–c), the nanoparticle size distribution had drastically changed.

**Fig. 4:**
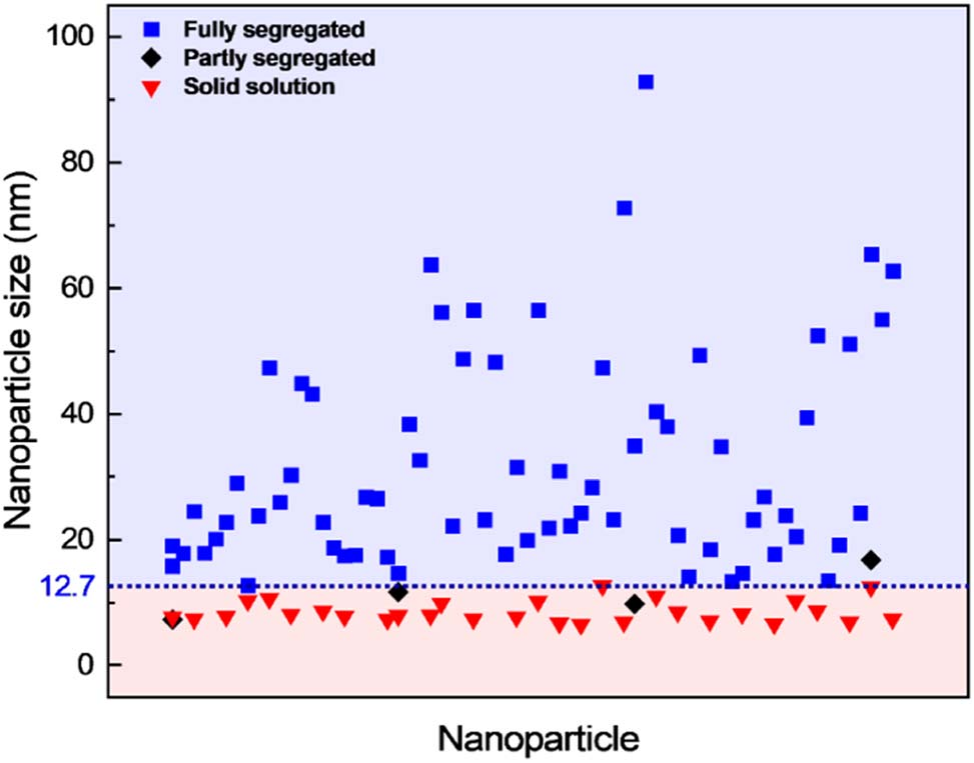
Element distribution *versus* nanoparticle size. Nanoparticle size of fully segregated (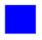) particles at 650 °C from lower magnification overview HAADF-STEM images (including the one in Fig. 3d). Only the fully segregated nanoparticles were measured from the lower magnification overview images (see justification in ESI section 1.2†). Nanoparticle size of solid solution (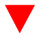), partly segregated (◆), and fully segregated (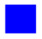) particles from high magnification, high-resolution HAADF-STEM images and complementary EDS maps at room temperature after quenching from 650 °C (Fig. S5,† ESI section 1.2†).

The contrast in the HAADF-STEM images, however, implies that Pt and Rh are mixed in a solid solution configuration for all particle sizes. This observation is in line with a redistribution of Pt and Rh into a solid solution configuration, accompanied by particle growth. Notably, after 13 h 9 min at 650 °C the situation has dramatically changed. We observe clear segregation by Z-contrast in some of the larger particles in the HAADF-STEM images in Fig. 3d–f. No such contrast is evident after 1 h 19 min. Correlating nanoparticle size to element distribution, apparently only nanoparticles above approximately 13 nm are fully segregated, see Fig. 4. This indicates there is a nanoparticle size induced reversal in elemental distribution in the Pt–Rh system, with a critical size of approximately 13 nm: *i.e.*, below ∼13 nm the nanoparticles are stabilized as a solid solution. To explain all findings in the TEM images reported in Fig. 3a–f, one must take into consideration that in parallel with the elemental reconfiguration process, agglomeration and particle growth by sintering also take place. We thus should expect to find some of the larger particles to still be in a mixed solid solution configuration, and some that are segregated; see Discussion section for a justification.

#### 2. *In situ* TEM characterization of Pt–Rh solid solution nanoparticles heated to 600 °C

**Fig. 5:**
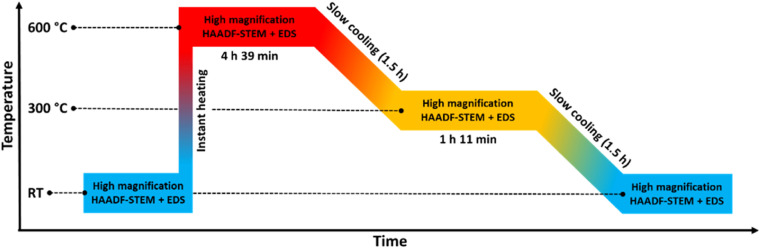
Temperature program used in the *in situ* TEM heating experiments of the Pt–Rh solid solution nanoparticles (*D*_avg_ = 8.9 ± 2.0 nm) heated to 600 °C and stepwise cooled to room temperature (RT).

To elaborate on the hypothesis that Pt–Rh nanoparticles ≲ 13 nm are stable in a solid solution configuration over the full temperature range (25–650 °C), well-defined solid solution Pt–Rh nanoparticles were heated to 600 °C and cooled down stepwise to room temperature following the temperature program sketched in Fig. 5. With focus on analysis of smaller, not agglomerated and sintered nanoparticles, and selecting a maximum temperature of 600 °C to avoid electron beam damage, EDS elemental mapping in addition to acquiring HAADF-STEM images at the various temperature steps were feasible.

**Fig. 6:**
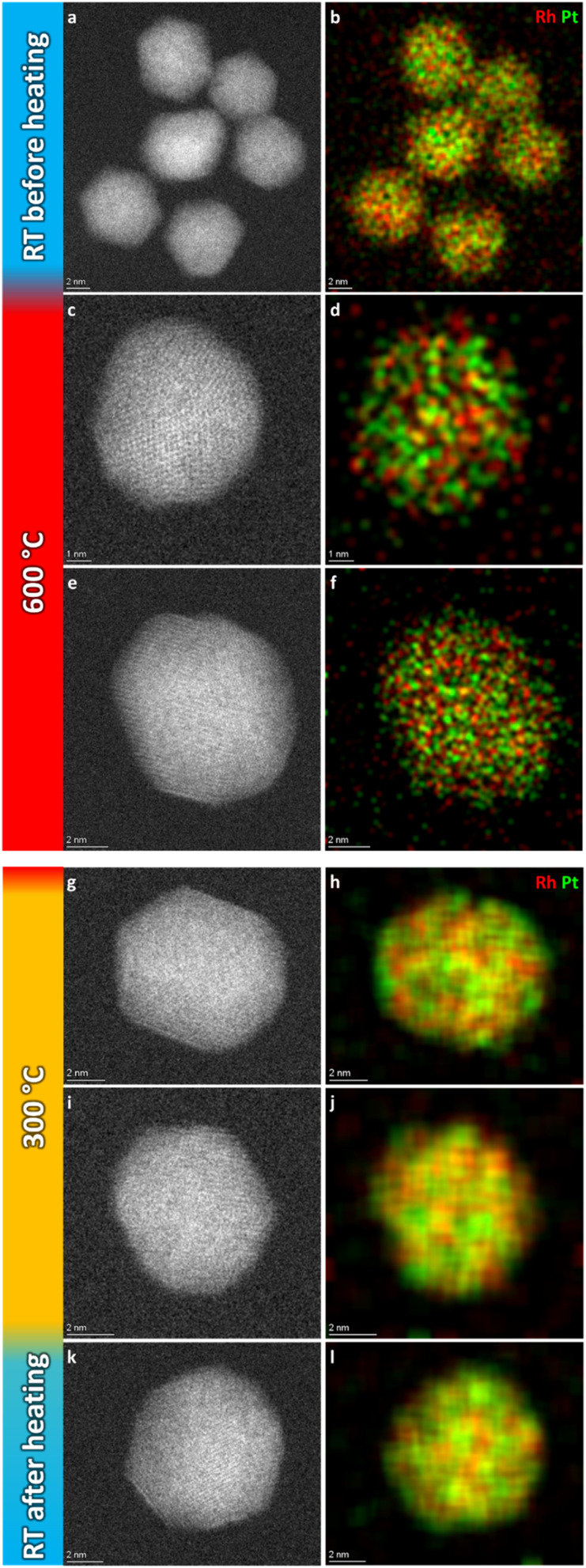
Representative selection of HAADF-STEM images and EDS elemental maps of Pt–Rh solid solution nanoparticles at the respective temperature steps.

Fig. 6a and b show a representative selection of the as-synthesized nanoparticles. Both HAADF-STEM and EDS maps show the particles to be single-phase solid solution, in line with previous findings.^6^ Representative nanoparticles were analyzed in the same way at 600 °C, 300 °C, and after cooling to room temperature (more details are given in ESI, section 2†). The results show that the element distribution does not change with temperature variations, indicating the nanoparticles are stable in a solid solution configuration over the entire temperature range.

The nanoparticles analyzed at 600 °C, 300 °C and at room temperature after cooling had *D*_avg_ = 8.3 ± 1.9 nm, *D*_avg_ = 9.0 ± 1.2 nm and *D*_avg_ = 9.3 ± 2.4 nm, with size ranges 6–12 nm, 8–11 nm and 5–15 nm, respectively. Thus, all the particles were either smaller than or close to the critical segregation size of ∼13 nm. The average size of all nanoparticles analyzed in this temperature range was *D*_avg_ = 8.9 ± 2.0 nm.

#### 3. *In situ* TEM characterization of larger Rh(core)–Pt(shell) nanoparticles heated to 600 °C

In view of the segregation occurring in some of the larger (≳13 nm) core–shell nanoparticles after heating to 650 °C (Fig. 4), we investigated if larger (*D*_avg_ = 15.0 ± 3.0 nm, range 9–24 nm) Rh(core)–Pt(shell) nanoparticles would segregate when exposed to the same heating program as employed for the as-synthesized solid solution nanoparticles described in Section 2.

**Fig. 7:**
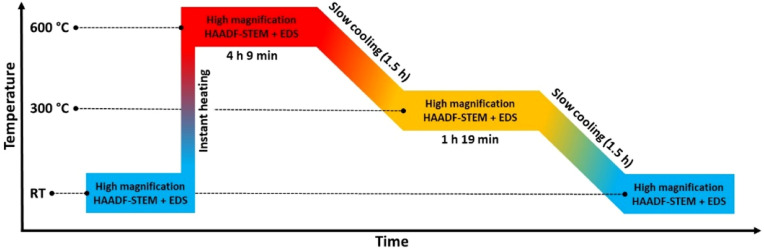
Temperature program used in the *in situ* TEM heating experiment of the larger (*D*_avg_ = 15.0 ± 3.0 nm) Rh(core)–Pt(shell) nanoparticles heated to 600 °C and step wise cooled back to room temperature (RT) in a controlled manner.

We therefore carried out an extra *in situ* TEM experiment where the slightly larger size fraction (batch 2) relative to batch 1 of Rh(core)–Pt(shell) nanoparticles was investigated by HAADF-STEM imaging and complementary EDS mapping following the heating program sketched in Fig. 7.

**Fig. 8:**
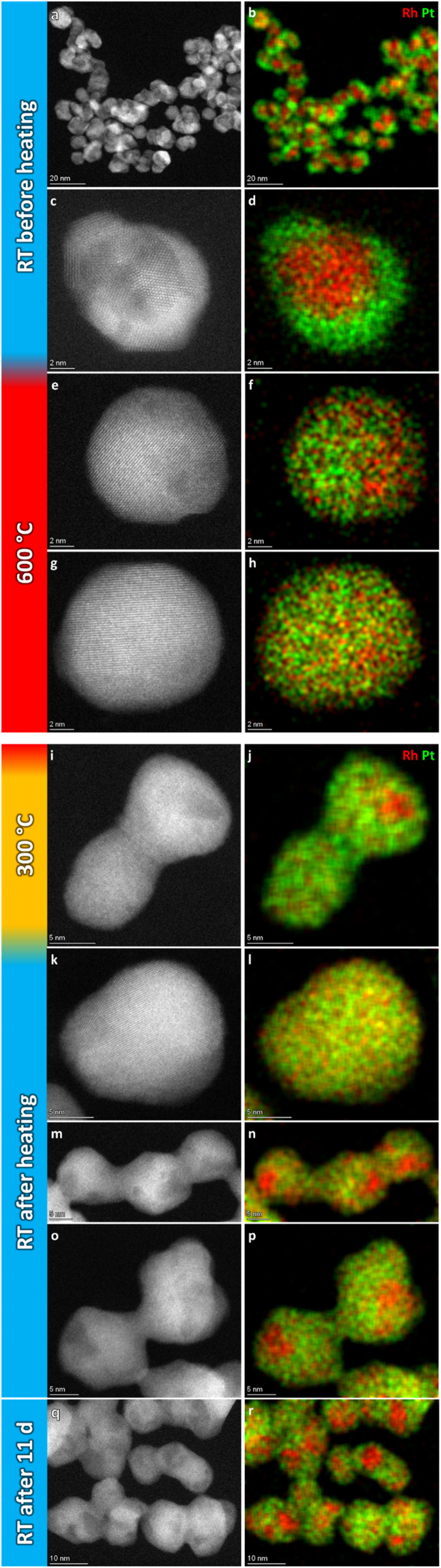
Representative selection of HAADF-STEM images and EDS elemental maps of Rh(core)–Pt(shell) nanoparticles at the respective temperature steps.

For the record, HAADF-STEM images and corresponding EDS elemental maps of the Rh(core)–Pt(shell) nanoparticles (batch 2) were shown to be well defined before heating (Fig. 8a–d and ESI, section 3.1†). The nanoparticles heated to 600 °C (Fig. 8e–h) are distinctively different from the images at room temperature in terms of element distribution. Overall, based on the analysis (ESI, section 3.2†), five nanoparticles are in a solid solution configuration with *D*_avg_ = 15.1 ± 3.0 nm (range 10–18 nm), seven are partly segregated with *D*_avg_ = 15.0 ± 2.6 nm (range 12–19 nm), while two are more segregated (11–13 nm). Notably, the two more clearly segregated nanoparticles are observed at the two earliest time points at 600 °C, thus might not have had time to fully transform. An explanation for the observed larger size range of the solid solution nanoparticles at 600 °C, which exceeds the previously identified critical size limit from the 650 °C experiment (Section 1), is provided in the Discussion section. Upon cooling to 300 °C, nine of the particles are solid solution with *D*_avg_ = 15.8 ± 3.3 nm (range 11–21 nm), one particle is partly segregated (17 nm) with five particles exhibiting more distinct segregation with *D*_avg_ = 13.7 ± 1.8 nm (range 12–16 nm); Fig. 8i–j and ESI, Section 3.3.† When returning to room temperature, only two of the 19 analyzed particles were in the solid solution configuration (19–24 nm) (Fig. 8k–p; ESI section 3.4†). The segregated particles with *D*_avg_ = 14.4 ± 2.9 nm (range 9–21 nm) show resemblance to the as-synthesized sample, with Rh-rich volumes encapsulated by a Pt-rich shell. Since the two fully mixed nanoparticles were imaged just after returning to ambient temperature, we revisited the sample after keeping it at room temperature in vacuum for 11 days; see Fig. 8q and r and ESI section 3.5.† All nanoparticles with *D*_avg_ = 14.9 ± 2.9 nm (range 11–22 nm) were now found to be segregated. The average size of all segregated nanoparticles in total after cooldown was *D*_avg_ = 14.7 ± 2.9 nm. It should be noted that the particles with diameter ≲ 13 nm were all residing in very close vicinity to other particles and had sintered together. This means it is unclear if they should be interpreted as a single entity with respect to element distribution, as mass transport is likely to occur throughout the particle assembly. Although the segregated particles at room temperature are similar to the as-synthesized sample, they differ in one aspect: The Rh-rich phase does not reside in the center of the particles, but is decentralized, which is evidence that the particles have undergone change with respect to the as-synthesized element spatial distribution, which is also observed in the Au–Ni system.^21^ The experimental results obtained can be subject to multiple interpretations, and a comprehensive discussion of these different perspectives is presented in the Discussion section.

## Discussion

**Fig. 9:**
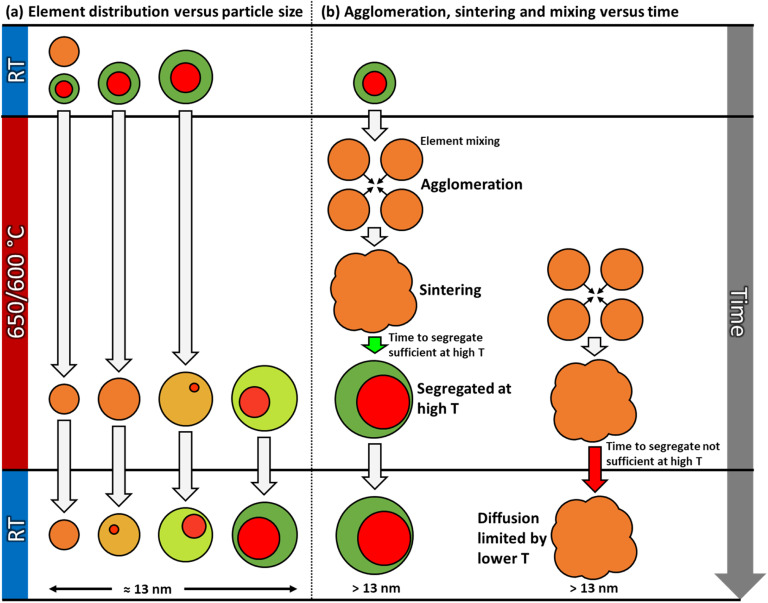
Schematic illustration of various dynamic processes occurring in the Pt–Rh nanoparticle samples during the *in situ* TEM heating experiments in vacuum. As seen in the element distribution *versus* size effect (a); throughout the entire temperature range, solid solution is favored below a certain size limit (suppression of miscibility dome). As the nanoparticle size is varied from small to larger above the approximate critical size of 13 nm, segregation is favored (left to right). Particles smaller than the critical size may first mix to form a solid solution at 650/600 °C, and then grow into a particle larger than this size by sintering. As shown in (b), large solid solution particles may segregate if given sufficient time (green arrow), or be kinetically trapped in this state for a long time due to diffusion and time limitations (red arrow) before segregation occurs, and thus both segregated and fully mixed particles coexist after long dwell times at 650 °C.

This work describes the evolution in elemental restructuring in well-defined as-synthesized Pt–Rh solid solution and Rh(core)–Pt(shell) PVP surface-stabilized nanoparticles upon heating using *in situ* TEM from room temperature to 650 °C in vacuum. The net composition of the particles is approximately 50 at% Pt and 50 at% Rh. Special care is taken to prevent electron beam effects, leading to three key findings (see also Fig. 9):

(i) As-synthesized Rh(core)–Pt(shell) (*D*_avg_ = 9.0 ± 2.5 nm), and Pt–Rh solid solution (*D*_avg_ = 8.9 ± 2.0 nm) nanoparticles exposed to 650 and 600–25 °C (Fig. 2 and 6), respectively, demonstrate the smaller nanoparticles (≲13 nm) to be stable in the form of a solid solution over the entire temperature interval (25–650 °C).

(ii) For the slightly larger as-synthesized Rh(core)–Pt(shell) nanoparticles, upon heating to 600 °C, the element distribution is either transformed to a situation with complete (*D*_avg_ = 15.1 ± 3.0 nm) or partial (*D*_avg_ = 15.0 ± 2.6 nm) mixing of Pt and Rh into a solid solution. Upon cooling to room temperature, the solid solution/partially mixed Pt–Rh nanoparticles segregate (*D*_avg_ = 14.7 ± 2.9 nm) to yield the Rh(core)–Pt(shell) configuration (Fig. 8).

(iii) Pt–Rh solid solution nanoparticles grown by sintering to a diameter ≳ 13 nm segregate when given sufficient time (13 h 9 min) at 650 °C to reach equilibrium (Fig. 3 and 4).

The observation of (i) complete miscibility of the smallest size fraction of Pt–Rh nanoparticles in the entire studied temperature interval, (ii) the evidence of complete or partial element mixing at 600 °C and Pt–Rh segregation in the slightly larger nanoparticles upon cooling to room temperature, and (iii) full segregation taking place in some of the largest particles (≳13 nm) exposed to 650 °C for a long dwell time, allows us to conclude that this specific Pt–Rh system provides a nanoparticle size-induced miscibility gap or a cross-over in the energetics of the phase diagram to favor phase separation at lower temperatures for larger nanoparticle sizes. The critical diameter is around 13 nm, however, as will be discussed, this diameter is not expected to be very well defined. Due to the method of determining the size of the nanoparticles by measuring their diameter across their largest cross-section, which assumes an ideal spherical shape, the critical diameter is expected to be imprecise. Furthermore, we do not consider any other characteristics, such as faceting, in the current argumentation on the nanoparticle stability. The uncertainty in the critical size for segregation is evident from our observations that the as-synthesized slightly larger Rh(core)–Pt(shell) nanoparticles were seen to transform to fully mixed (10–18 nm) and partly segregated (12–19 nm) configurations at 600 °C, despite the size ranges somewhat exceeding the lower size limit (∼13 nm) for full segregation observed in the experiment at 650 °C.

However, we also observed partly segregated particles in the size range 7–17 nm after a long dwell time at 650 °C. These findings do not overshadow the fact that practically all particles ≳13 nm segregated when cooled to room temperature from 600 °C, clearly suggesting the presence of an immiscibility dome which is shifted to higher temperature for this slightly larger nanoparticle size fraction, compared to the smaller ones (≲13 nm) (see Fig. 9a).

One can question if the observed difference in behavior between the smaller and larger Pt–Rh solid solution nanoparticles upon cooling is caused by insufficient atomic mobility or too short dwell time to equilibrate. We rule out that our conclusion is hampered by nanoparticles being given insufficient time for equilibration, for two reasons. Firstly, a large solid solution particle requires longer atomic transport paths relative to the smaller particles. Secondly, it has been established that diffusivity is enhanced in smaller nanoparticles.^22^ Hence, as within the same time scale larger solid solution nanoparticles are observed to segregate upon cooling whereas the smaller ones stay in solid solution (Fig. 9a), we conclude we have given the nanoparticles sufficient time to undergo the required elemental reconstruction. To the best of our knowledge, no such nanoparticle size dependent differences have been reported for the Pt–Rh system. Notably, our finding is in line with results from other bimetallic systems. In the Introduction section, we discussed literature reporting a critical lower size for obtaining a stable single phase solid solution configuration in the systems Ag–Sn,^16^ Ag–Bi,^18^ and Ag–Ni.^17^ However, detailed studies for evaluating the exact particle size – elemental distribution correlation at different compositions are suggested as future work. In this context, also the effect of external factors such as ligands (PVP) will be evaluated.

It is worth noting that not all the nanoparticles with diameter ≳ 13 nm were fully segregated after 13 h 9 min at 650 °C (Fig. 3d–f). This observation contrasts with our conclusion that solid solution nanoparticles with a diameter ≳ 13 nm segregate at this temperature. A plausible explanation for the coexistence of large segregated and solid solution nanoparticles is that when nanoparticles are annealed at elevated temperatures, several dynamic processes occur simultaneously, but at different time scales.

More concretely, in parallel with the thermodynamically driven nanoparticle size dependent elemental mixing/de-mixing processes, coalescence and sintering of the nanoparticles may take place. The change in nanoparticle size due to these processes may in itself induce cross-over in stable element distribution configuration within the particles, as well as the diffusion distances increase and the atomic mobility decreases^22^ (Fig. 9). The acquisition of *in situ* TEM data as a function of time (Fig. 3) enables the decoupling of the various processes. The starting time for the sintering of a particular particle ensemble dictates how far elemental reconfiguration has progressed when the TEM image and EDS map are acquired. Depending on the progress of the sintering process, we may find some larger assemblies, with atomic mobility sufficiently low and with long diffusion distances, that have not yet reached equilibrium (Fig. 9b). Therefore, a more solid solution situation was observed in these larger ensembles even though the nanoparticle size was larger than the apparent thermodynamical phase segregation critical size of ≳13 nm. On the other hand, for particles where the sintering process started much earlier, these particles have had time to age and thus equilibrate (phase separation). This explains why some of the large particles in Fig. 3d–f have reached the segregated equilibrium situation while others still have more solid solution elemental distribution. However, the potential role of interface effects or the relative grain size in the particles is not included in this reasoning. Importantly, the as-synthesized solid solution particles are free from interfaces, whereas both smaller and larger Rh(core)–Pt(shell) nanoparticles contain interfaces. As pointed out by Tiwari *et al.*,^23^ additional interfaces present in biphasic alloy nanoparticles, as compared to nanoparticles composed of a single-phase alloy, can significantly affect the phase transformation behavior. This effect is also particle size dependent.

## Concluding remarks and perspectives

In this work we have shown experimentally for the first time that the tendency for elemental mixing- or segregation to occur in Pt–Rh nanoparticles depends on the nanoparticle size and temperature. The finding is based on *in situ* TEM investigations of well-defined 50 at% Pt/50 at% Rh Pt–Rh solid solution and Rh(core)–Pt(shell) nanoparticles (≤24 nm) in vacuum. The nanoparticles were produced *via* colloidal synthesis routes using PVP as stabilizing agent, and were investigated in the temperature range between room temperature and 650 °C. We identified that smaller nanoparticles (≲13 nm) are stable in the solid solution configuration over the entire studied temperature range. Larger nanoparticles (≳13 nm) tended to segregate when cooled to room temperature.

The current study demonstrates *in situ* TEM to be a powerful technique capable of giving insight into and decouple the many dynamic processes that occur in bimetallic nanoparticle systems in the explored temperature range. The results obtained are of importance to understand the thermodynamics of this specific Pt–Rh nanoparticle system, but also to compare to other metallic nanoparticle systems. One natural next step is to elaborate experimentally by *in situ* TEM on the extent of a possible miscibility dome with respect to Pt–Rh composition and nanoparticle size, including the role of interfaces and grain structure. In a wider perspective, our findings will add value towards applications like catalysis, whereof *e.g.* supported Pt–Rh nanoparticles are attractive candidates for NH_3_ slip abatement processes. In this context, *in situ*/operando experiments using reactive gases as O_2_ and NH_3_ on element distribution will shed light on the stable element distribution at operative conditions. The nanoparticles used in this study are excellent model materials, even though they are stabilized with ligands. For more direct correlations concerning the thermodynamics of Pt–Rh alloys and for catalytic applications, gentle surfactant removal will be attempted.

Finally, it should be noted that the *in situ*/operando TEM technique at elevated temperatures requires awareness of system specific intrinsic properties such as metal evaporation at high vacuum or in inert gas atmosphere,^24^ the role of reactive gases on the formation of volatile metal containing species,^24^ size dependent stabilization of metal particles on various supports (strong metal support interaction)^25^ as well as various beam effects.^20^ Clear-cut findings extracted from the current TEM experiments rely fully on access to well-defined solid solution and core–shell Pt–Rh nanoparticles with equal net composition and nanoparticle size. Obtaining such nanoparticles requires high-level control of the synthesis, which is an art in itself.^6^

## Conflicts of interest

There are no conflicts to declare.

## Supplementary Material

NA-005-D3NA00448A-s001
