# Supplementary material for: Variable temperature in situ TEM mapping of the thermodynamically stable element distribution in bimetallic Pt–Rh nanoparticles
Source: Nanoscale Adv. 2023 Aug 24;5(19):5286–94. doi: 10.1039/d3na00448a (PMC10521204; doi:10.1039/d3na00448a)
Supplement: NA-005-D3NA00448A-s001 [file NA-005-D3NA00448A-s001.pdf]

Electronic Supplementary Material (ESI) for Nanoscale Advances.
This journal is © The Royal Society of Chemistry 2023

Table of Contents Supplementary Information

1. In-situ Transmission Electron Microscopy study of Rh(core)-Pt(shell) nanoparticles heated to 650 °C 1

1.1. Rh(core)-Pt(shell) nanoparticles at room temperature before heating to 650 °C 1

1.2. Rh(core)-Pt(shell) nanoparticles heated to 650 °C 7

Justification for particle selection in Fig. 4 (main manuscript) 17

2. In-situ Transmission Electron Microscopy study of Pt-Rh solid solution nanoparticles heated to 600 °C 18

2.1. Solid solution nanoparticles at room temperature before heating 18

2.2. Pt-Rh solid solution nanoparticles heated to 600 °C 27

2.3. Pt-Rh solid solution nanoparticles at 300 °C after cooling from 600 °C 36

2.4. Pt-Rh solid solution nanoparticles at room temperature after cooling from 300 °C 41

3. In-situ Transmission Electron Microscopy study of larger Rh(core)-Pt(shell) nanoparticles heated to 600 °C 49

3.1. Larger Rh(core)-Pt(shell) nanoparticles at room temperature before heating 49

3.2. Larger Rh(core)-Pt(shell) nanoparticles heated to 600 °C 52

3.3. Larger Rh(core)-Pt(shell) nanoparticles at 300 °C after cooling from 600 °C 60

3.4. Rh(core)-Pt(shell) nanoparticles at room temperature after cooling from 300 °C 65

3.5. Rh(core)-Pt(shell) nanoparticles after heating, then after 11 days at room temperature ... 72

References 76

1. In-situ Transmission Electron Microscopy study of Rh(core)-Pt(shell) nanoparticles heated to 650 °C

In this section, additional data supplementing the in-situ TEM results from section 1 in the main manuscript of the Rh(core)-Pt(shell) nanoparticles (batch 1) will be presented.

For convenience, the temperature program for the in-situ TEM experiment from the main manuscript (Fig. 1) is also shown here (Fig. S1). As mentioned in the main manuscript, we collected HAADF-STEM images at lower magnification at 650 °C (Fig. S1, zone 3) to avoid beam effects, because lower magnification lowers the electron dose on the sample. To extract information concerning the elemental distribution at the high temperature, we quenched the sample to room temperature aiming to freeze the elemental distribution state and enable high-resolution data collection without beam effects (Fig. S1, zone 4).

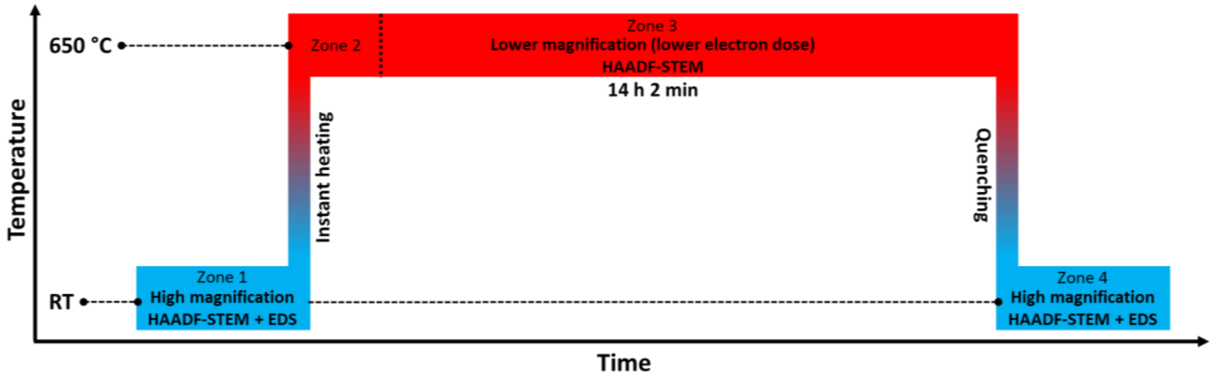

Fig. S1 Temperature program used in the in-situ TEM heating experiments of the Rh(core)-Pt(shell) nanoparticles heated to 650 °C. The experiment is divided into 4 zones.

1.1. Rh(core)-Pt(shell) nanoparticles at room temperature before heating to 650 °C

We first characterized the nanoparticles at room temperature before heating (Fig. S1, zone 1) to verify that the batch was well-defined in terms of elemental distribution (Fig. S2).

From the HAADF-STEM images and EDS elemental maps in Fig. S2 we conclude that the Rh(core)-Pt(shell) nanoparticles are well-defined in terms of elemental distribution and are well suited for the heating experiment to study the element distribution dynamics upon temperature variation.

The chemical composition of a selection (NP48 – 58) of individual Rh(core)-Pt(shell) nanoparticles was determined by quantitative EDS analysis. The areas selected for analysis are shown in Fig. S3 and the results are summarized in Table S1. The chemical composition was plotted versus nanoparticle size (Fig. S4), and no correlation between the two parameters was identified. For NP48 – 58, the average composition of the nanoparticles ($\pm 1\sigma$) normalized to Pt and Rh was 50.5 ± 6.2 at. % Pt.

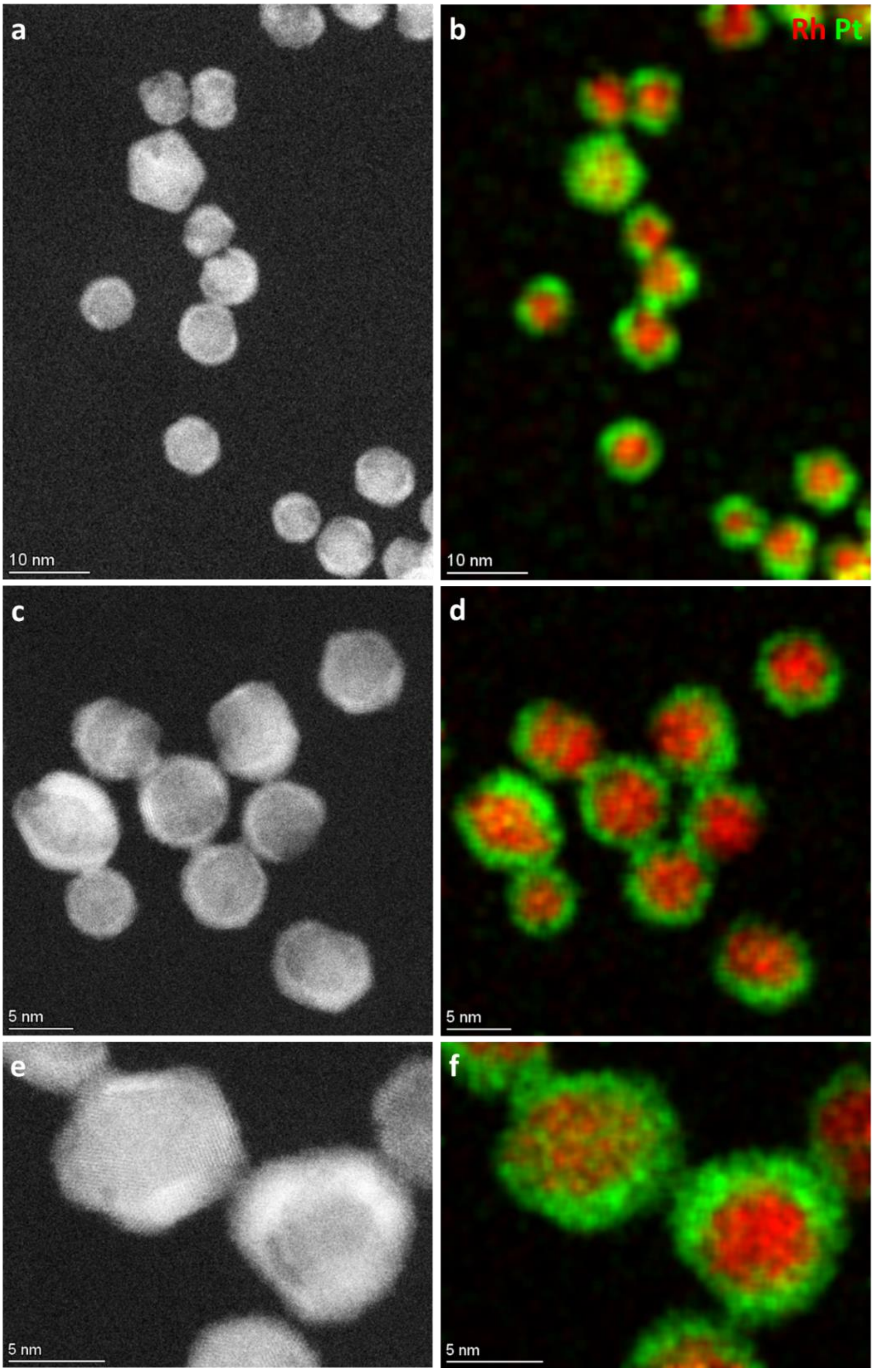
a
b
Rh Pt
10 nm
10 nm
c
d
5 nm
5 nm
e
f
5 nm
5 nm

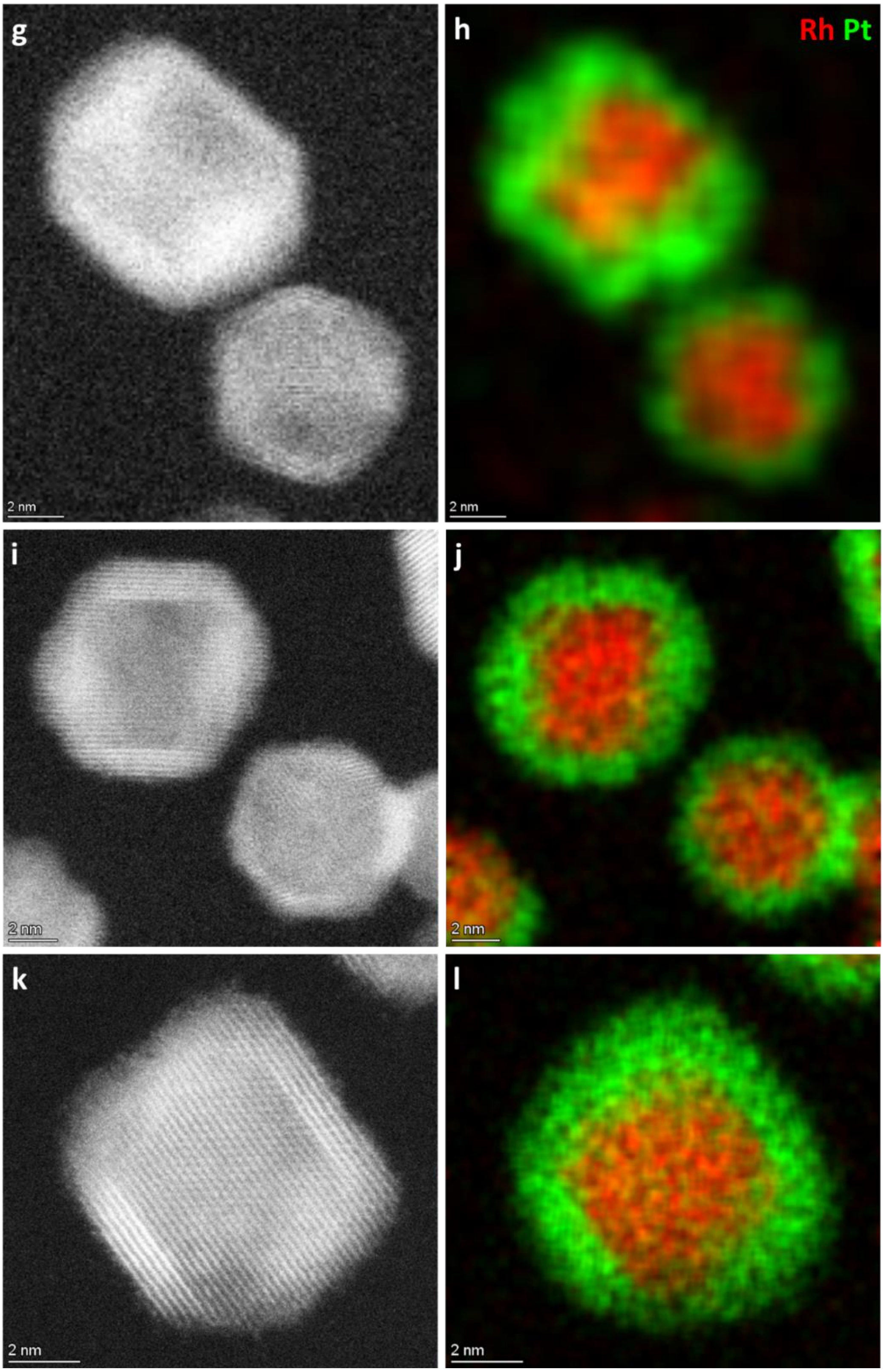
g
h
Rh Pt
2 nm
2 nm
i
j
2 nm
2 nm
k
l
2 nm
2 nm

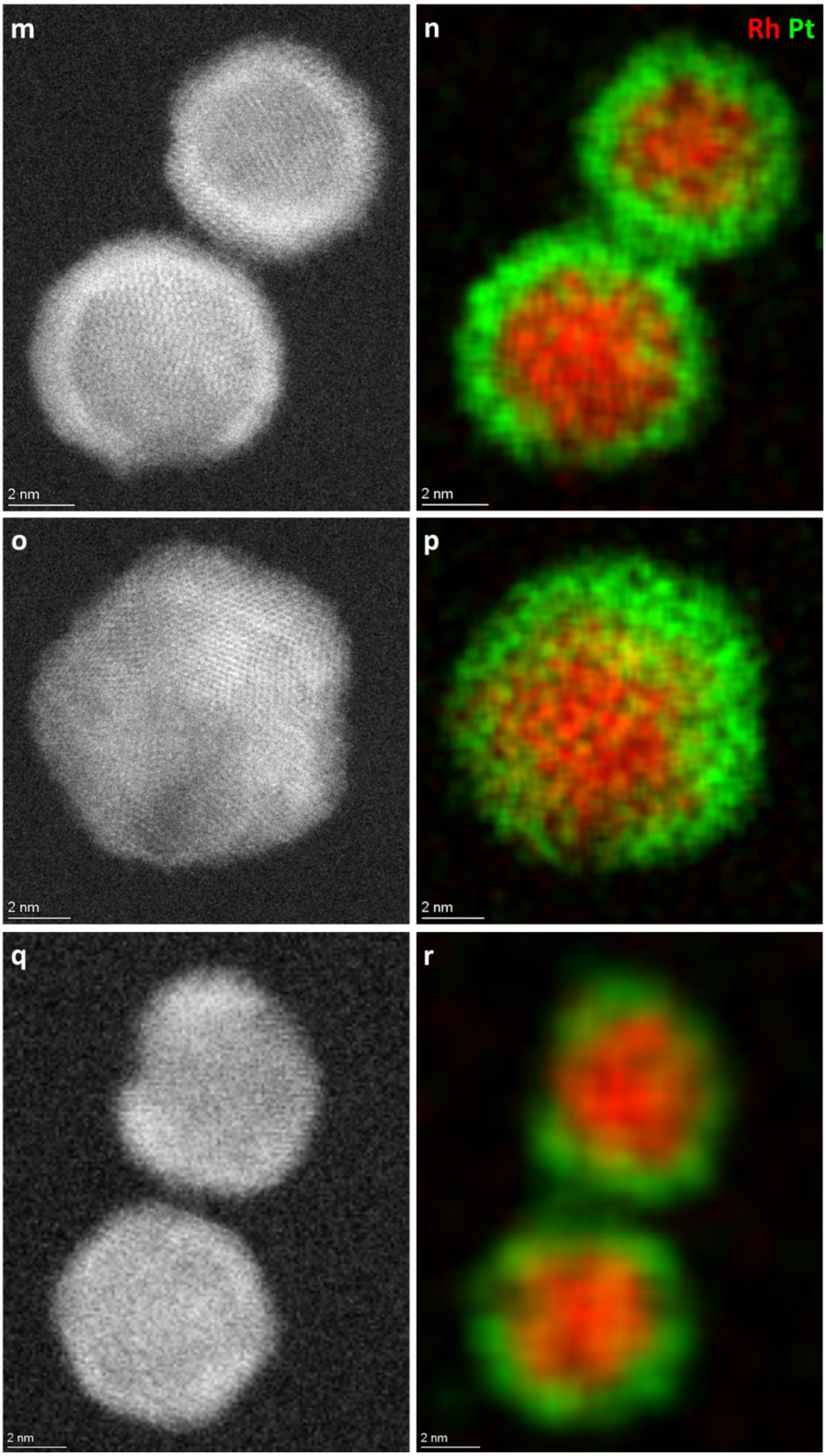

Fig. S2 Rh(core)-Pt(shell) nanoparticles imaged using HAADF-STEM and EDS elemental mapping at room temperature before heating to 650 °C. Rh and Pt are mapped in red and green, respectively.

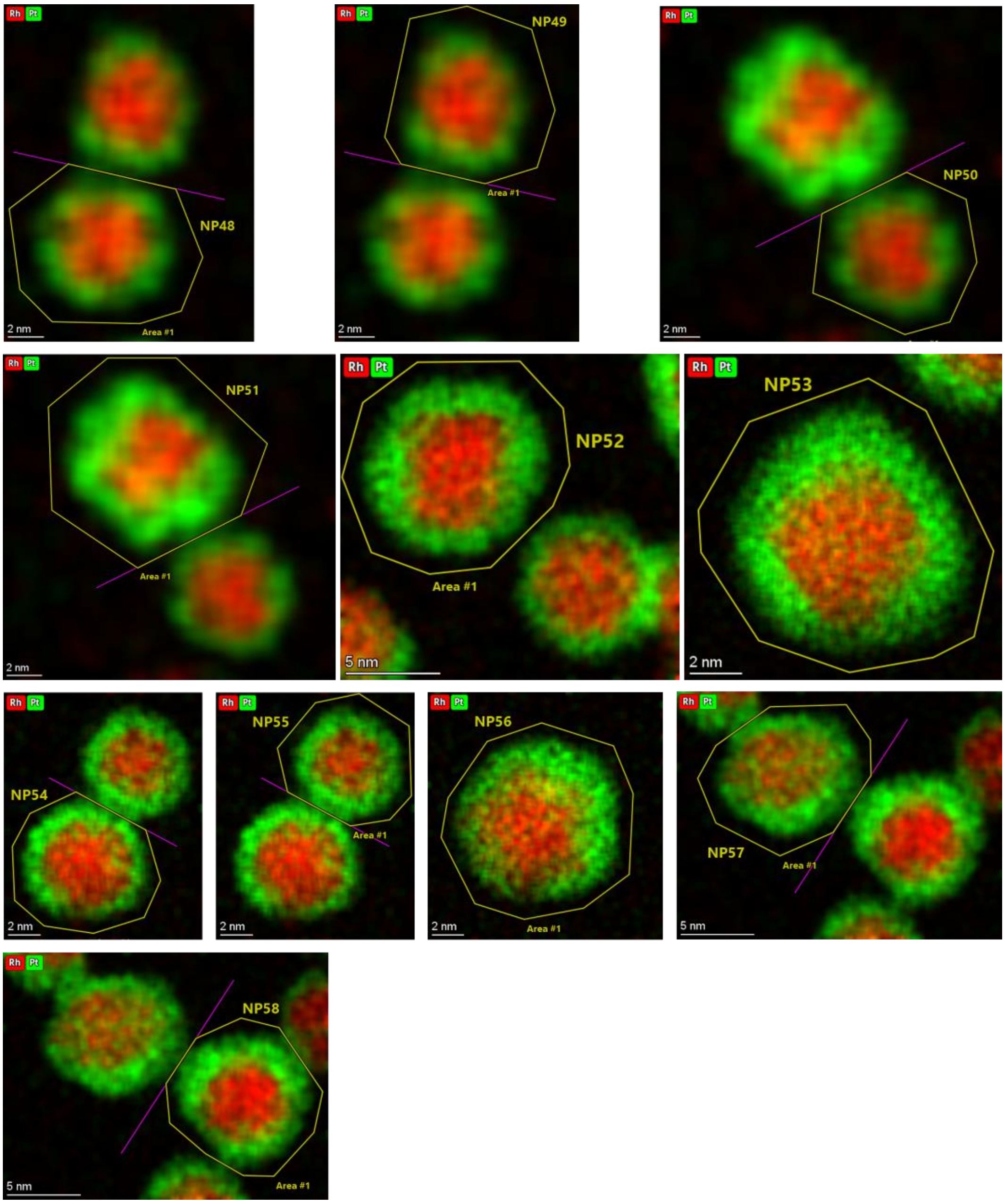

Fig. S3 EDS elemental maps of Rh(core)-Pt(shell) nanoparticles (NP48-58) at room temperature before heating to 650 °C, showing the areas selected for quantitative EDS analysis (results in Table S1). Rh and Pt are mapped in red and green, respectively.

Table S1 **Results from quantitative EDS analysis of Rh(core)-Pt(shell) nanoparticles (NP48-58) at room temperature before heating to 650 °C (see Fig. S3). The Pt-Rh molar composition is normalized to 100 at. %.**

Nanoparticle	Rh [at. %]	Pt [at. %]	Nanoparticle size [nm]
NP48	52 ±3.0	48 ±3.0	8.0
NP49	57 ±2.9	43 ±2.9	8.0
NP50	54 ±3.0	46 ±3.0	7.6
NP51	38 ±2.8	62 ±2.8	10.5
NP52	47 ±2.9	53 ±2.9	10.1
NP53	44 ±2.9	56 ±2.9	9.8
NP54	57 ±2.9	43 ±2.9	7.8
NP55	46 ±2.9	54 ±2.9	6.9
NP56	55 ±2.9	45 ±2.9	10.6
NP57	44 ±2.9	56 ±2.9	10.0
NP58	51 ±3.0	49 ±3.0	9.8

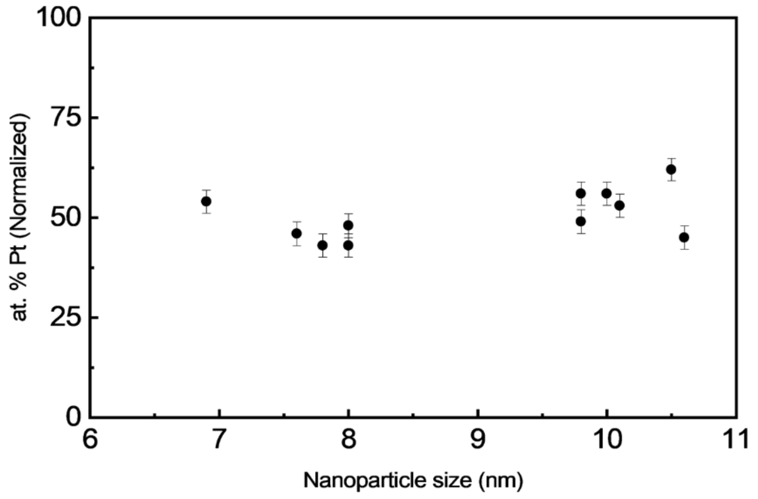

Fig. S4 **Normalized Pt content from quantitative EDS (Table S1) versus Rh(core)-Pt(shell) nanoparticle size at room temperature before heating to 650 °C. The Pt-Rh molar composition is normalized to 100 at. %.**

1.2. Rh(core)-Pt(shell) nanoparticles heated to 650 °C

The Rh(core)-Pt(shell) nanoparticles from section 1.1 were heated to 650 °C and kept at this temperature overnight (14 h) inside the TEM, before we instantly quenched them to room temperature. HAADF-STEM images and corresponding EDS elemental maps are shown as a function of time after room temperature was reached (Fig. S1, zone 4) in Fig. S5.

In total, 34 nanoparticles (D_{avg} = 9.0 ± 2.5 nm) were analyzed; see HAADF-STEM images and EDS maps in Fig. S5. The data show the great majority of the nanoparticles to be in the solid solution elemental distribution configuration, with complete mixing of Pt and Rh in 29 particles with D_{avg} = 8.4 ± 1.7 nm and size range 6 – 13 nm throughout the whole temporal range (24 min – 4 h 20 min) after quenching to room temperature. Four nanoparticles at time-points 0:44 (7.2 nm), 0:51 (11.6 nm), 1:49 (9.7 nm), and 2:19 (16.7 nm) show partial segregation (D_{avg} = 11.3 ± 4.0 nm), with some Rh-rich patches present inside the nanoparticles. The nanoparticle at time-point 3:29 (15.8 nm) is the sole nanoparticle in the total sample population showing clear segregation.

The chemical composition of a selection (NP59 – 76) of the individual Rh(core)-Pt(shell) nanoparticles was studied by quantitative EDS analysis as a function of time after quenching from 650 °C. The areas selected for analysis are shown in Fig. S6 and the results are summarized in Table S2. The average composition (±1σ) normalized to Pt and Rh was 43.6 ± 9.0 at. % Pt. The composition was then plotted versus nanoparticle size (Fig. S7a) to look for possible correlations between the two parameters. No correlations were identified. Next, we plotted the composition versus time progressed at room temperature after quenching from 650 °C (Fig. S7b). No correlation was identified between these two parameters. The type of elemental distribution, categorized as segregated (S), partly segregated (PS) and solid solution (SS), was plotted versus the chemical composition by the quantitative EDS analysis (Fig. S7c) and versus nanoparticle size (Fig. S7d). No correlation between elemental configuration and chemical composition was identified. The type of elemental distribution versus nanoparticle size was discussed in the beginning of this section and in the manuscript (section 1).

Overall, the HAADF-STEM and EDS elemental maps show the Rh(core)-Pt(shell) nanoparticles in the smallest size domain to transform to a solid solution elemental distribution configuration when heated to 650 °C for 14 h. The nanoparticles remained stable in this configuration upon quenching to and at room temperature during the time of characterization (4 h 20 min). Interestingly, we observe that larger nanoparticles segregate. These findings are independent of the chemical composition in the composition range studied.

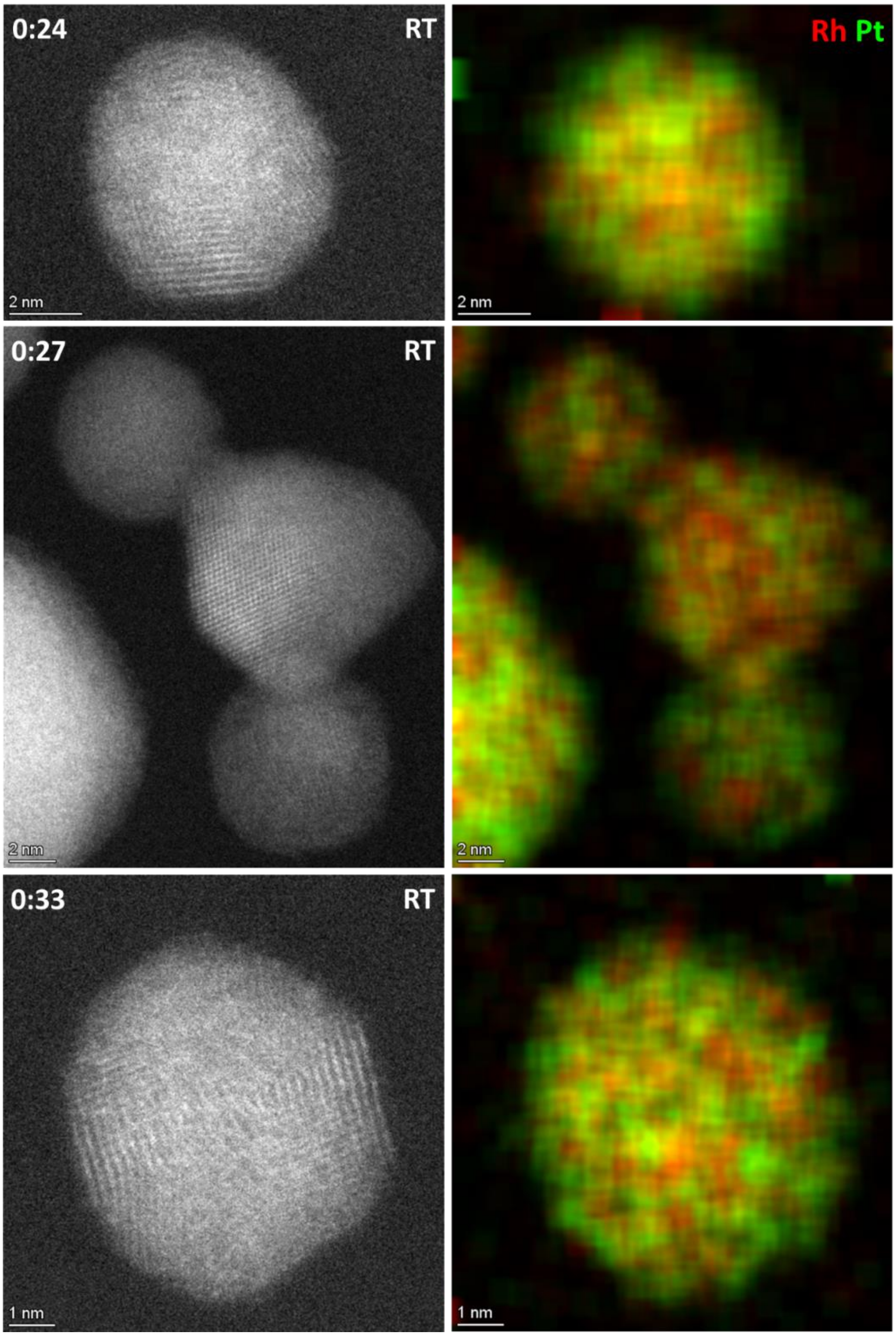
0:24
RT
2 nm
Rh Pt
2 nm
0:27
RT
2 nm
2 nm
0:33
RT
1 nm
1 nm

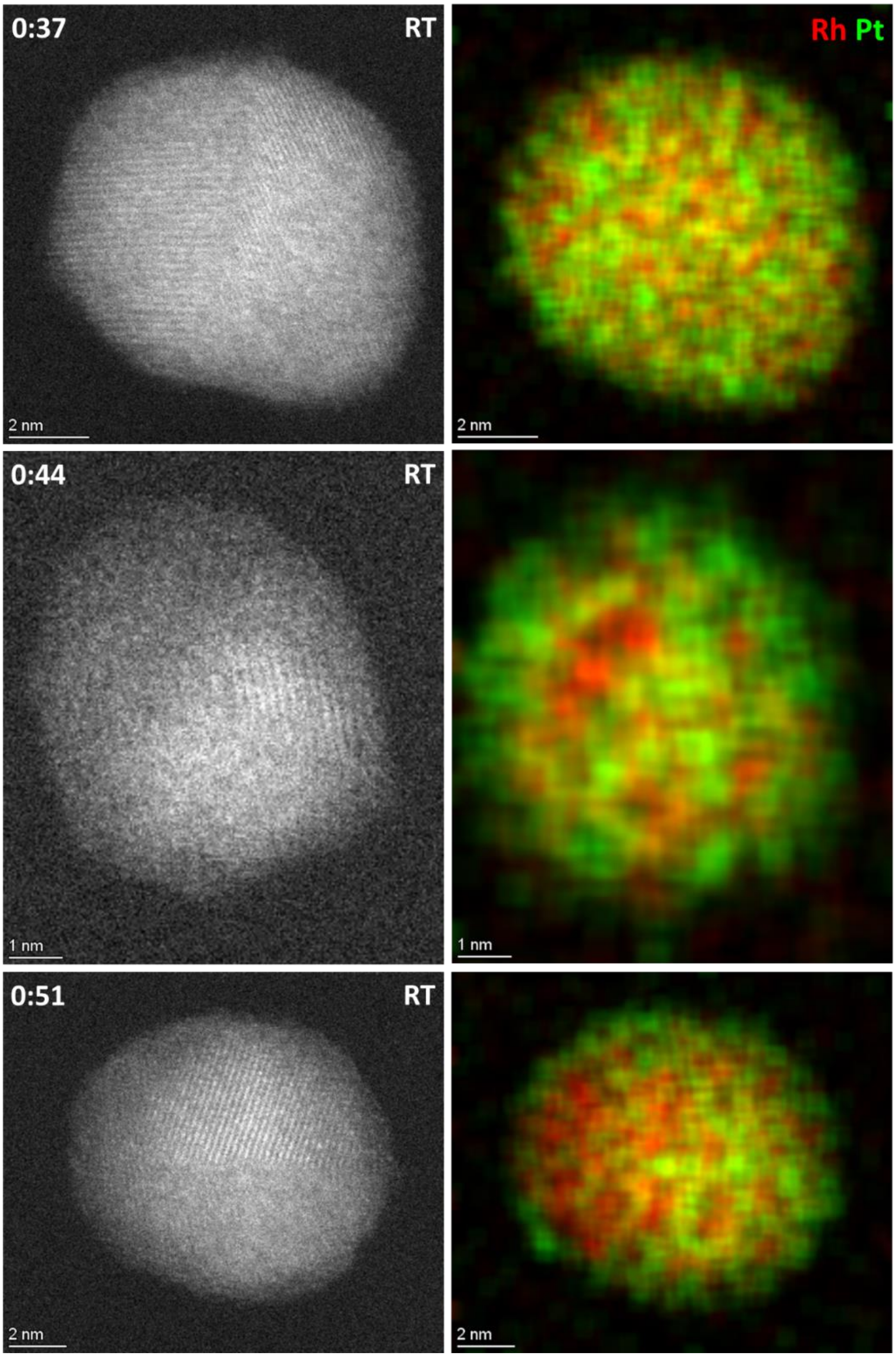
0:37
RT
2 nm
Rh Pt
2 nm
0:44
RT
1 nm
1 nm
0:51
RT
2 nm
2 nm

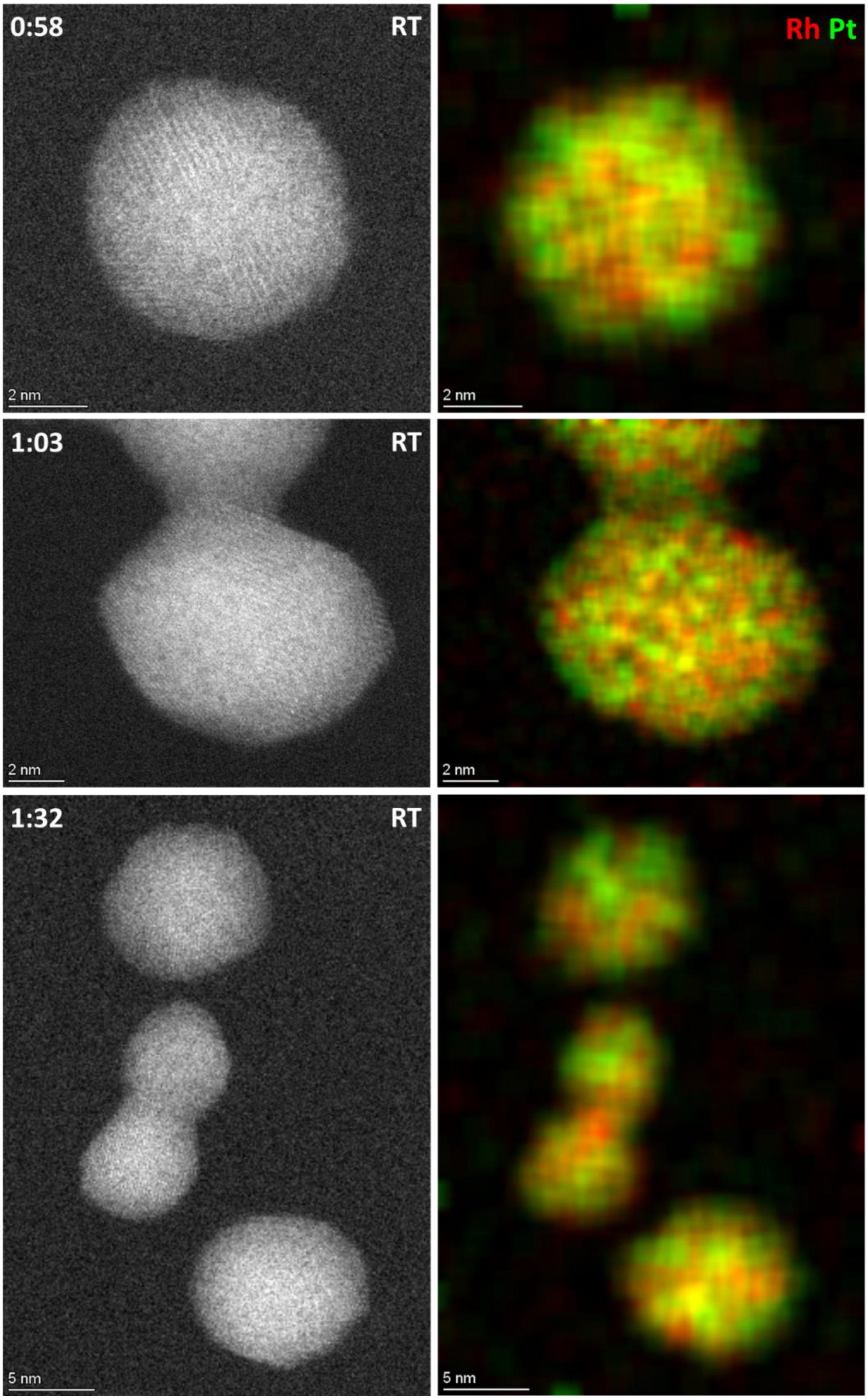
0:58
RT
Rh Pt
2 nm
2 nm
1:03
RT
2 nm
2 nm
1:32
RT
5 nm
5 nm

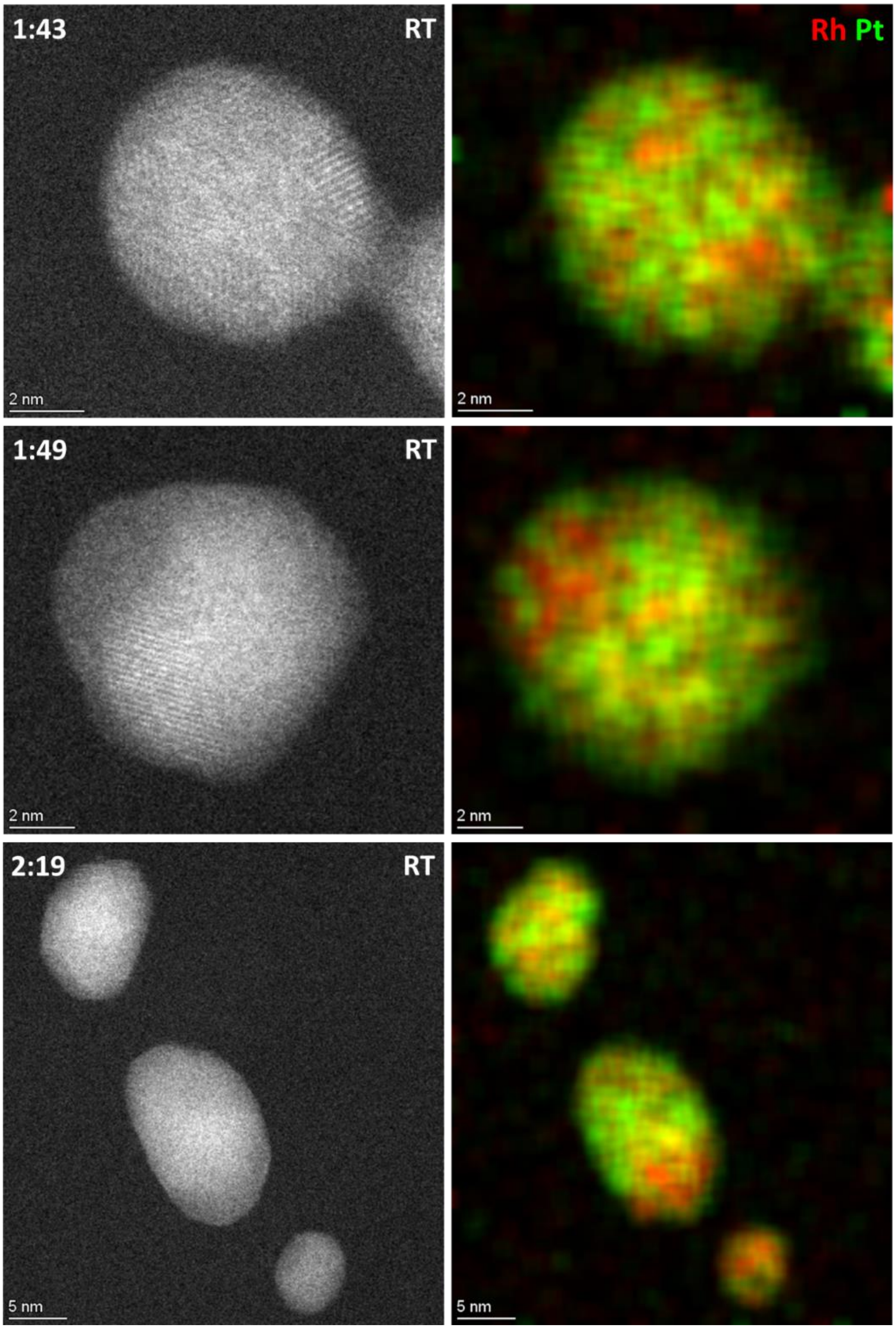
1:43
RT
2 nm
Rh Pt
2 nm
1:49
RT
2 nm
2 nm
2:19
RT
5 nm
5 nm

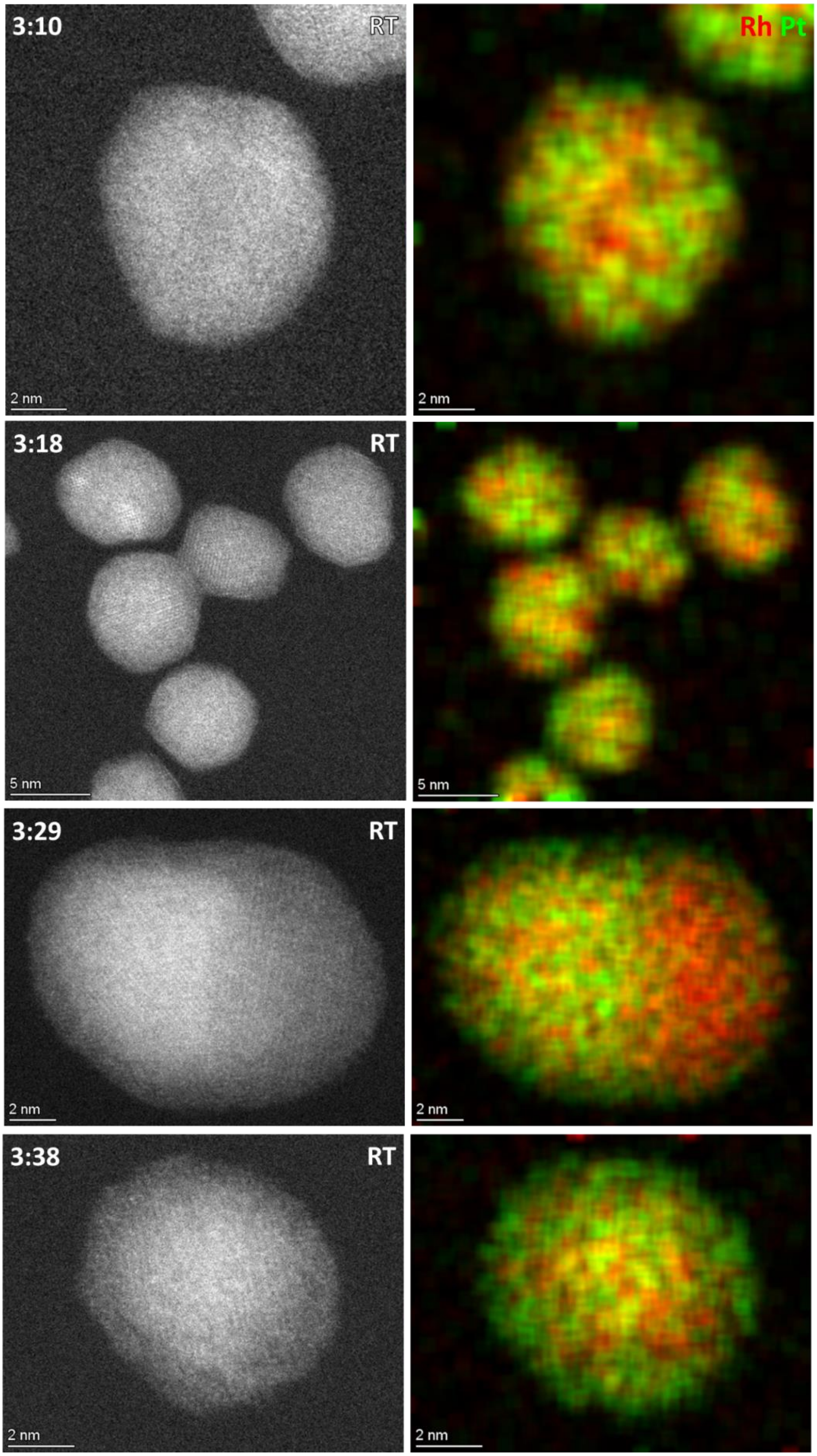
3:10
RT
Rh Pt
2 nm
2 nm
3:18
RT
5 nm
5 nm
3:29
RT
2 nm
2 nm
3:38
RT
2 nm
2 nm

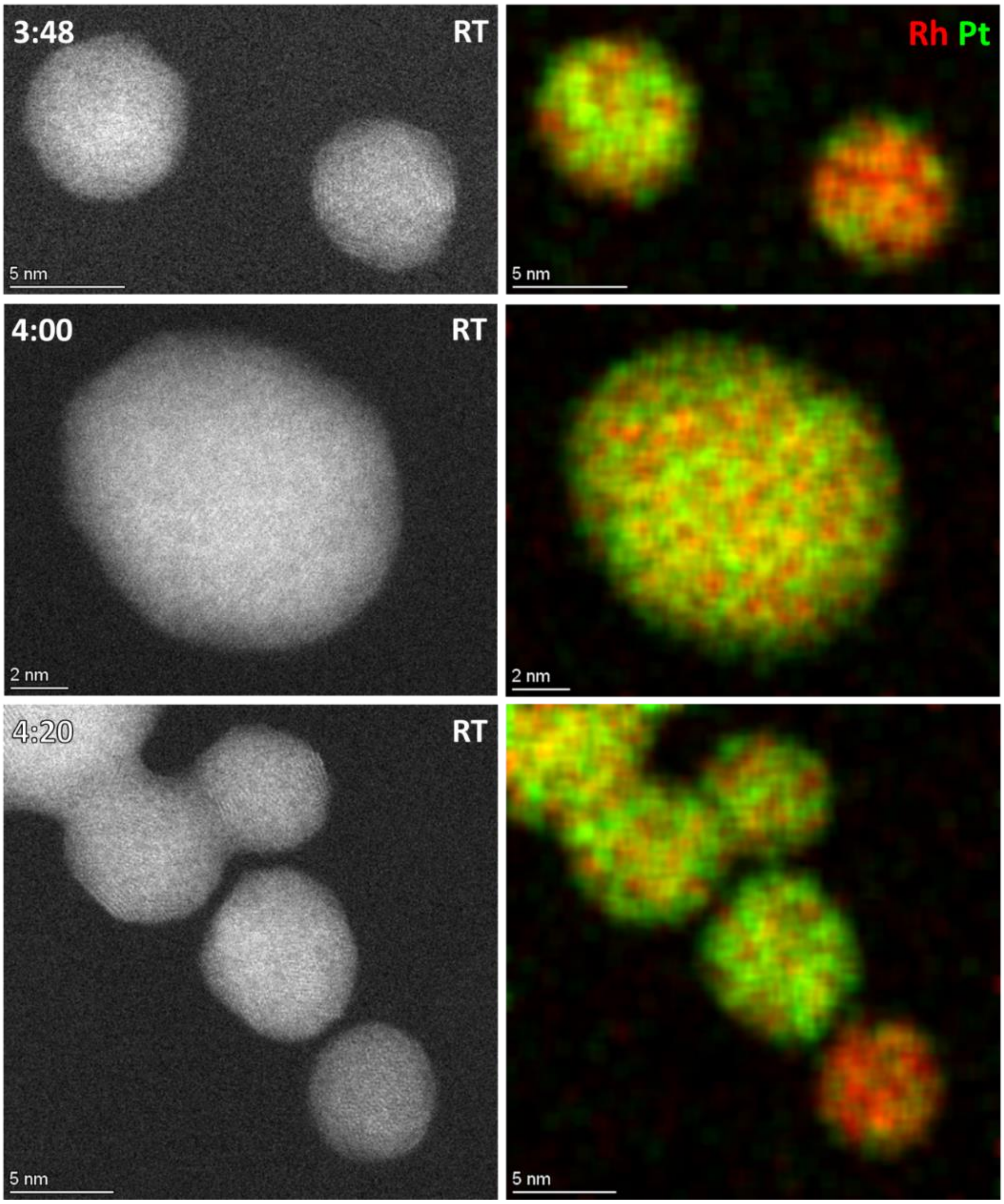

Fig. S5 **Rh(core)-Pt(shell) nanoparticles imaged using HAADF-STEM and EDS elemental maps at room temperature after quenching from 650 °C. The time after room temperature was reached is indicated by h:min. Rh and Pt are mapped in red and green, respectively.**

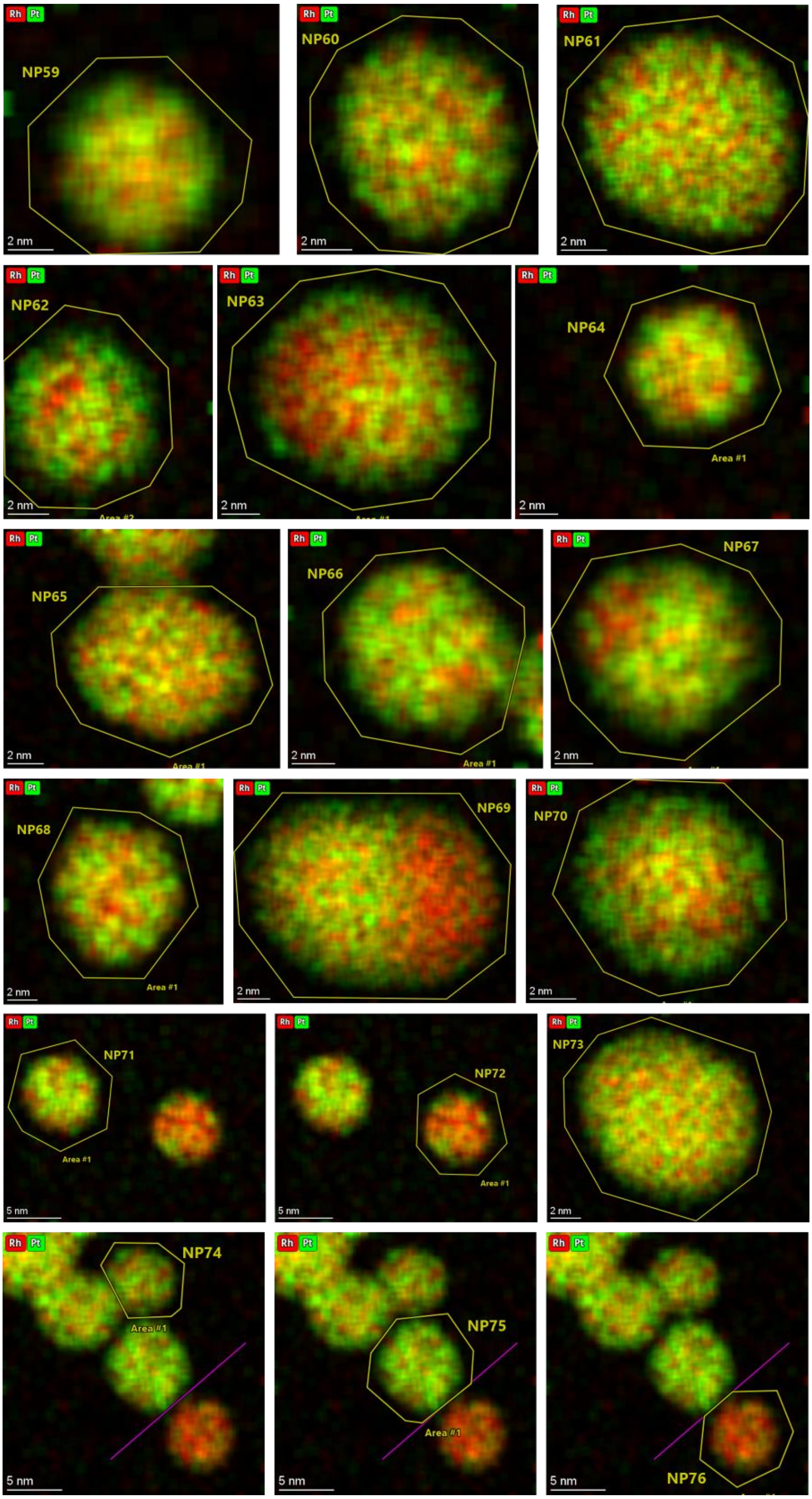

Fig. S6 **EDS elemental maps Rh(core)-Pt(shell) nanoparticles (batch 1) (NP59-76) at room temperature after quenching from 650 °C, showing the areas selected for quantitative EDS analysis (results in Table S2). Rh and Pt are mapped in red and green, respectively.**

Table S2 Results from quantitative EDS analysis of Rh(core)-Pt(shell) nanoparticles (batch 1) (NP59-76) at room temperature after quenching from 650 °C (see Fig. S6). The Pt-Rh molar composition is normalized to 100 at. %.

Nanoparticle	Time at RT [h: mm]	Rh [at. %]	Pt [at. %]	Nanoparticle size [nm]	Element distribution
NP59	0:24	54 ±3.0	46 ±3.0	7.6	Solid solution
NP60	0:33	55 ±2.9	45 ±2.9	8.1	Solid solution
NP61	0:37	56 ±2.9	44 ±2.9	10.2	Solid solution
NP62	0:44	46 ±2.9	54 ±2.9	7.2	Partly segregated
NP63	0:51	72 ±2.4	28 ±2.4	11.6	Partly segregated
NP64	0:58	52 ±3.0	48 ±3.0	7.0	Solid solution
NP65	1:03	54 ±2.9	46 ±2.9	10.9	Solid solution
NP66	1:43	54 ±2.9	46 ±2.9	7.8	Solid solution
NP67	1:49	46 ±2.9	54 ±2.9	9.7	Partly segregated
NP68	3:10	61 ±2.8	39 ±2.8	9.8	Solid solution
NP69	3:29	52 ±3.0	48 ±3.0	15.8	Segregated
NP70	3:38	54 ±2.9	46 ±2.9	8.4	Solid solution
NP71	3:48	63 ±2.8	37 ±2.8	7.6	Solid solution
NP72	3:48	75 ±2.2	25 ±2.2	6.6	Solid solution
NP73	4:00	52 ±2.9	48 ±2.9	12.6	Solid solution
NP74	4:20	51 ±3.0	49 ±3.0	6.5	Solid solution
NP75	4:20	46 ±2.9	54 ±2.9	8.6	Solid solution
NP76	4:20	73 ±2.3	27 ±2.3	6.8	Solid solution

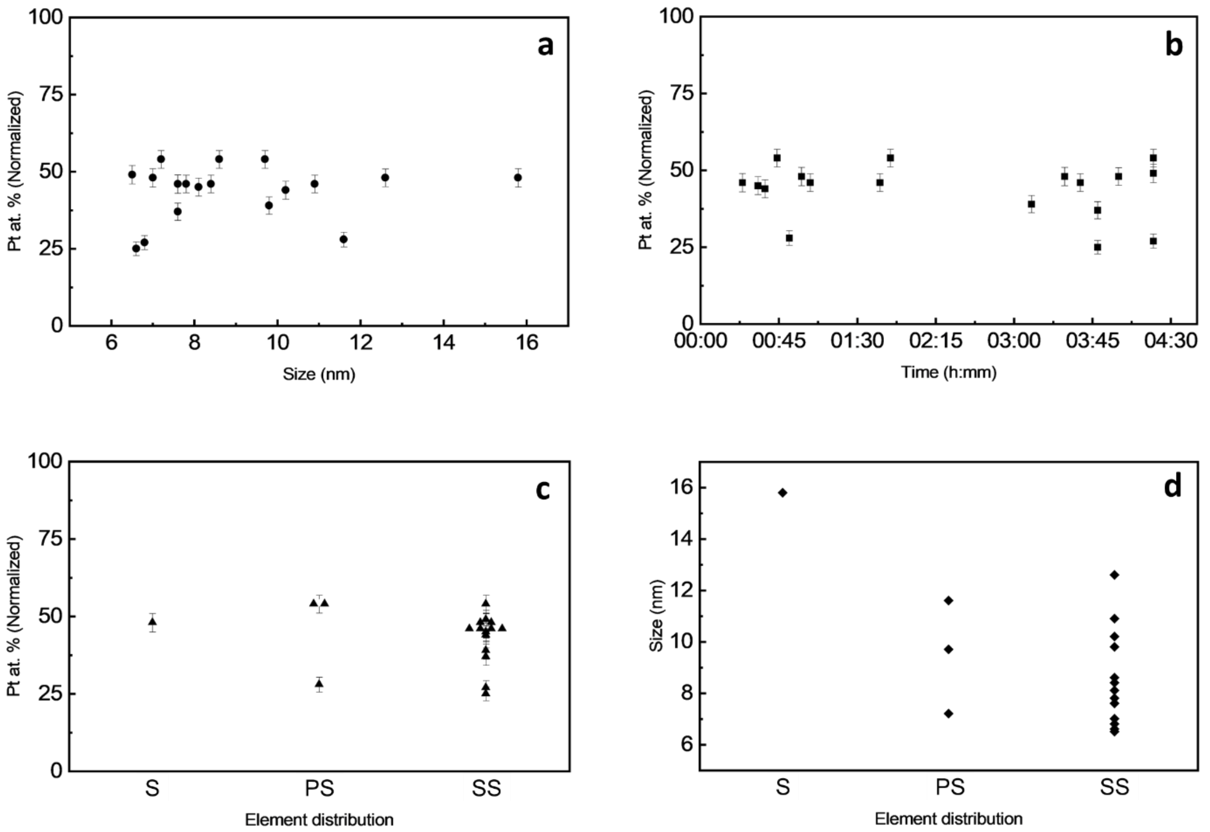

Fig. S7 Normalized Pt-content from quantitative EDS (Table S2) versus Rh(core)-Pt(shell) nanoparticle size (a), time (hh:mm) at room temperature after quenching (b), elemental distribution type (c), and nanoparticle size versus elemental distribution type (d) at room temperature after quenching from 650 °C. The Pt-Rh molar composition is normalized to 100 at. %. The elemental distribution type is categorized as segregated (S), partly segregated (PS), and solid solution (SS).

Justification for particle selection in Fig. 4 (main manuscript)

We wanted to avoid beam effects affecting the elemental distribution in the nanoparticles imaged at 650 °C by imaging the sample at lower magnification (Fig. S1, zone 3), see overview HAADF-STEM images in Fig. 3 in the manuscript. We observed some of the larger nanoparticles to be fully segregated, although solid solution particles existed in the full nanoparticle size-range. When correlating the nanoparticle size to the segregation behavior, we decided to only measure the size of the nanoparticles that were clearly segregated in the overview HAADF-images at low magnification for the following reason: We observed a lower limit in the nanoparticle size corresponding to segregated elemental distribution (~ 13 nm). The observation could be explained by two reasons or a combination of these: (i) the smaller nanoparticles were stable against segregation in the solid solution configuration, (ii) the resolution in the lower magnification overview HAADF-STEM image (Fig. 3 in the manuscript) was too low to identify segregated nanoparticles below a certain size, i.e., below a certain particle size limit all particles appear to be of the solid solution configuration. If one includes the size measurements of the solid solution particles in the analysis based on the low magnification HAADF-images, there is a risk of including segregated particles falsely appearing as solid solution particles due to the lower resolution. In the next part of the experiment (Fig. S1, zone 4), we studied smaller particles, including those below the lower size limit of segregation, at room temperature after quenching the sample (freezing the state from 650 °C) where beam effects are not an issue. With the room temperature data, we show that smaller particles were stable in the solid solution configuration (i). Therefore, to evaluate the segregation behavior as a function of particle size at 650 °C, the sizes of the segregated particles from the overview HAADF images at 650 °C are plotted together with the sizes of the smaller particles as obtained from the high-resolution HAADF-images and corresponding EDS maps of the quenched sample at room temperature (Fig. S5, section 1.2).

2. In-situ Transmission Electron Microscopy study of Pt-Rh solid solution nanoparticles heated to 600 °C

This experiment was performed to elaborate on our hypothesis that Pt-Rh nanoparticles ≲ 13 nm are stable in the solid solution configuration over the full temperature range 25 – 650 °C. The as-synthesized Pt-Rh solid solution nanoparticles were heated according to the temperature program in Fig. S8 (also shown in the manuscript, Fig. 5). The average size of all nanoparticles analyzed in the temperature range 600 °C – RT was 8.9 ± 2.0 nm. In the experiment, a maximum temperature of 600 °C was chosen for in-situ high-resolution HAADF-STEM imaging and EDS mapping while avoiding issues with electron beam damage.

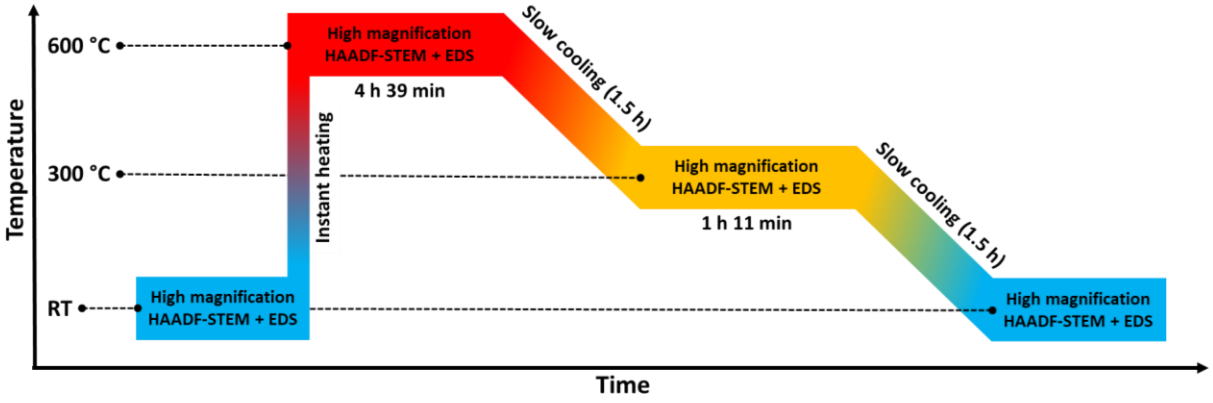

Fig. S8 Temperature program used in the in-situ TEM heating experiments of the Pt-Rh solid solution nanoparticles heated to 600 °C and stepwise cooled to room temperature.

2.1. Solid solution nanoparticles at room temperature before heating

An analysis of the Pt-Rh solid solution nanoparticles at room temperature was carried out before the sample was heated to 600 °C in vacuum. In this section, we examine the elemental distribution from HAADF-STEM images and EDS maps at room temperature (Fig. S9), and conclude that the sample is homogeneous - all nanoparticles show the solid solution elemental distribution.

The chemical composition of a selection (NP77 – 105) of individual Pt-Rh nanoparticles was determined by quantitative EDS analysis. The areas selected for analysis are shown in Fig. S10 and the results are summarized in Table S3. The chemical composition is plotted versus nanoparticle size (Fig. S11) to show the sample homogeneity with respect to chemical composition. The average composition of the nanoparticles (± 1σ) normalized to Pt and Rh is 46.3 ± 4.3 at.% Pt.

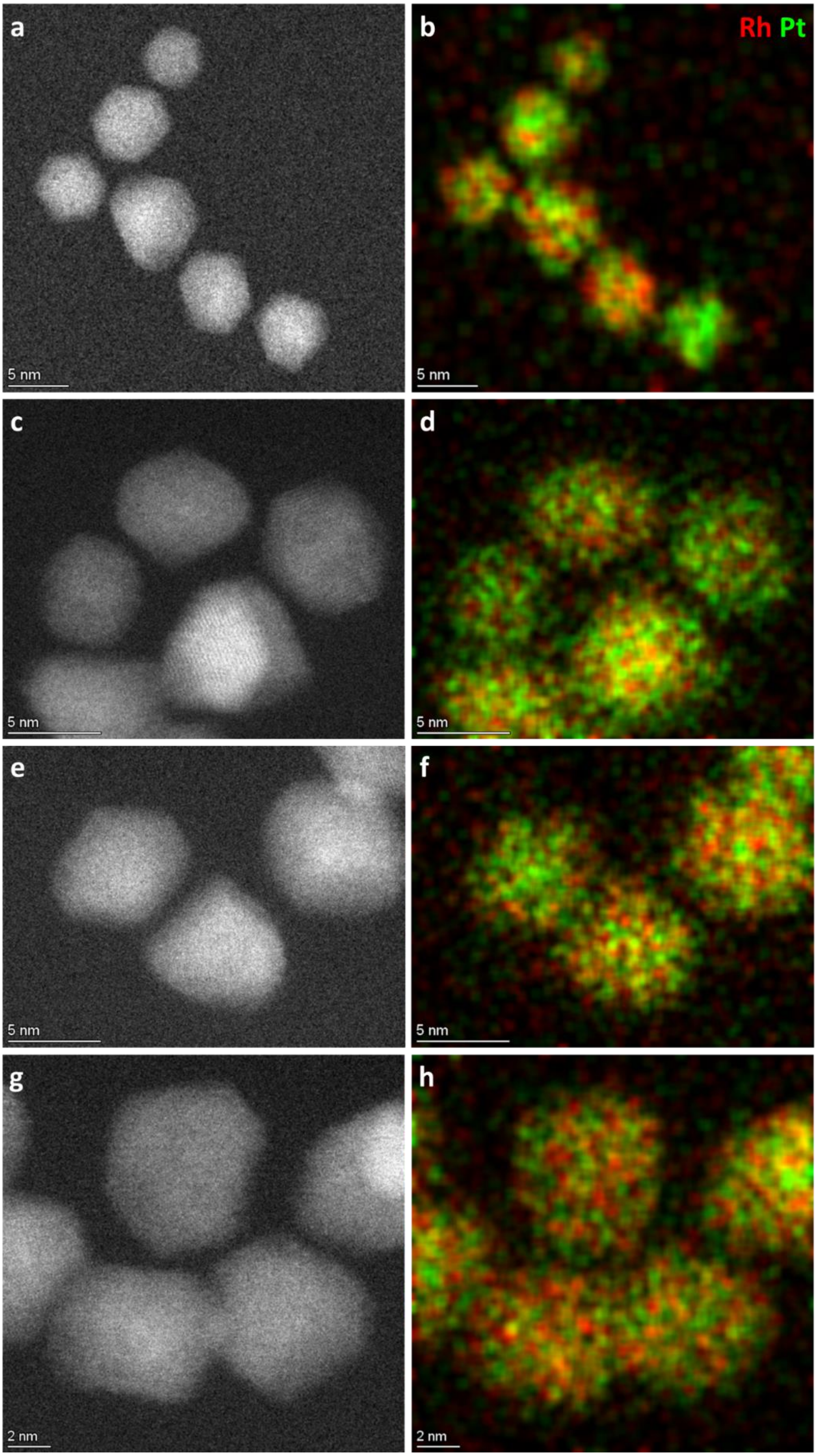
a
b
Rh Pt
c
d
e
f
g
h
5 nm
2 nm

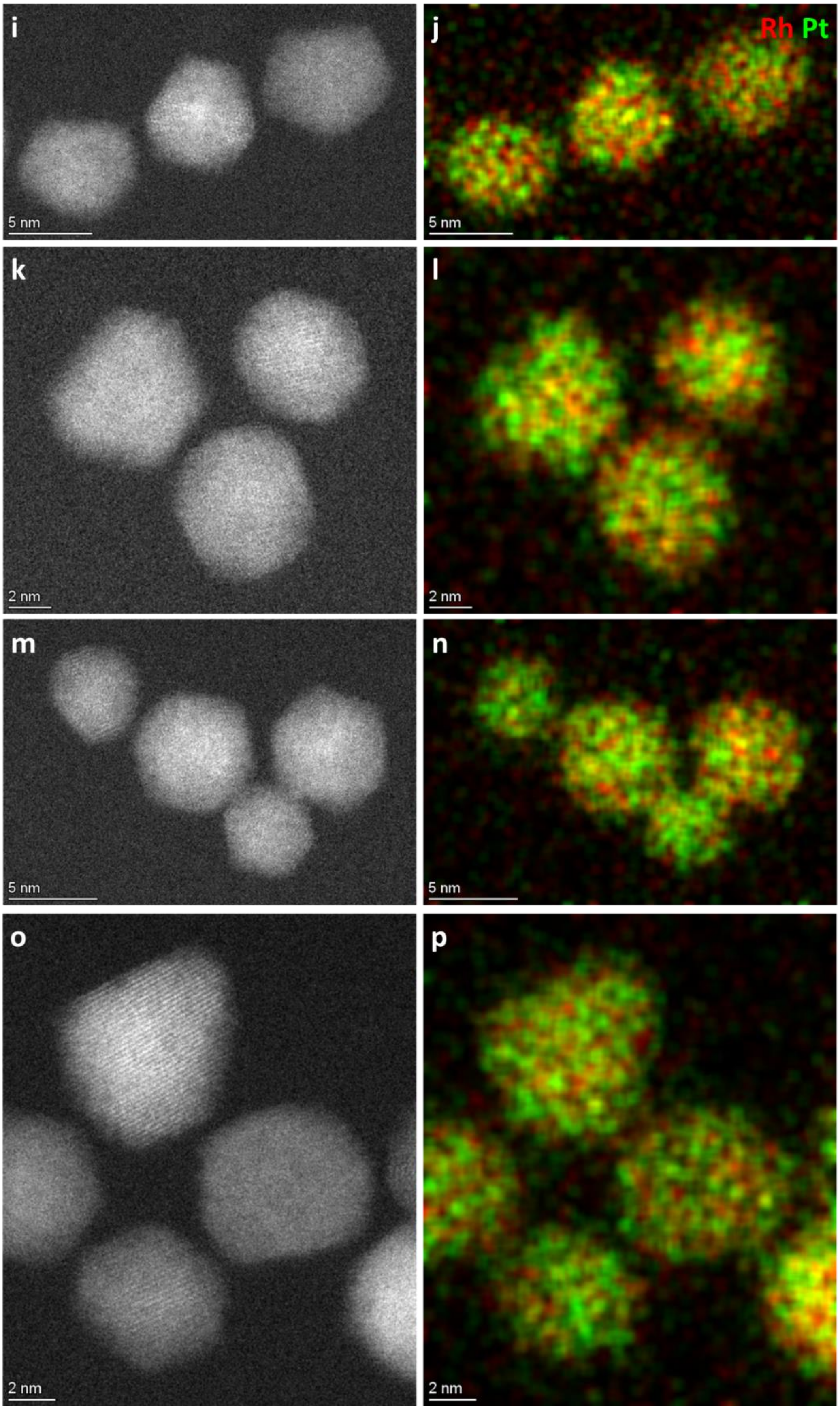
i
j
Rh Pt
5 nm
5 nm
k
l
2 nm
2 nm
m
n
5 nm
5 nm
o
p
2 nm
2 nm

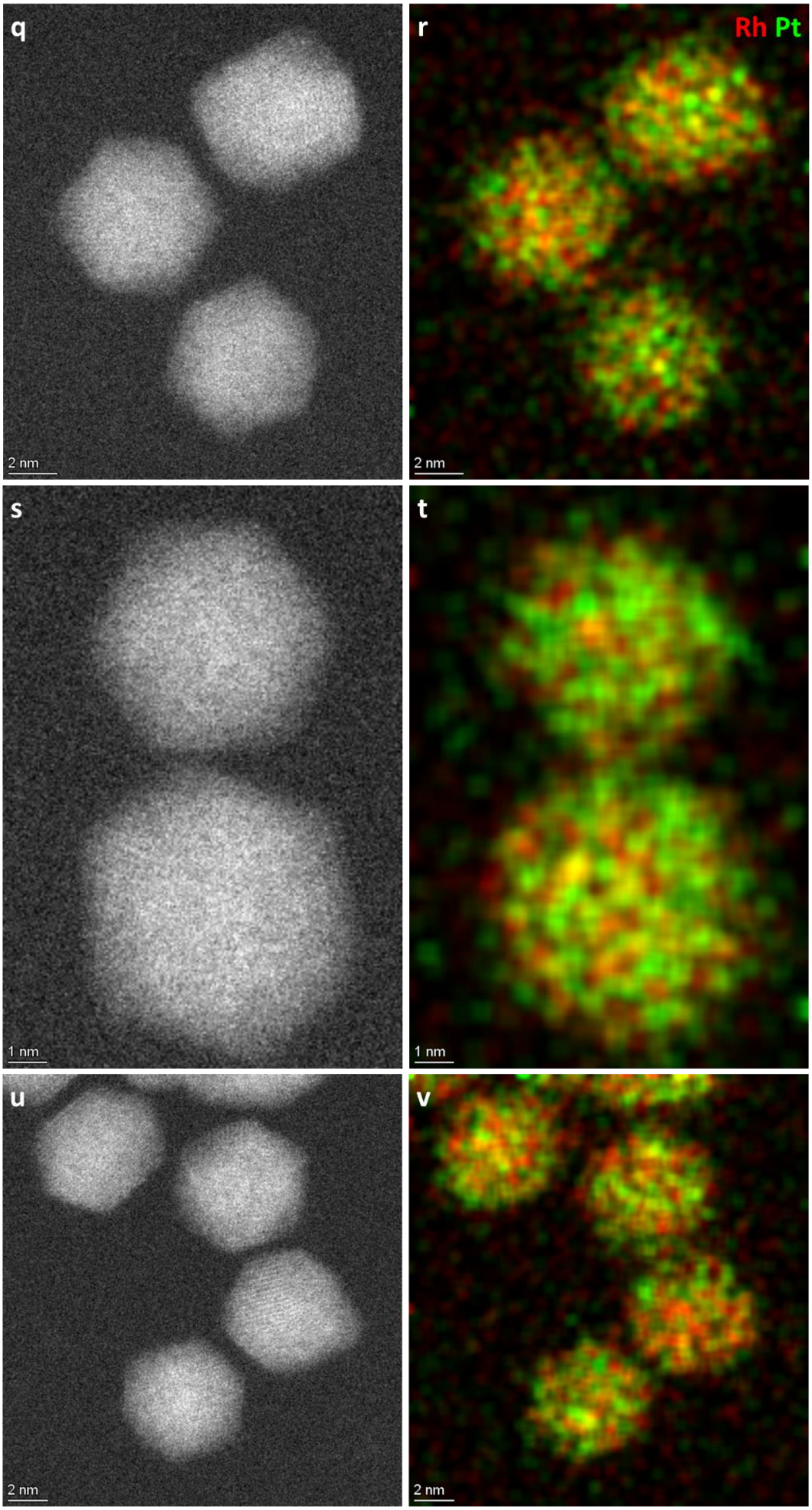
q
r
Rh Pt
2 nm
2 nm
s
t
1 nm
1 nm
u
v
2 nm
2 nm

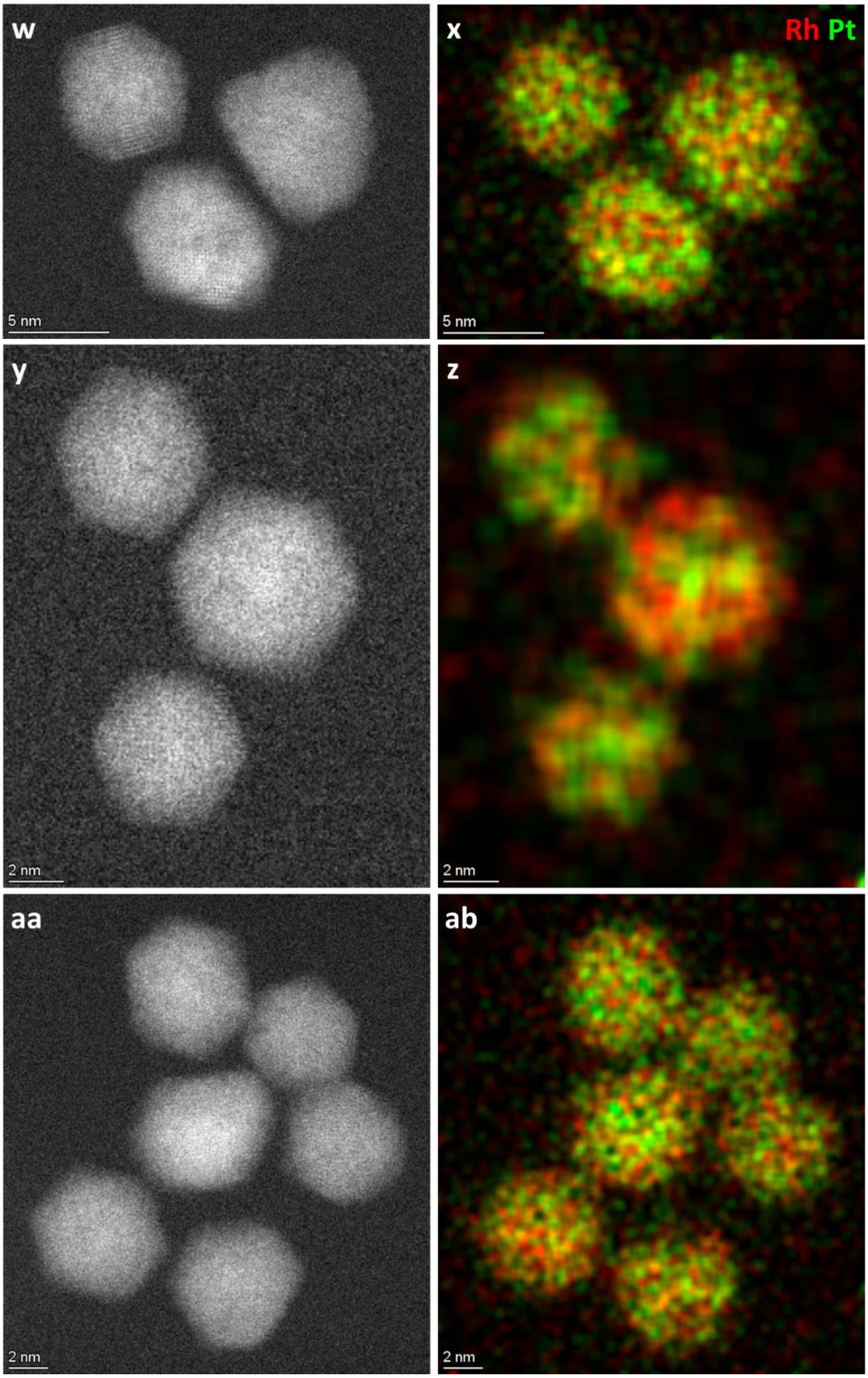
w
x
Rh Pt
5 nm
5 nm
y
z
2 nm
2 nm
aa
ab
2 nm
2 nm

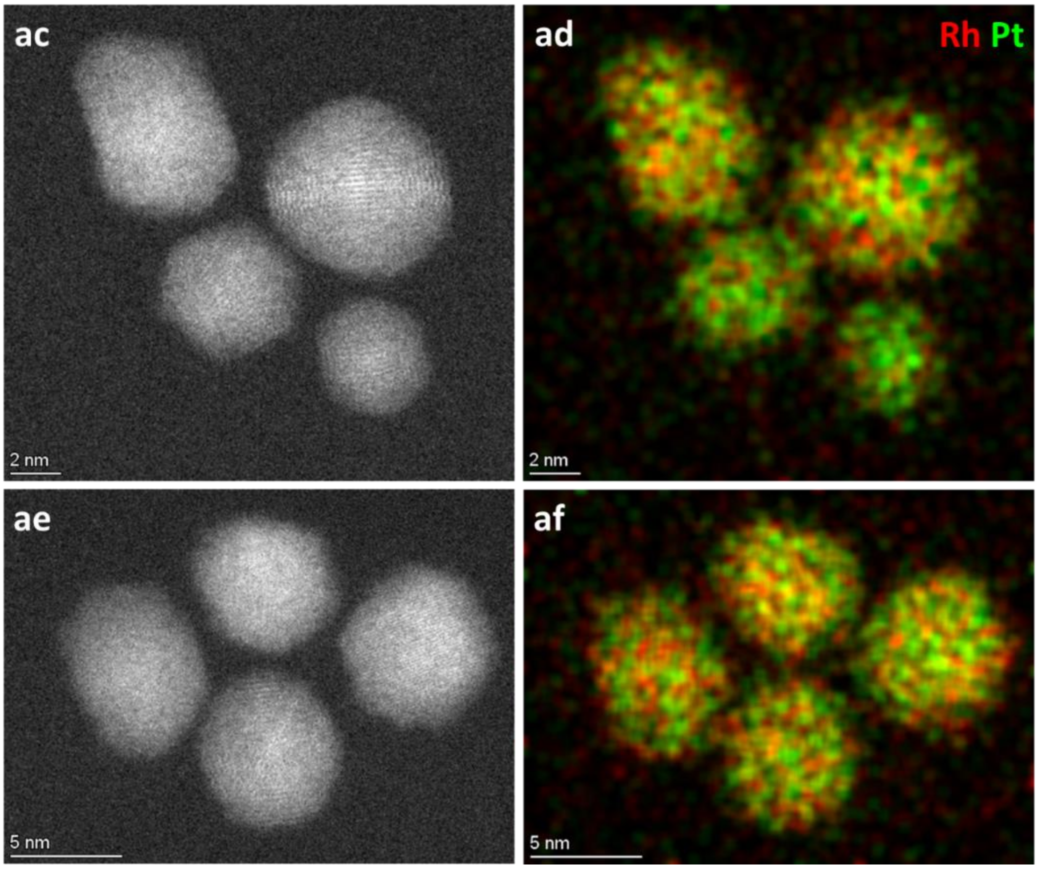

Fig. S9 **Pt-Rh solid solution nanoparticles imaged with HAADF-STEM and EDS elemental mapping at room temperature before heating to 600 °C. Rh and Pt are mapped in red and green, respectively.**

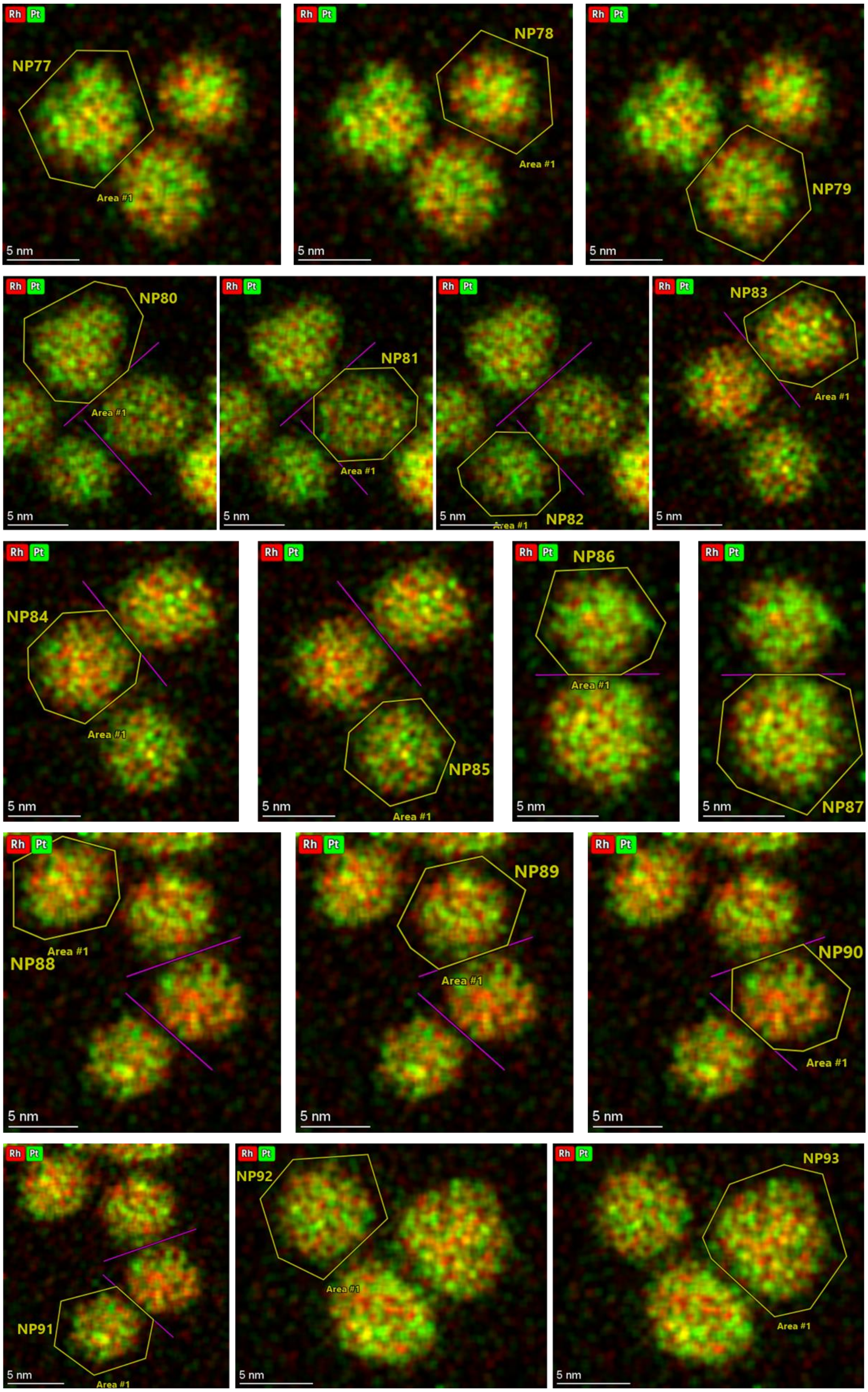

Rh
Pt
NP77
Area #1
5 nm
NP78
NP79
NP80
NP81
NP82
NP83
NP84
NP85
NP86
NP87
NP88
NP89
NP90
NP91
NP92
NP93

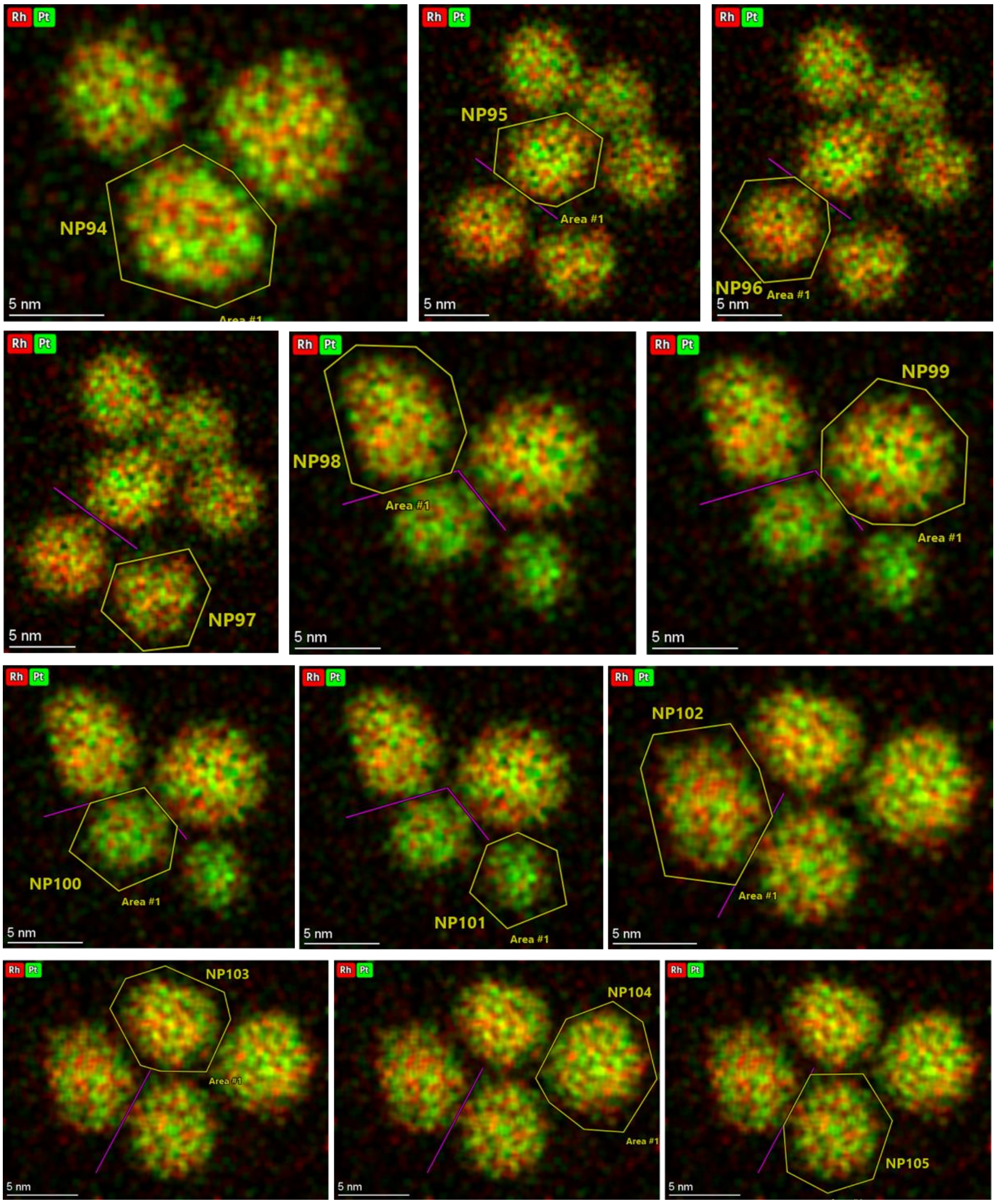

Fig. S10 EDS elemental maps of Pt-Rh solid solution nanoparticles (NP77-105) at room temperature before heating to 600 °C, showing the areas selected for quantitative EDS analysis (results in Table S3). Rh and Pt are mapped in red and green, respectively.

Table S3 Results from quantitative EDS analysis of Pt-Rh solid solution nanoparticles (NP77-105) at room temperature before heating to 600 °C (see Fig. S10). The Pt-Rh molar composition is normalized to 100 at. %.

Nanoparticle	Rh [at. %]	Pt [at. %]	Nanoparticle size [nm]
NP77	46 ±3.0	54 ±3.0	7.8
NP78	55 ±2.9	45 ±2.9	6.6
NP79	52 ±3.0	48 ±2.0	7.4
NP80	55 ±2.9	45 ±2.9	9.2
NP81	57 ±2.9	43 ±2.9	7.7
NP82	50 ±3.0	50 ±3.0	6.7
NP83	51 ±3.0	49 ±3.0	7.1
NP84	54 ±3.0	46 ±3.0	6.8
NP85	46 ±3.0	54 ±3.0	6.6
NP86	58 ±2.9	42 ±2.9	6.4
NP87	61 ±2.8	39 ±2.8	7.8
NP88	53 ±3.0	47 ±3.0	7.0
NP89	53 ±3.0	47 ±3.0	7.1
NP90	58 ±2.9	42 ±3.0	7.1
NP91	49 ±3.0	41 ±3.0	6.5
NP92	57 ±2.9	43 ±2.9	7.1
NP93	58 ±2.9	42 ±2.9	8.7
NP94	57 ±2.9	43 ±2.9	8.5
NP95	52 ±3.0	48 ±3.0	7.5
NP96	57 ±2.9	43 ±2.9	7.3
NP97	53 ±3.0	47 ±3.0	6.9
NP98	55 ±2.9	45 ±3.0	8.1
NP99	56 ±2.9	44 ±2.9	7.3
NP100	47 ±3.0	53 ±3.0	5.7
NP101	43 ±3.0	57 ±3.0	4.7
NP102	58 ±2.9	42 ±2.9	7.9
NP103	54 ±2.9	46 ±2.9	6.8
NP104	52 ±3.0	48 ±3.0	7.5
NP105	51 ±3.0	49 ±3.0	7.0

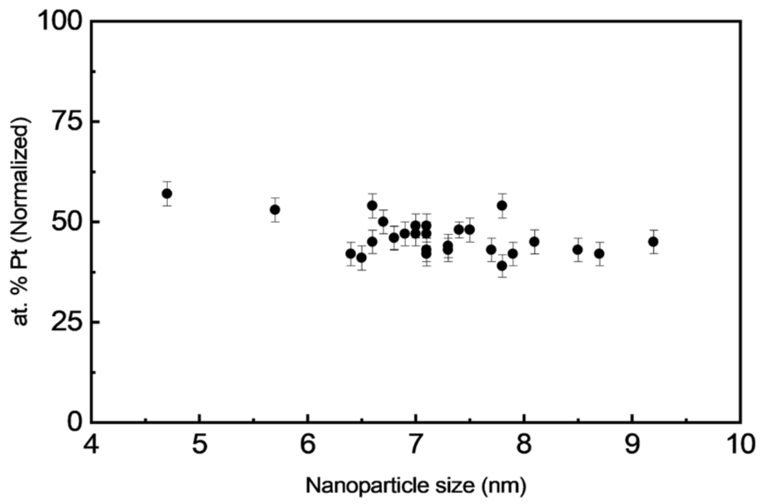

Fig. S11 Normalized Pt-content from quantitative EDS (Table S3) versus Pt-Rh solid solution nanoparticle size at room temperature before heating to 600 °C. The Pt-Rh molar composition is normalized to 100 at. %.

2.2. Pt-Rh solid solution nanoparticles heated to 600 °C

The Pt-Rh solid solution nanoparticles from section 2.1 were quickly ramped to 600 °C for prolonged dwelling (see temperature program in Fig. S8). HAADF-STEM images and EDS elemental maps were acquired at selected time points after reaching 600 °C; see Fig. S12.

The HAADF-STEM images and corresponding EDS maps in Fig. S12 show all the 18 imaged nanoparticles (D_{avg} = 8.3 ± 1.9 nm, size range 6 – 12 nm) to be in the solid solution elemental distribution configuration. These results show that the sample has not undergone any changes in the elemental distribution during the dwell time at 600 °C.

The chemical composition of a selection (NP106 – 119) of individual Pt-Rh nanoparticles was studied by quantitative EDS analysis as time progressed after reaching 600 °C. The areas selected for analysis are shown in Fig. S13 and the results are summarized in Table S4. The average composition (± 1σ) of nanoparticles NP106 – 119 normalized to Pt and Rh, is 47.5 ± 3.7 at. % Pt. Thereafter, the Pt content of the individual nanoparticles was plotted versus nanoparticle size (Fig. S14a), as well as the Pt content of individual nanoparticles versus dwell time at 600 °C (Fig. S14b). No correlations were identified.

Overall, the HAADF-STEM and EDS elemental maps show the Pt-Rh solid solution nanoparticles (D_{avg} = 8.3 ± 1.9 nm with size range 6 – 12 nm) to remain unchanged when heated and annealed at 600 °C (4 h 28 min), in the nanoparticle size- and chemical composition range studied.

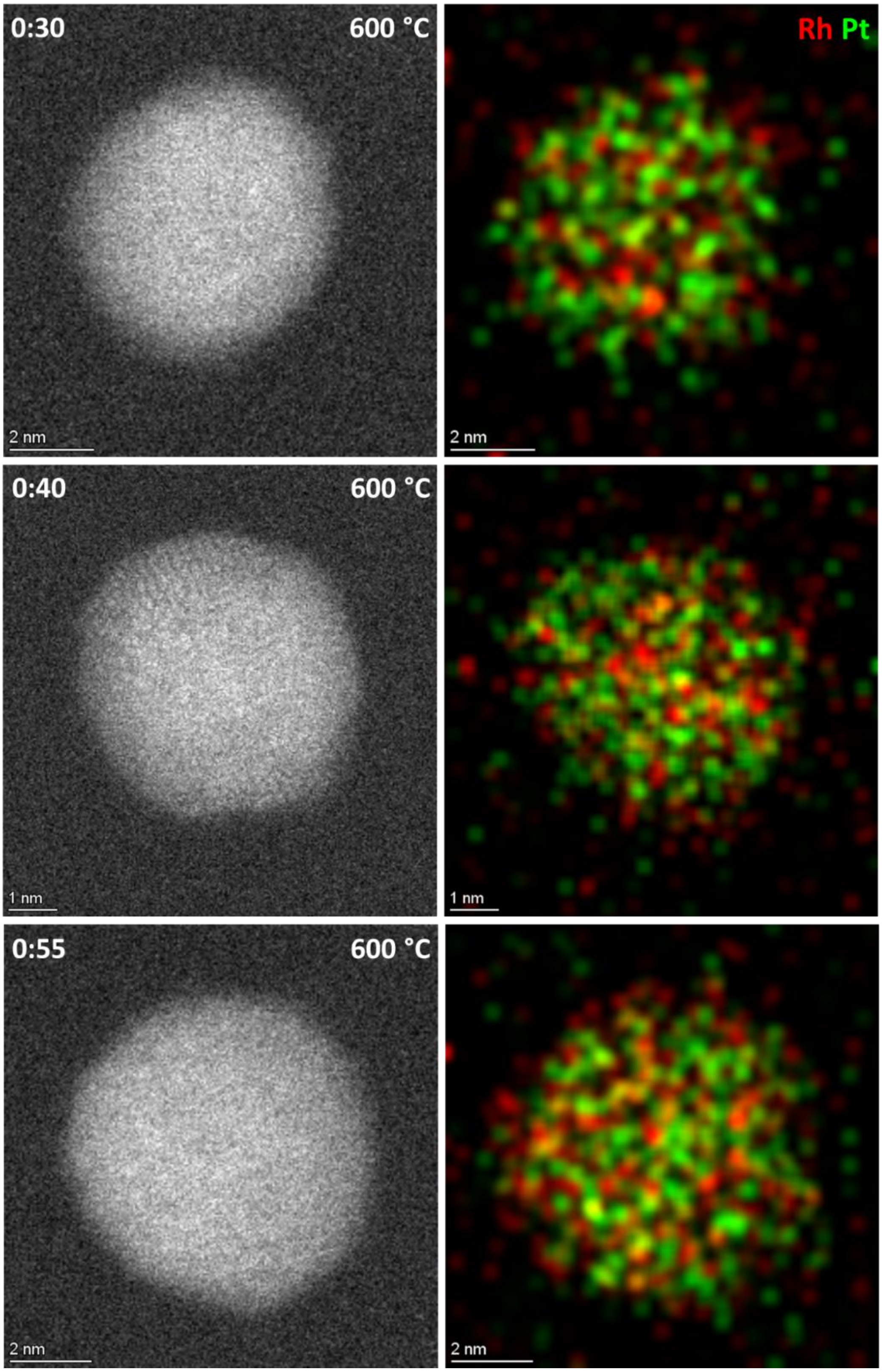
0:30
600 °C
Rh Pt
2 nm
2 nm
0:40
600 °C
1 nm
1 nm
0:55
600 °C
2 nm
2 nm

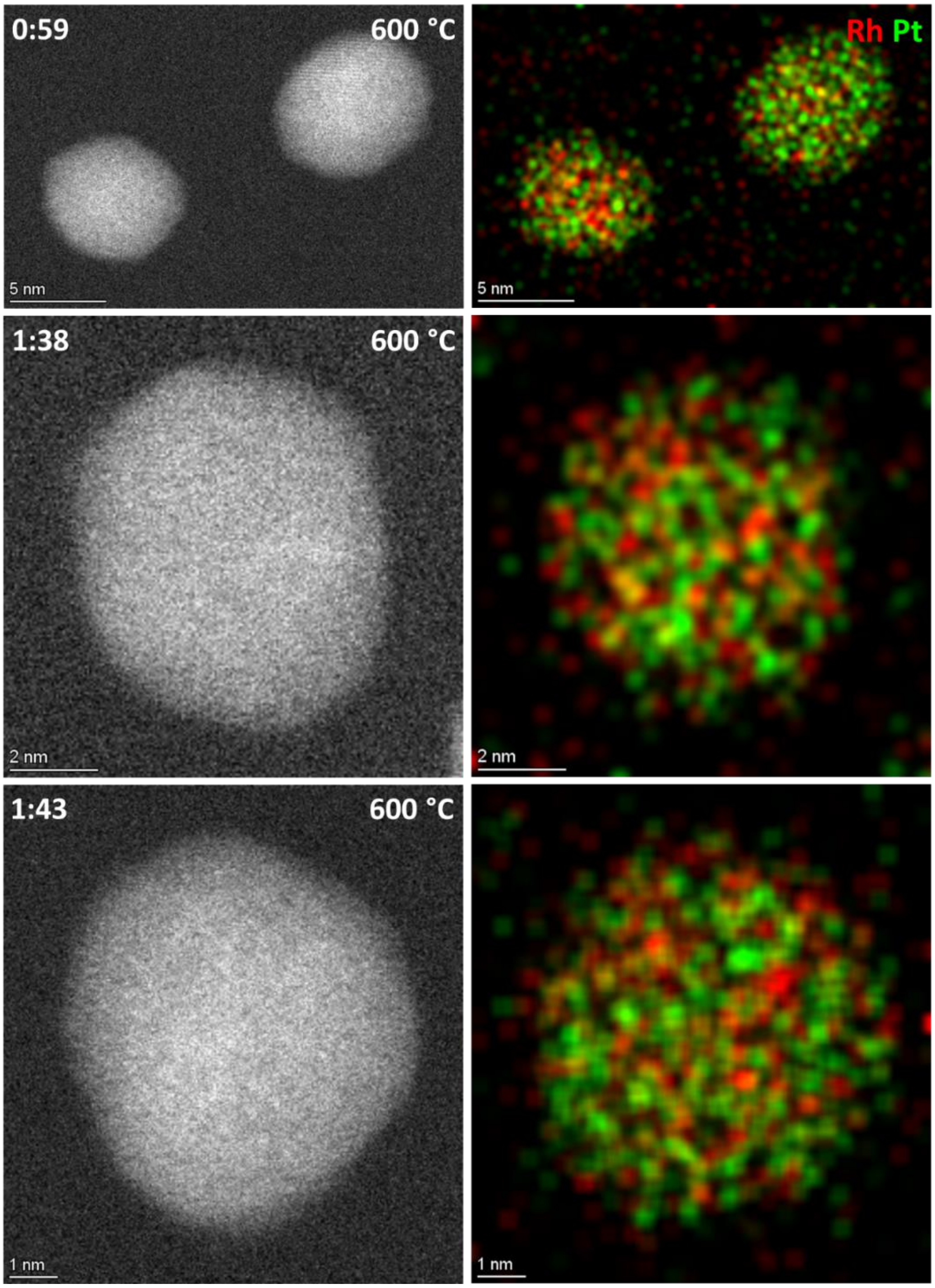
0:59
600 °C
5 nm
Rh Pt
5 nm
1:38
600 °C
2 nm
2 nm
1:43
600 °C
1 nm
1 nm

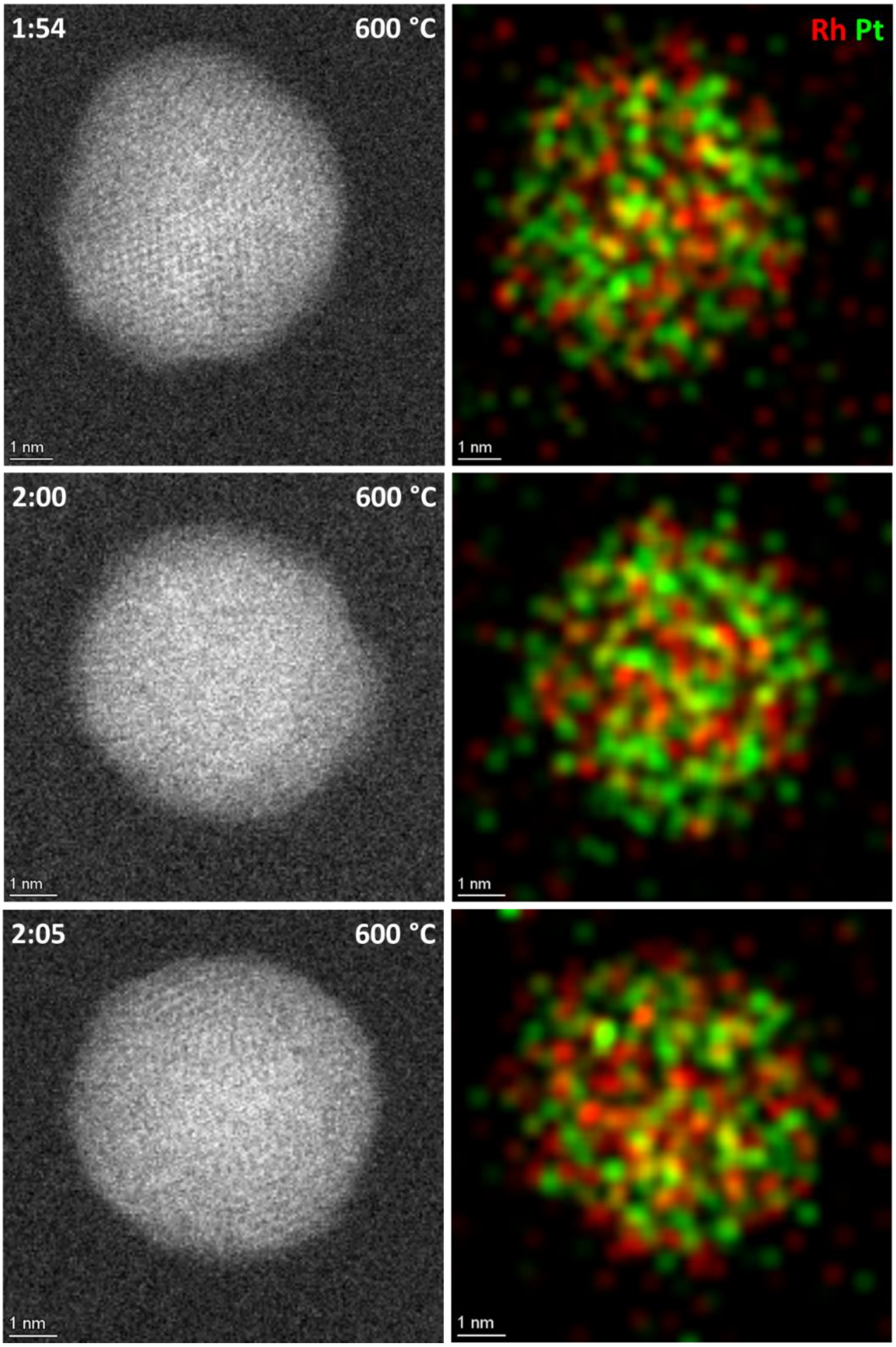
1:54
600 °C
1 nm
Rh Pt
1 nm
2:00
600 °C
1 nm
1 nm
2:05
600 °C
1 nm
1 nm

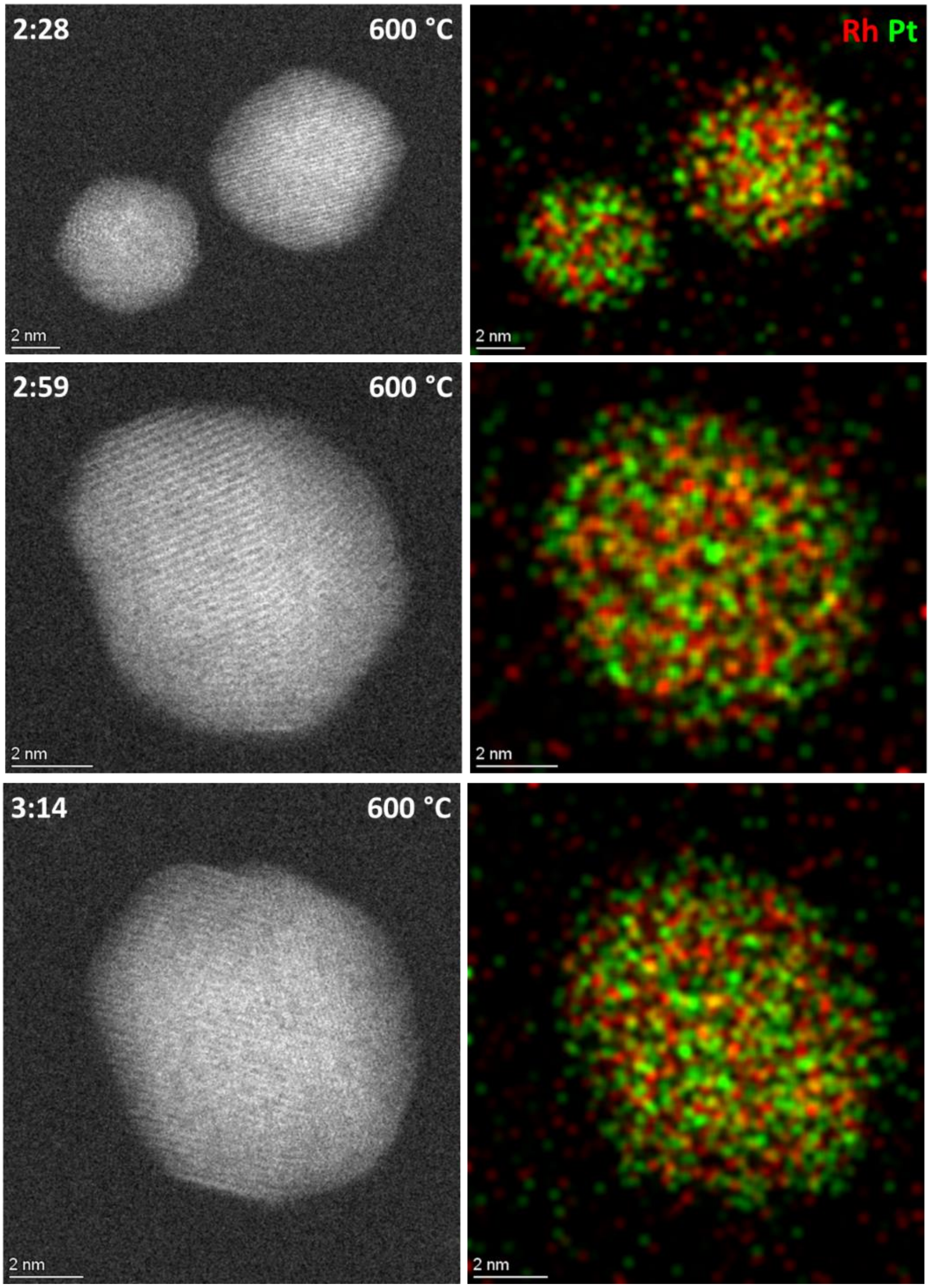
2:28
600 °C
Rh Pt
2 nm
2:59
600 °C
3:14
600 °C

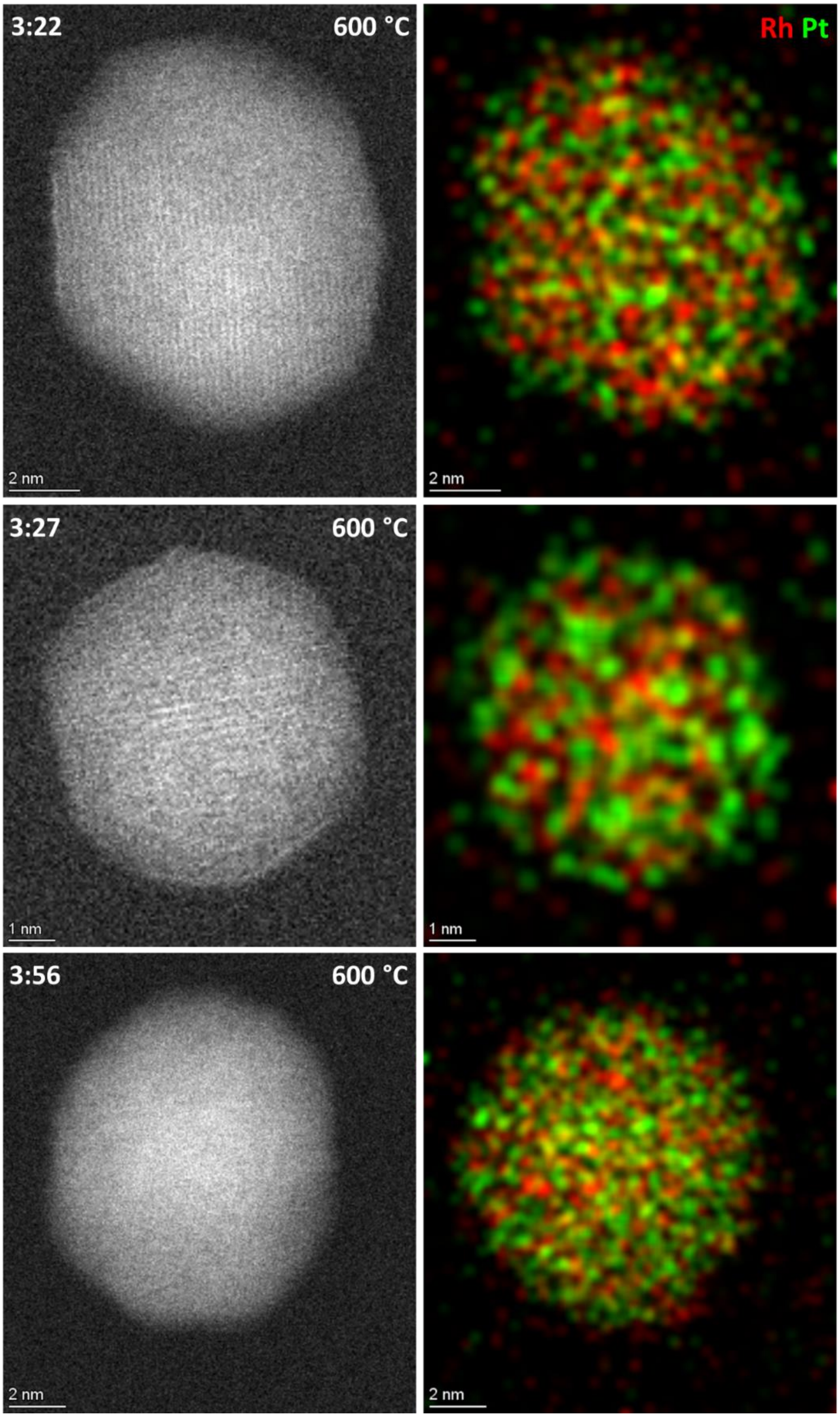
3:22
600 °C
Rh Pt
2 nm
2 nm
3:27
600 °C
1 nm
1 nm
3:56
600 °C
2 nm
2 nm

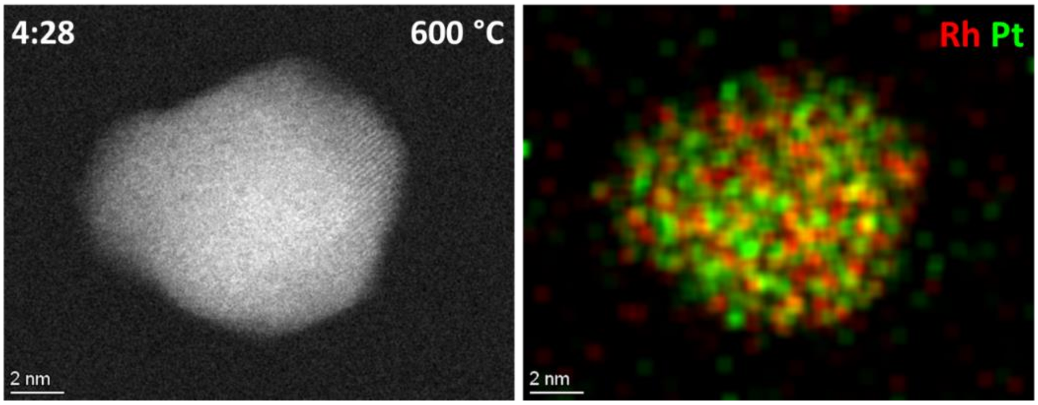

Fig. S12 **Pt-Rh solid solution nanoparticles imaged with HAADF-STEM and EDS elemental maps at 600 °C. The time after the temperature has reached 600 ˚C is indicated by h:min. Rh and Pt are mapped in red and green, respectively.**

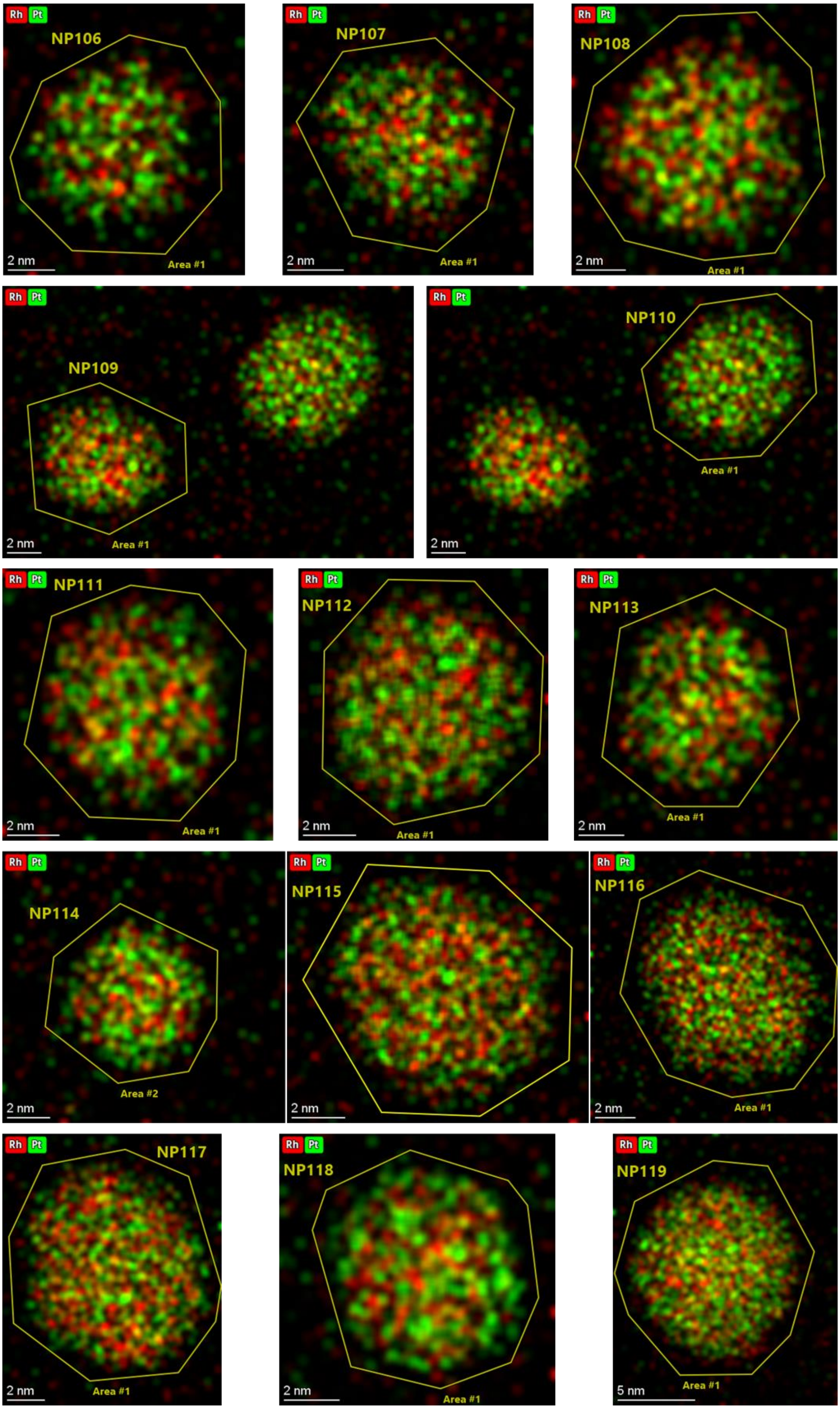

Fig. S13 **EDS elemental maps of Pt-Rh solid solution nanoparticles (NP106-119) at 600 °C showing the areas selected for quantitative EDS analysis (results in Table S4). Rh and Pt are mapped in red and green, respectively.**

Table S4 Results from quantitative EDS analysis of Pt-Rh solid solution nanoparticles (NP106-119) at 600 °C (see Fig. S13). The Pt-Rh molar composition is normalized to 100 at. %.

Nanoparticle	Time at 600 °C [h: mm]	Rh [at. %]	Pt [at. %]	Nanoparticle size [nm]
NP106	0:30	55 ±3.0	45 ±3.0	6.9
NP107	0:40	52 ±3.0	48 ±3.0	6.2
NP108	0:55	56 ±2.9	44 ±2.9	8.2
NP109	0:59	57 ±2.9	43 ±2.9	7.5
NP110	0:59	44 ±3.0	56 ±3.0	8.2
NP111	1:38	55 ±3.0	45 ±3.0	8.7
NP112	1:43	50 ±3.0	50 ±3.0	8.3
NP113	1:54	46 ±3.0	54 ±3.0	7.6
NP114	2:00	52 ±3.0	48 ±3.0	6.5
NP115	2:59	53 ±3.0	47 ±3.0	8.9
NP116	3:14	52 ±3.0	48 ±3.0	9.8
NP117	3:22	54 ±3.0	46 ±3.0	11.3
NP118	3:27	54 ±3.0	46 ±3.0	7.3
NP119	3:56	55 ±2.9	45 ±2.9	12.0

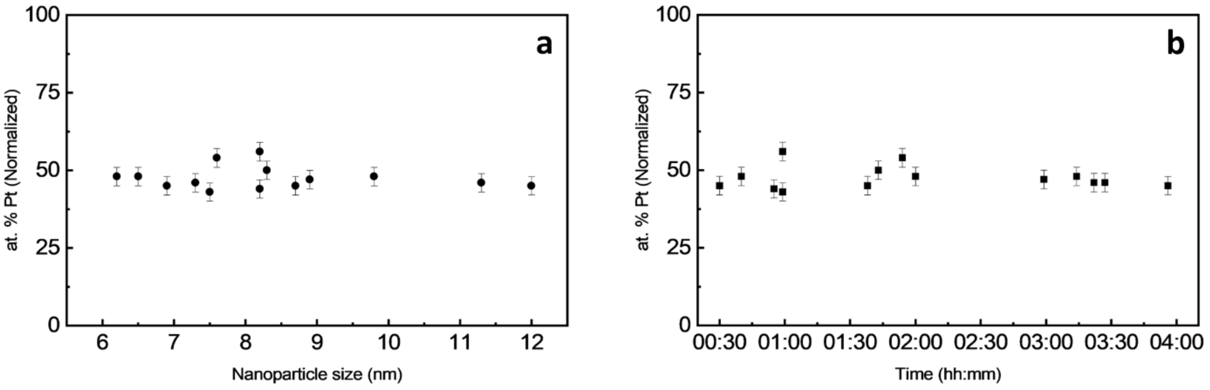

Fig. S14 Normalized Pt-content from quantitative EDS (Table S4) versus Pt-Rh solid solution nanoparticle size (a) and time (hh:mm) after reaching 600 °C (b). The Pt-Rh molar composition is normalized to 100 at. %.

2.3. Pt-Rh solid solution nanoparticles at 300 °C after cooling from 600 °C

An analysis of the Pt-Rh solid solution nanoparticles cooled from 600 °C to 300 °C over a period of 1.5 h in vacuum (see temperature program in Fig. S8) is described in this section. As in section 2.2, we examine the elemental distribution using HAADF-STEM images and corresponding EDS elemental maps while dwelling at 300 °C.

The HAADF-STEM images and corresponding EDS maps in Fig. S15 shows the 8 analyzed nanoparticles (NP120 – 127), with D_{avg} = 9.0 ± 1.2 nm and size range 8 – 11 nm, to be in the solid solution elemental distribution configuration. These results show that the sample has not undergone any changes with respect to elemental distribution upon cooldown (1.5 h) from 600 to 300 °C nor during the dwell time of 57 min at 300 °C.

The chemical composition of the individual Pt-Rh nanoparticles (NP120 – 127) was studied by quantitative EDS analysis versus time after reaching 300 °C. The areas selected for analysis are shown in Fig. S16 and the results are summarized in Table S5. The average composition of the nanoparticles (±1σ) normalized to Pt and Rh was 46.1 ± 3.5 at. % Pt. The Pt content of the individual nanoparticles is plotted versus nanoparticle size (Fig. S17a) and time progressed at 300 °C (Fig. 17b) to look for possible correlations between Pt content and nanoparticle size/dwell time. No correlations were identified.

Overall, the HAADF-STEM and EDS elemental maps show the Pt-Rh solid solution nanoparticles (D_{avg} = 9.0 ± 1.2 nm, with size range 8 – 11 nm) to remain unchanged when cooled down from 600 °C to 300 °C and during the dwell time at 300 °C (57 min).

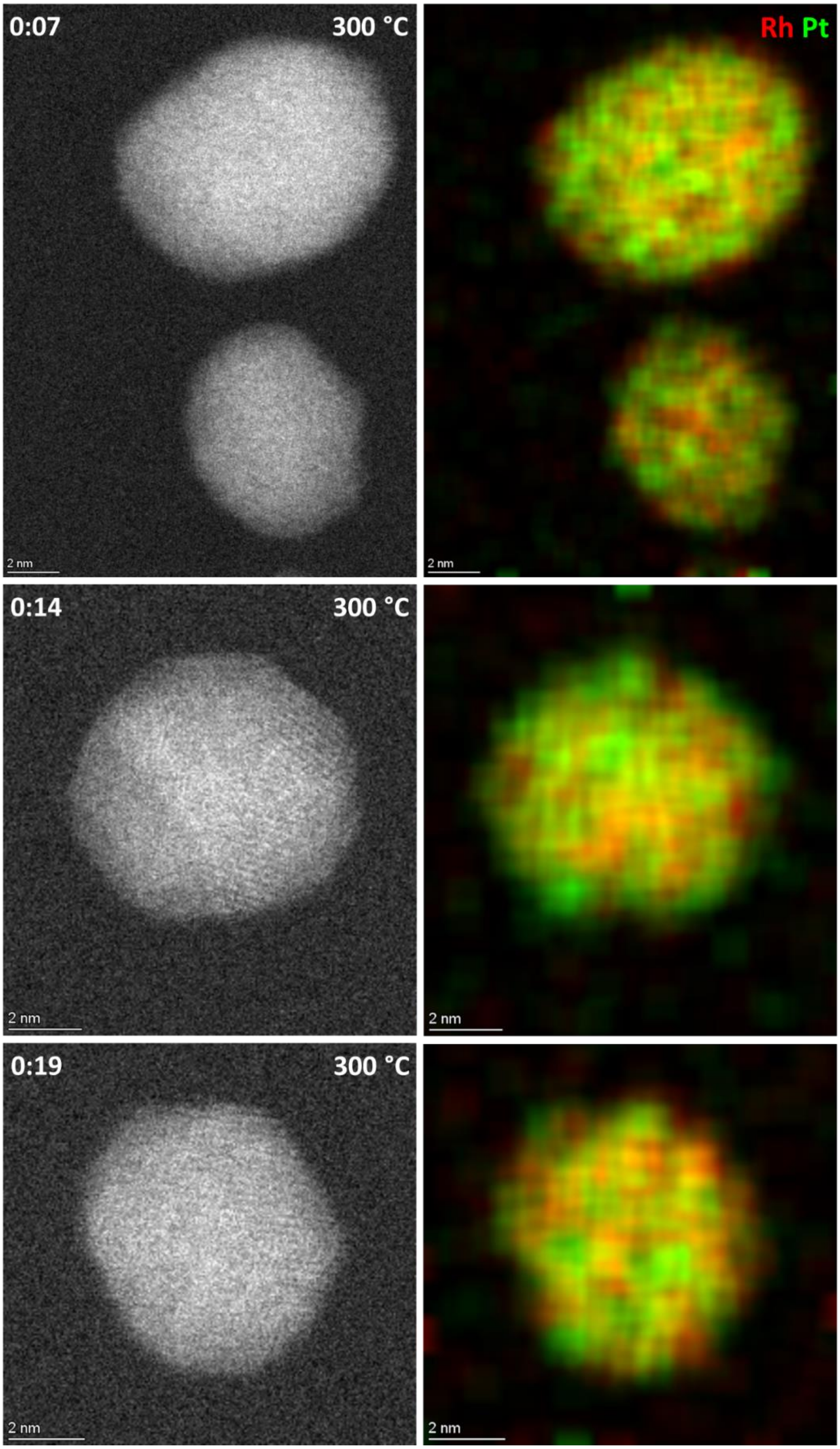
0:07
300 °C
Rh Pt
2 nm
2 nm
0:14
300 °C
2 nm
2 nm
0:19
300 °C
2 nm
2 nm

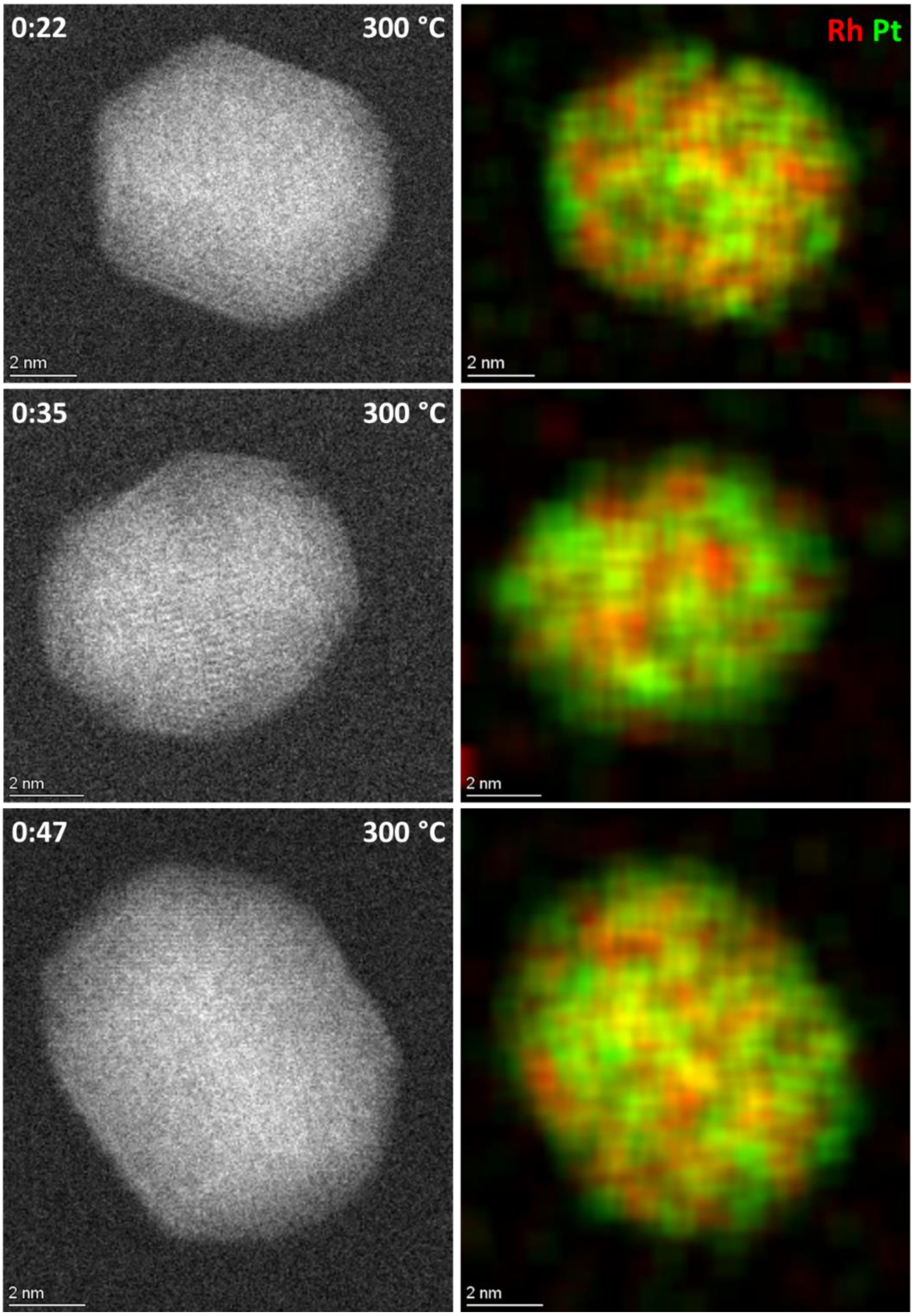
0:22
300 °C
Rh Pt
2 nm
2 nm
0:35
300 °C
2 nm
2 nm
0:47
300 °C
2 nm
2 nm

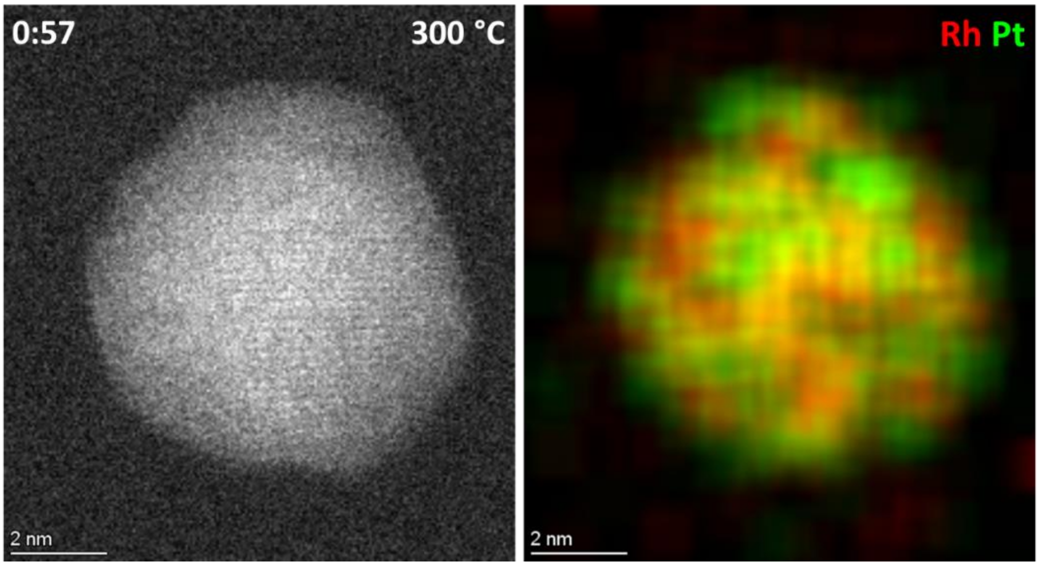

Fig. S15 **Pt-Rh solid solution nanoparticles imaged with HAADF-STEM and EDS elemental maps at 300 °C after cooldown from 600 °C. The time after the temperature has reached the targeted temperature is indicated by h:min. Rh and Pt are mapped in red and green, respectively.**

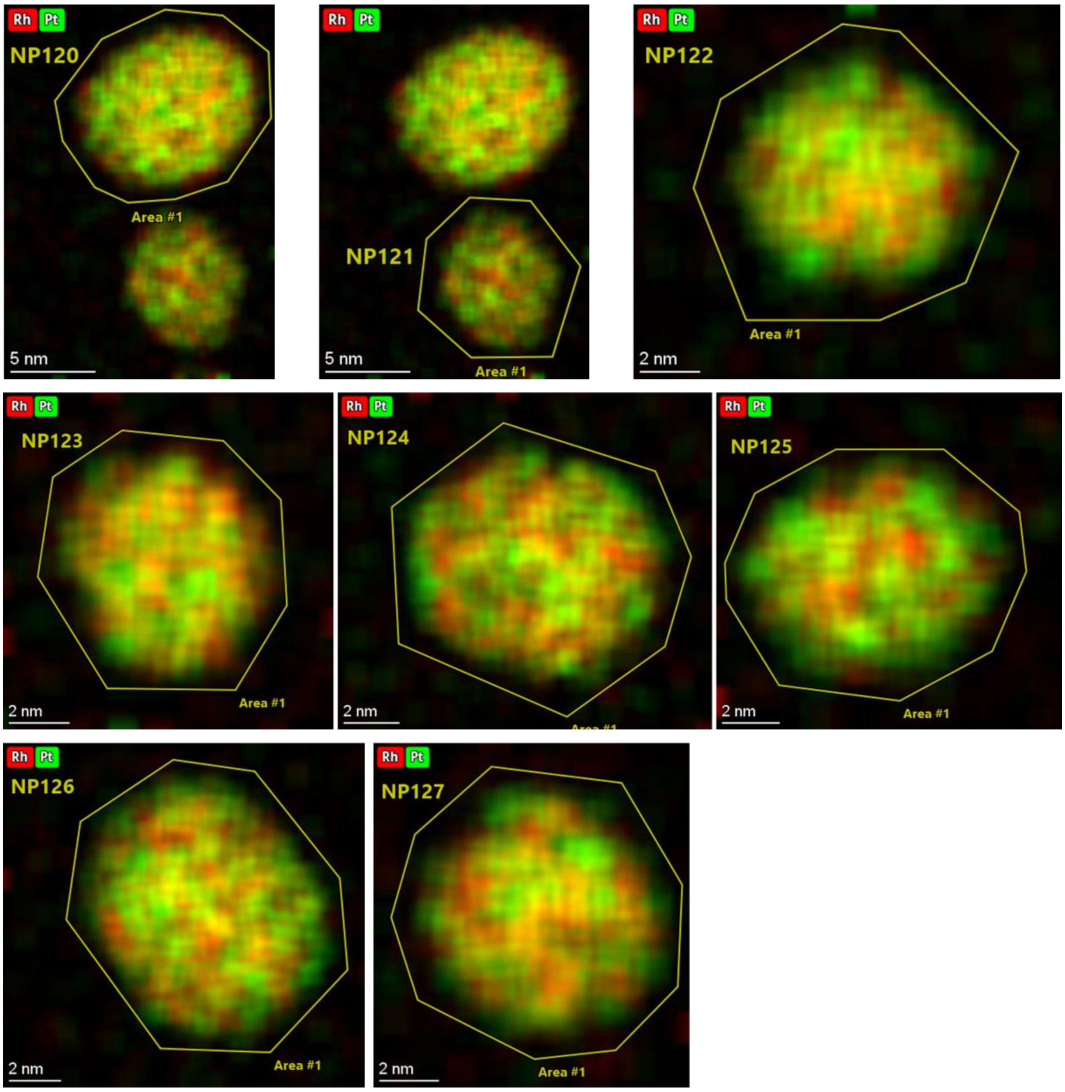

Fig. S16 EDS elemental maps of Pt-Rh solid solution nanoparticles (NP120-127) at 300 °C after cooldown from 600 °C showing the areas selected for quantitative EDS analysis (results in Table S5). Rh and Pt are mapped in red and green, respectively.

Table S5 Quantitative EDS analysis of Pt-Rh solid solution nanoparticles (NP120-127) at 300 °C after cooldown from 600 °C (see Fig. S16). The Pt-Rh molar composition is normalized to 100 at. %.

Nanoparticle	Time at 300 °C [h: mm]	Rh [at. %]	Pt [at. %]	Nanoparticle size [nm]
NP120	0:07	54 ±2.9	46 ±2.9	10.8
NP121	0:07	55 ±2.9	45 ±2.9	8.3
NP122	0:14	54 ±3.0	46 ±3.0	7.9
NP123	0:19	50 ±3.0	50 ±3.0	7.5
NP124	0:22	58 ±2.9	42 ±2.9	9.6
NP125	0:35	52 ±3.0	48 ±3.0	8.8
NP126	0:47	59 ±2.9	41 ±2.9	10.5
NP127	0:57	49 ±3.0	51 ±3.0	8.6

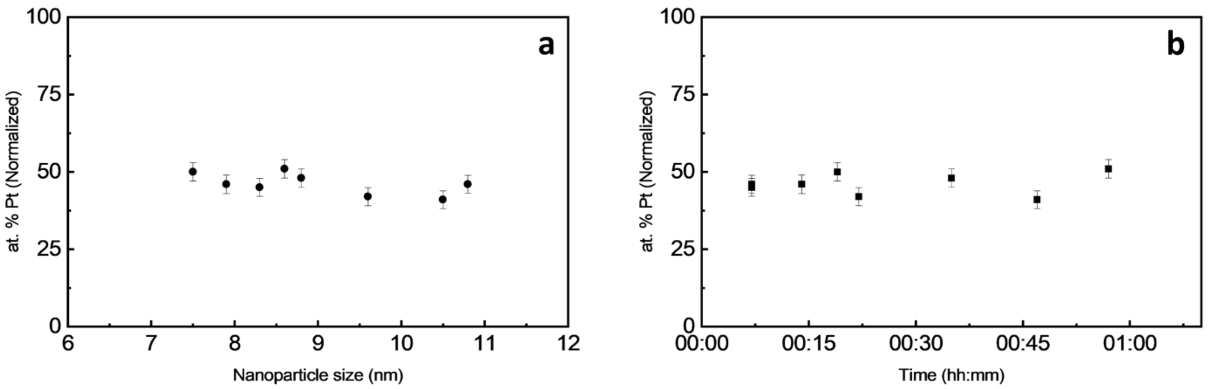

Fig. S17 Normalized Pt-content of individual nanoparticles from quantitative EDS (Table S5) versus Pt-Rh solid solution nanoparticle size (a) and time (hh:mm) progressed (b) at 300 °C after cooldown from 600 °C. The Pt-Rh molar composition is normalized to 100 at. %.

2.4. Pt-Rh solid solution nanoparticles at room temperature after cooling from 300 °C

An analysis of the Pt-Rh solid solution nanoparticles first heated to 600 °C, followed by cooling (during 1.5 h) to 300 °C and dwelling for 57 min before finally cooling (during 1.5 h) to room temperature in vacuum is presented here (see temperature program in Fig. S8). In this section, we examine the element distribution by HAADF-STEM images and EDS elemental maps versus time after reaching room temperature.

The HAADF-STEM images and EDS maps in Fig. S18 show the 19 analyzed nanoparticles (D_{avg} = 9.3 ± 2.4 nm, size range 5 – 15 nm) to be in the solid solution elemental distribution configuration. These results document that the sample has not undergone any change with respect to the element distribution upon cooldown (1.5 h) from 300 °C to room temperature nor during the time after room temperature was reached (2 h 39 min).

The chemical composition of a selection (NP128 – 141) of individual Pt-Rh nanoparticles was studied by quantitative EDS analysis as time progressed after reaching room temperature. The areas selected for analysis are shown in Fig. S19 and the results are summarized in Table S6. The average composition of the nanoparticles (± 1σ) normalized to Pt and Rh was 48.1 ± 5.1 at. % Pt. The Pt content of the individual nanoparticles was plotted versus nanoparticle size (Fig. S20a) to determine possible correlations between the two parameters. As seen in the sample at room temperature before heating (section 2.1), slightly higher Pt-content is observed in the smallest nanoparticles studied (5.3 nm), but this is expected due to the difference in reduction rates of the Pt- and Rh-precursors in the synthesis of the solid solution nanoparticles (see reference[1]). Next, the Pt content of the individual nanoparticles was plotted versus time at room temperature after cooldown from 300 °C (Fig. S20b) to look for correlations. No correlations were identified between these two parameters.

Overall, the HAADF-STEM and EDS elemental maps show the Pt-Rh solid solution nanoparticles (D_{avg} = 9.3 ± 2.4 nm, size range 5 – 15 nm) to remain unchanged when cooled from 300 °C to room temperature and during the time of characterization at room temperature (2 h 39 min).

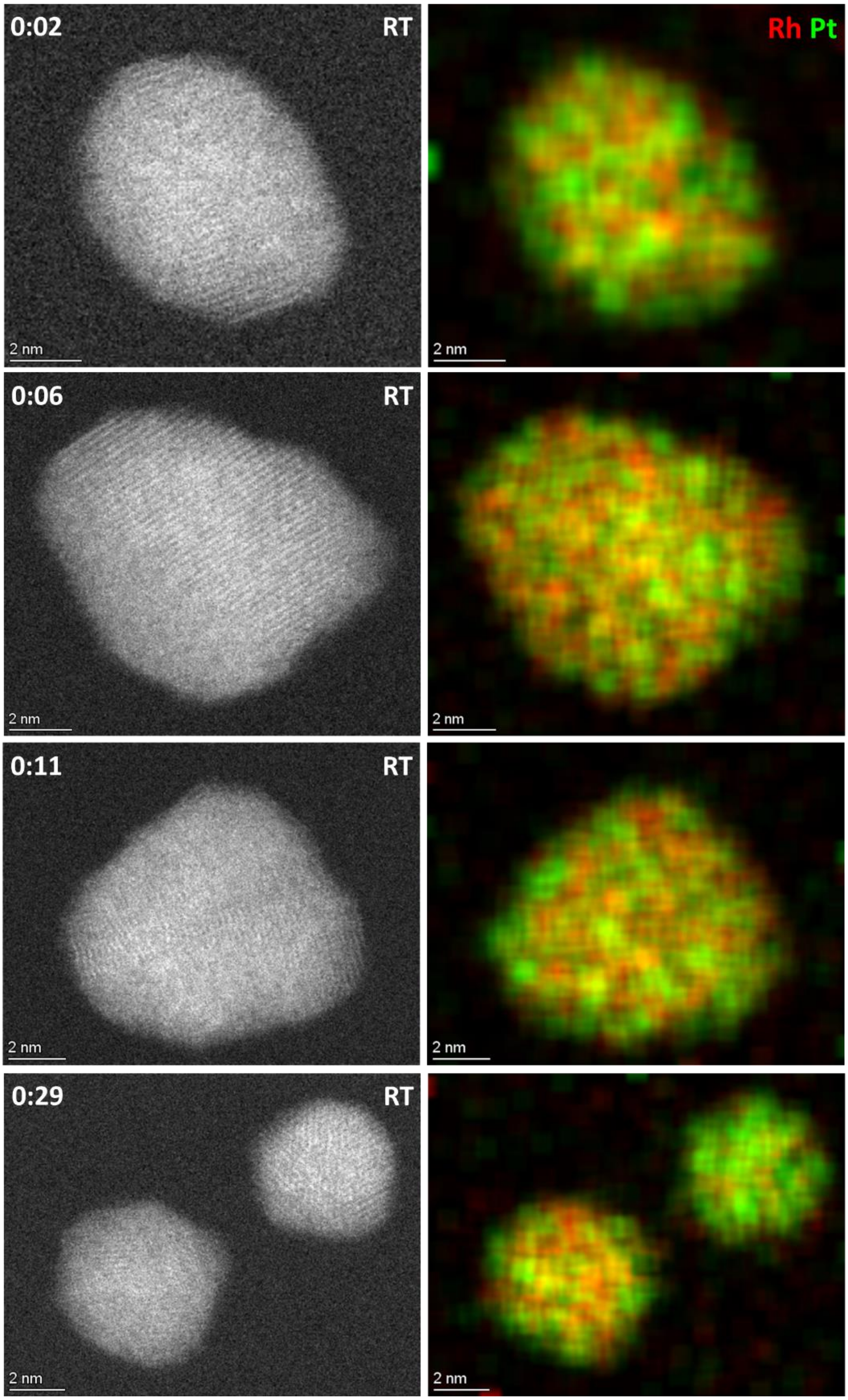
0:02
RT
Rh Pt
2 nm
2 nm
0:06
RT
2 nm
2 nm
0:11
RT
2 nm
2 nm
0:29
RT
2 nm
2 nm

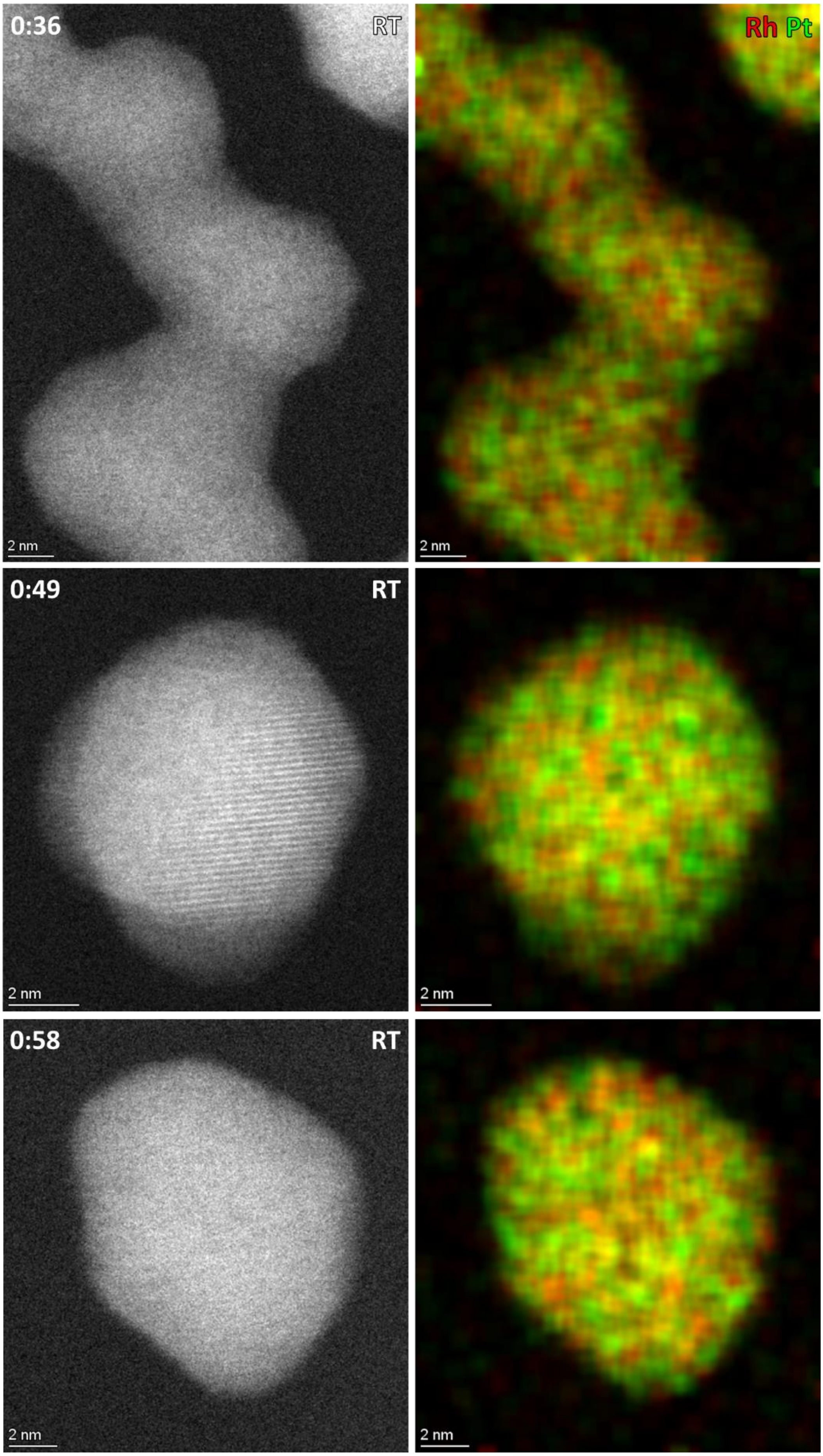
0:36
RT
Rh Pt
2 nm
2 nm
0:49
RT
2 nm
2 nm
0:58
RT
2 nm
2 nm

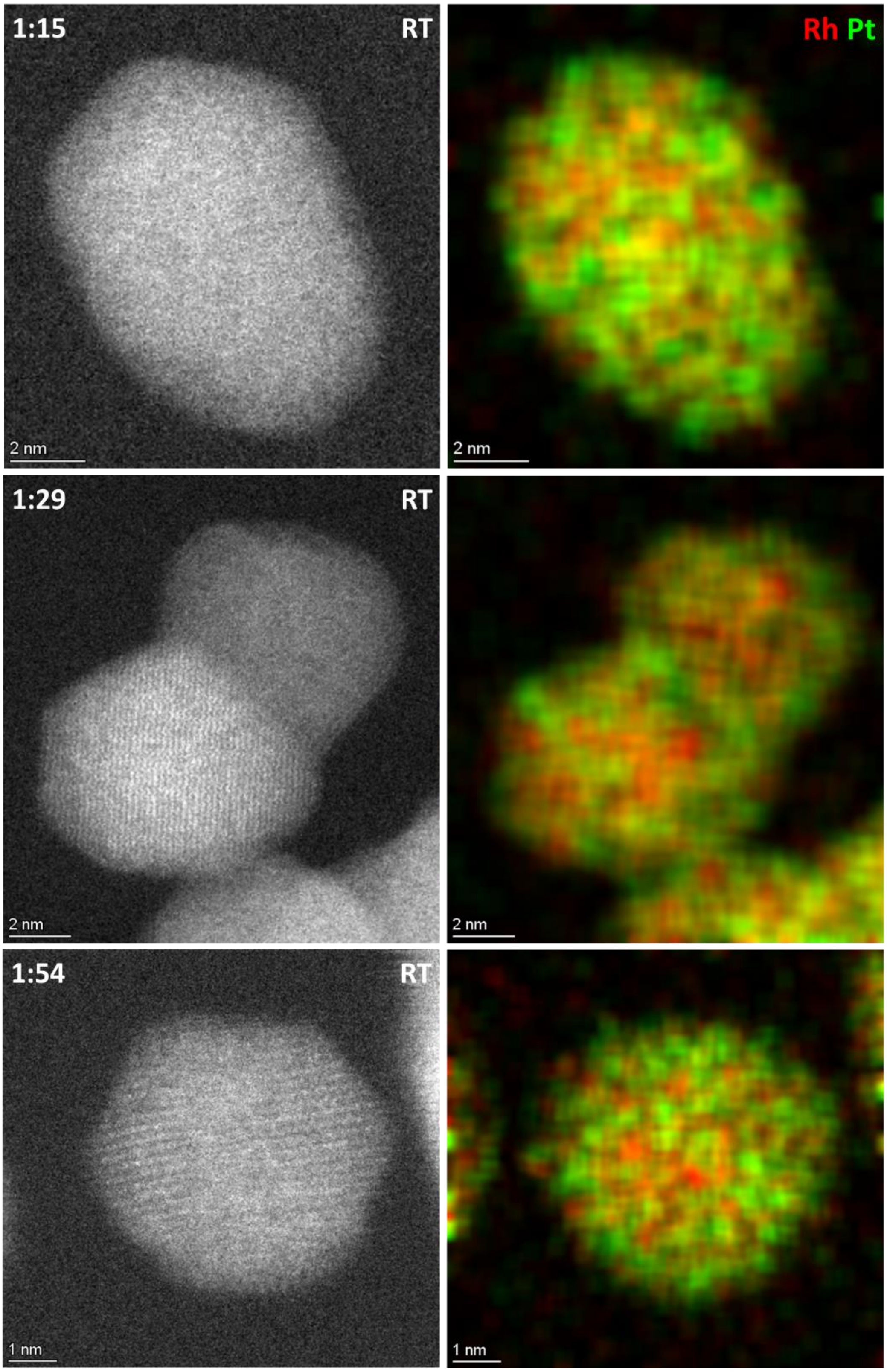
1:15
RT
2 nm
Rh Pt
2 nm
1:29
RT
2 nm
2 nm
1:54
RT
1 nm
1 nm

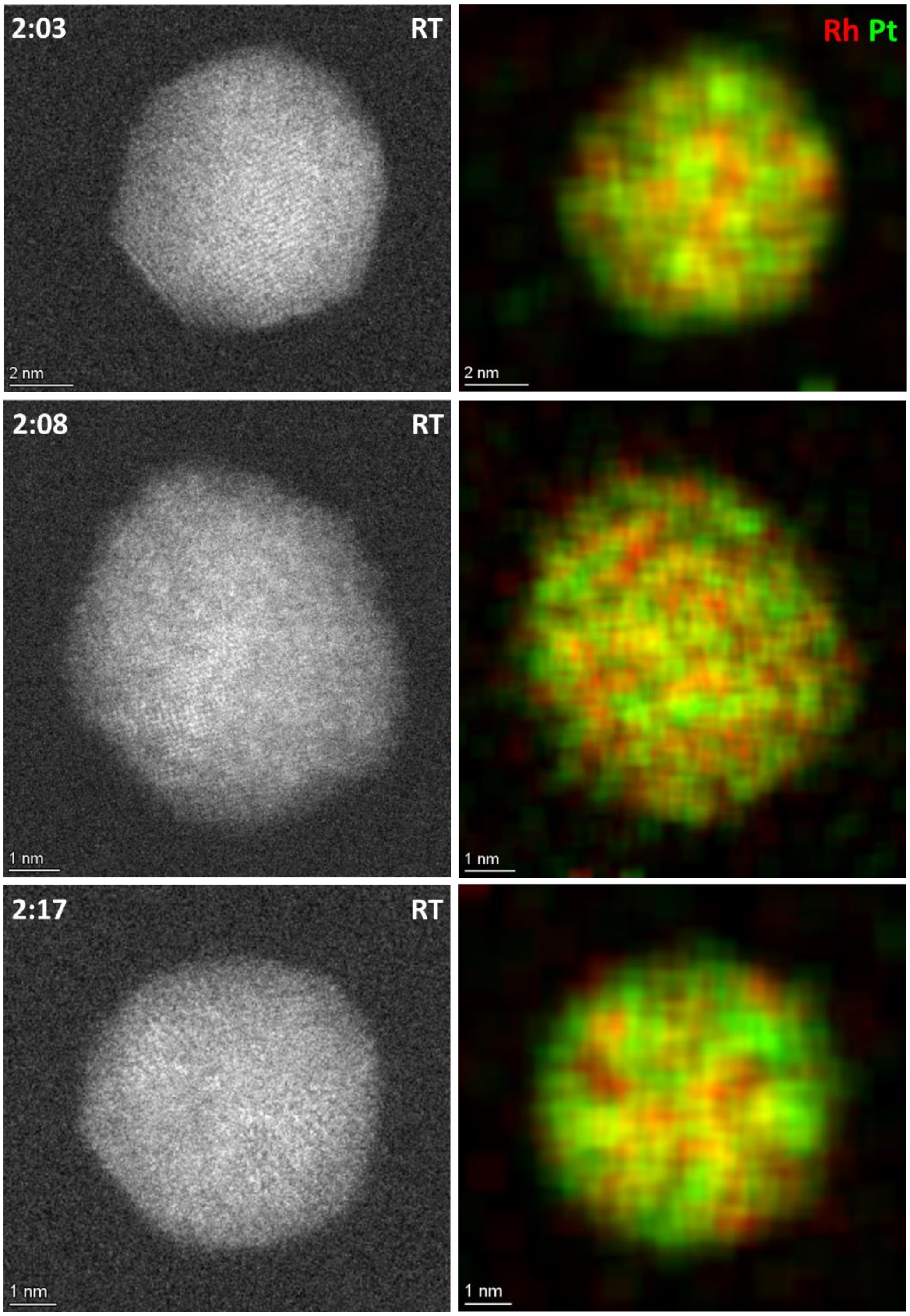
2:03
RT
2 nm
Rh Pt
2 nm
2:08
RT
1 nm
1 nm
2:17
RT
1 nm
1 nm

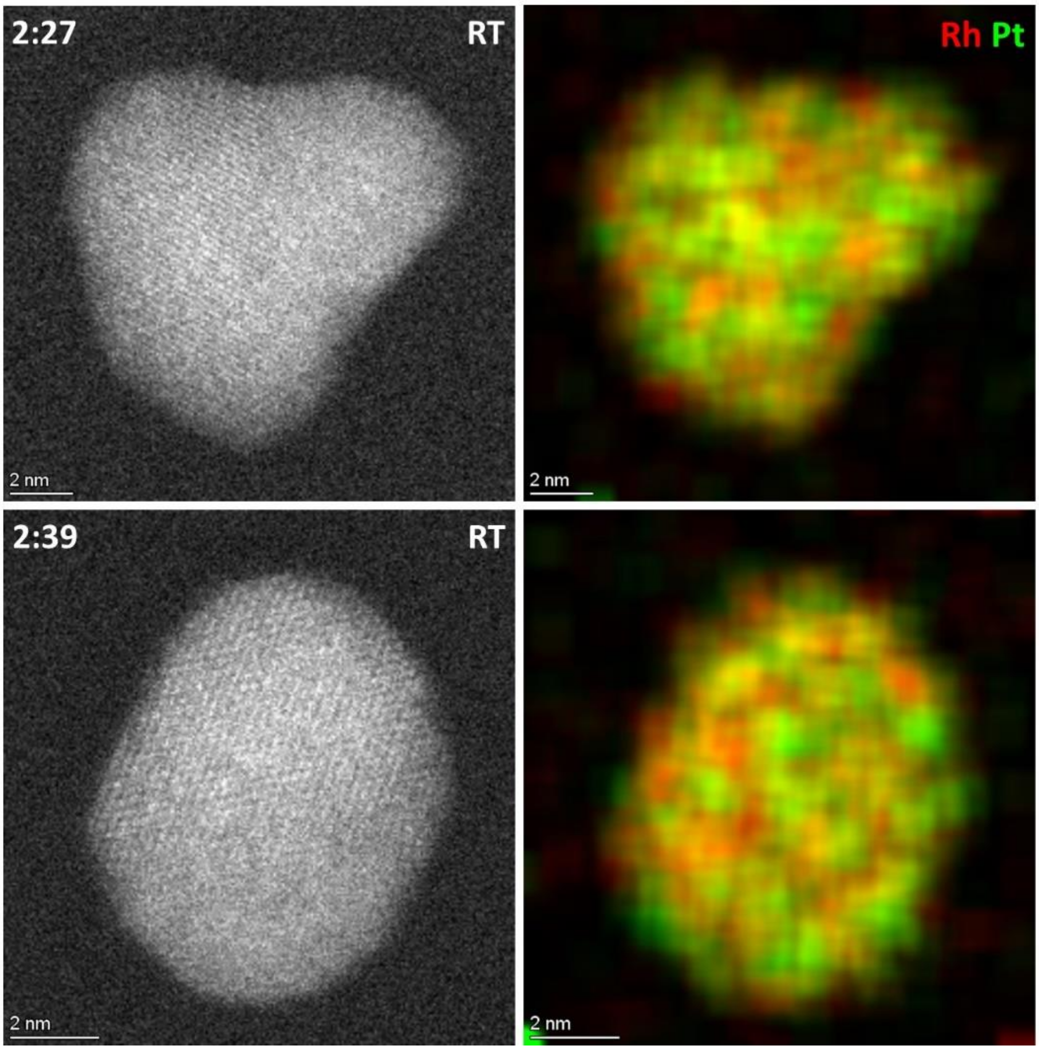

Fig. S18 **Pt-Rh solid solution nanoparticles imaged with HAADF-STEM and EDS elemental maps at room temperature after cooldown from 300 °C. The time after the temperature has reached room temperature is indicated by h:min. Rh and Pt are mapped in red and green, respectively.**

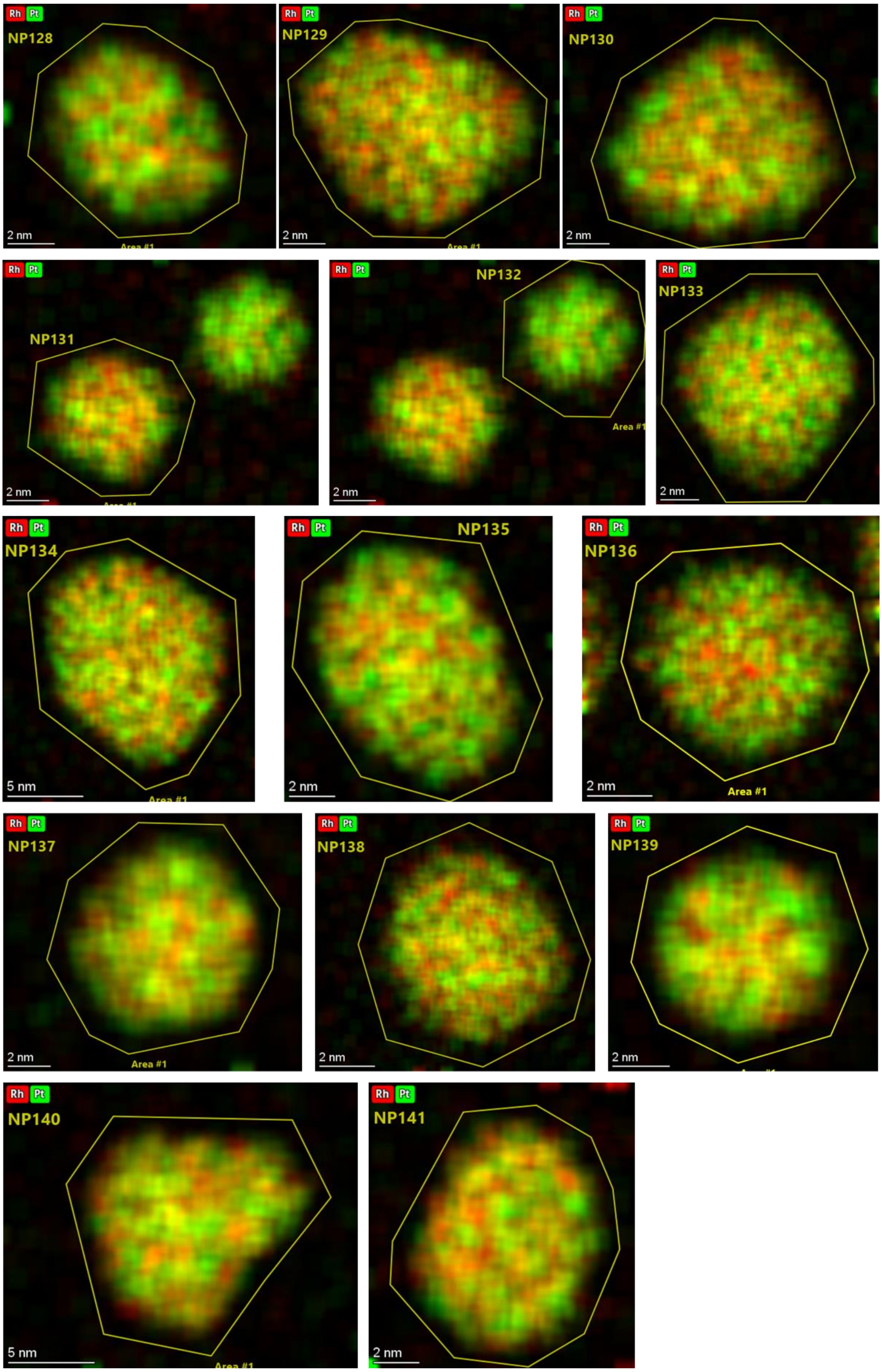

Fig. S19 **EDS elemental maps of Pt-Rh solid solution nanoparticles (NP128-141) at room temperature after cooldown from 300 °C showing the areas selected for quantitative EDS analysis (results in Table S6). Rh and Pt are mapped in red and green, respectively.**

Table S6 Results from quantitative EDS analysis of Pt-Rh solid solution nanoparticles (NP128-141) at room temperature after cooldown from 300 °C (see Fig. S19). The Pt-Rh molar composition is normalized to 100 at. %.

Nanoparticle	Time at RT [h: mm]	Rh [at. %]	Pt [at. %]	Nanoparticle size [nm]
NP128	0:02	51 ±3.0	49 ±3.0	8.4
NP129	0:06	54 ±2.9	46 ±2.9	11.5
NP130	0:11	53 ±2.9	47 ±2.9	10.6
NP131	0:29	53 ±3.0	47 ±3.0	6.6
NP132	0:29	36 ±2.9	64 ±3.0	5.3
NP133	0:49	51 ±3.0	49 ±3.0	10.5
NP134	0:58	52 ±3.0	48 ±3.0	14.5
NP135	1:15	56 ±2.9	44 ±2.9	11.2
NP136	1:54	50 ±3.0	50 ±3.0	6.4
NP137	2:03	56 ±2.9	44 ±2.9	9.3
NP138	2:08	56 ±2.9	44 ±2.9	7.4
NP139	2:17	52 ±3.0	48 ±3.0	6.5
NP140	2:27	56 ±2.9	44 ±2.9	13.4
NP141	2:39	50 ±3.0	50 ±3.0	9.8

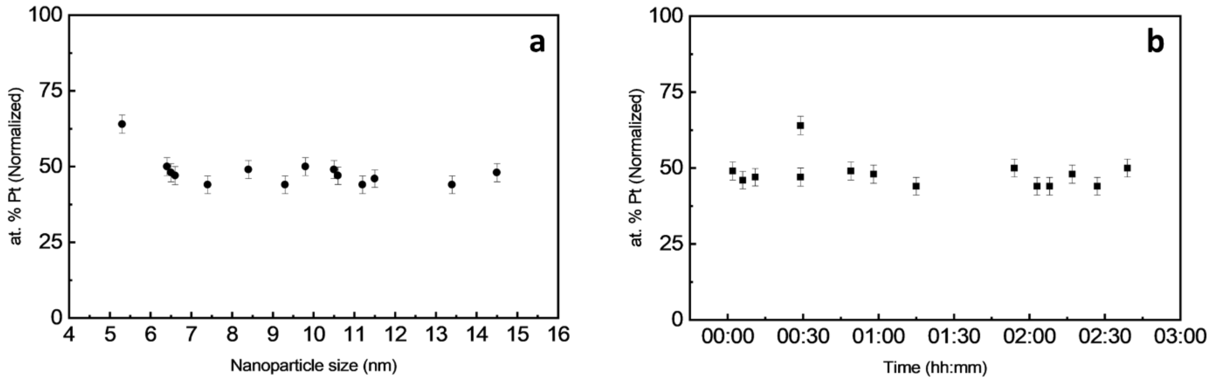

Fig. S20 Normalized Pt-content from quantitative EDS results (Table S6) versus Pt-Rh solid solution nanoparticle size (a) and time (hh:mm) progressed at room temperature after cooldown from 300 °C. The Pt-Rh molar composition is normalized to 100 at. %.

3. In-situ Transmission Electron Microscopy study of larger Rh(core)-Pt(shell) nanoparticles heated to 600 °C

As we observed segregation to occur in some of the larger (≳ 13 nm) as-synthesized Rh(core)-Pt(shell) nanoparticles (batch 1) after heating to 650 °C (see Fig. 4 in the manuscript), we decided to investigate whether slightly larger Rh(core)-Pt(shell) nanoparticles segregate when exposed to the same heat treatment as employed for the solid solution nanoparticles (section 2). We synthesized another batch (batch 2) of Rh(core)-Pt(shell) nanoparticles and exposed them to the heating program (also included in the main manuscript, Fig. 7) shown in Fig. S21. Only the slightly larger size fraction (batch 2) relative to batch 1 of Rh(core)-Pt(shell) nanoparticles was investigated. We also revisited the sample after keeping it at room temperature in vacuum for 11 days. The average size of all nanoparticles from batch 2 analyzed in the temperature range 600 °C – room temperature (after 11 days) was 15.0 ± 3.0 nm (size range 9 – 24 nm).

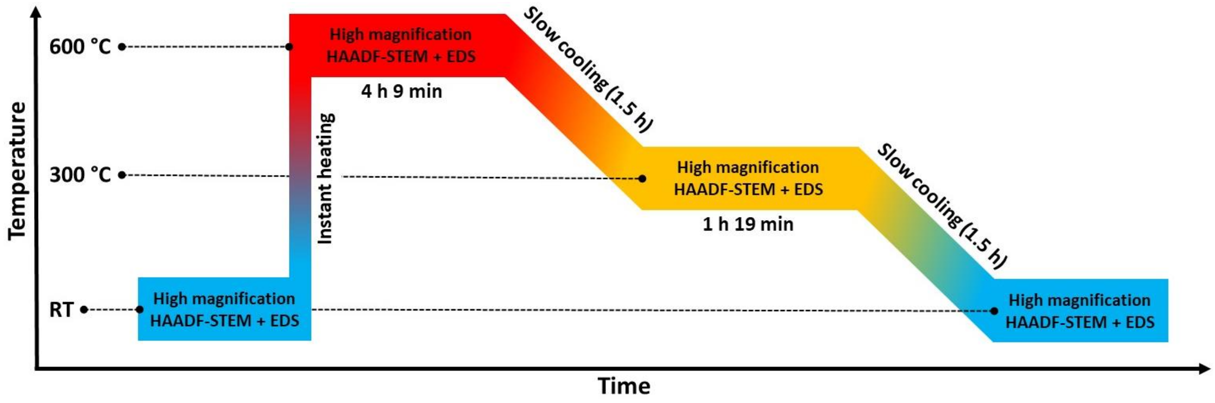

Fig. S21 Temperature program used in the in-situ TEM heating experiment of the larger Rh(core)-Pt(shell) nanoparticles heated to 600 °C and stepwise cooled to room temperature in a controlled manner.

3.1. Larger Rh(core)-Pt(shell) nanoparticles at room temperature before heating

For the sake of documentation, we characterized the nanoparticles at room temperature before heating to verify that the batch was homogeneous (Fig. S21).

From the HAADF-STEM images and EDS elemental maps in Fig. S22 we conclude that the nanoparticles in batch 2 of Rh(core)-Pt(shell) nanoparticles are well-defined in terms of element distribution.

The chemical composition of individual Rh(core)-Pt(shell) nanoparticles (NP1 – 5) was determined by quantitative EDS analysis. The average composition of the nanoparticles (± 1σ) normalized to Pt and Rh was 62.4 ± 11.2 at. % Pt. The areas selected for analysis are shown in Fig. S23 and the results are summarized in Table S7. The Pt content of the individual nanoparticles was plotted versus nanoparticle size (Fig. S24). Ignoring the nanoparticle with D = 9.5 nm, because this might be an outlier, no correlation between nanoparticle size and Pt content was found.

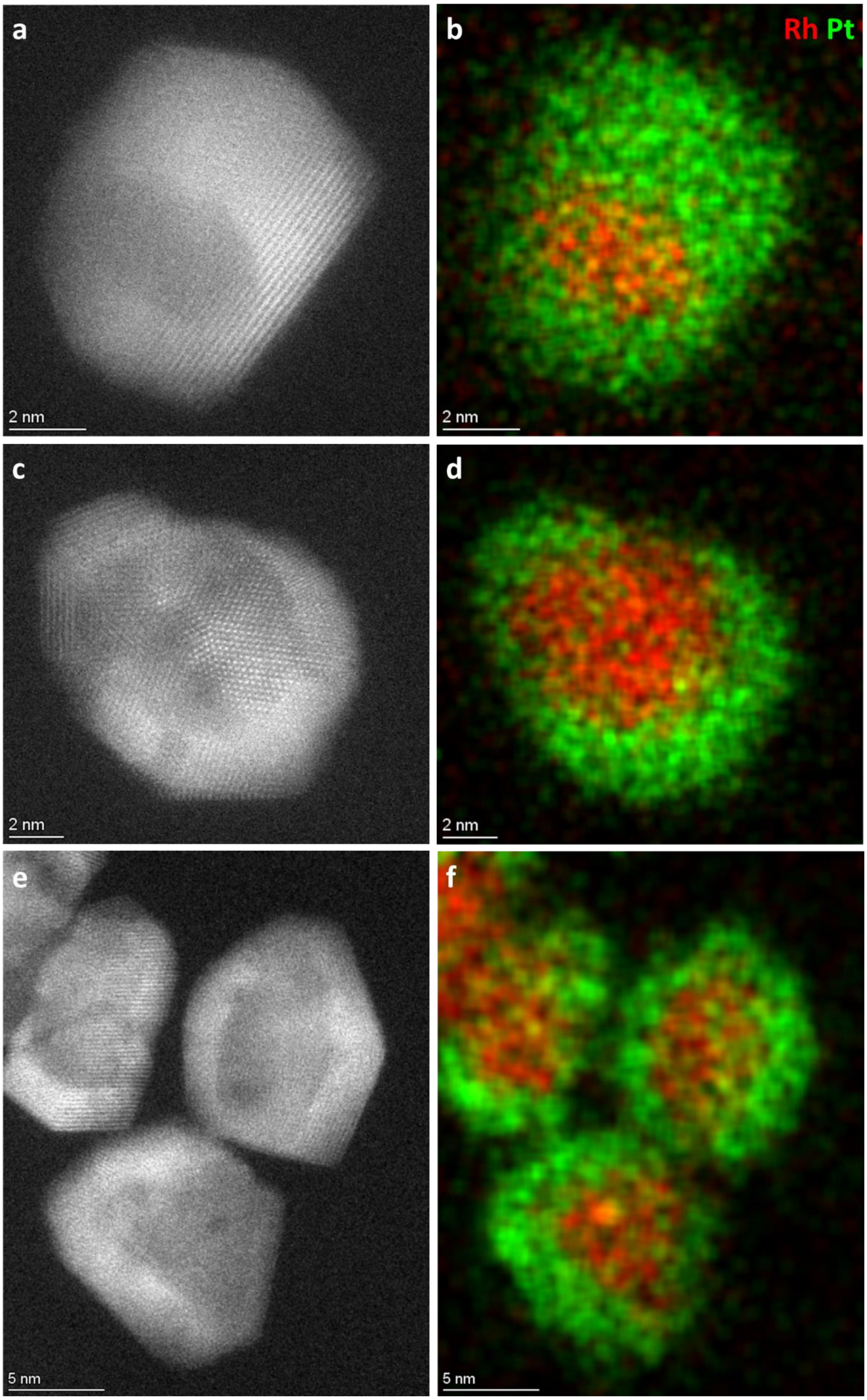
a
b
Rh Pt
2 nm
2 nm
c
d
2 nm
2 nm
e
f
5 nm
5 nm

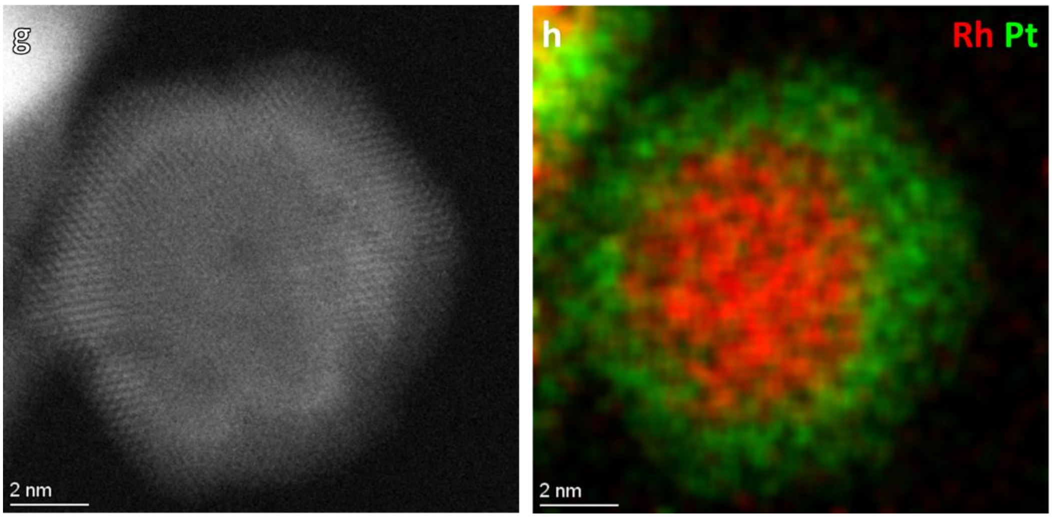

Fig. S22 HAADF-STEM images and EDS elemental maps of Rh(core)-Pt(shell) nanoparticles at room temperature before heating to 600 °C. Rh and Pt are mapped in red and green, respectively.

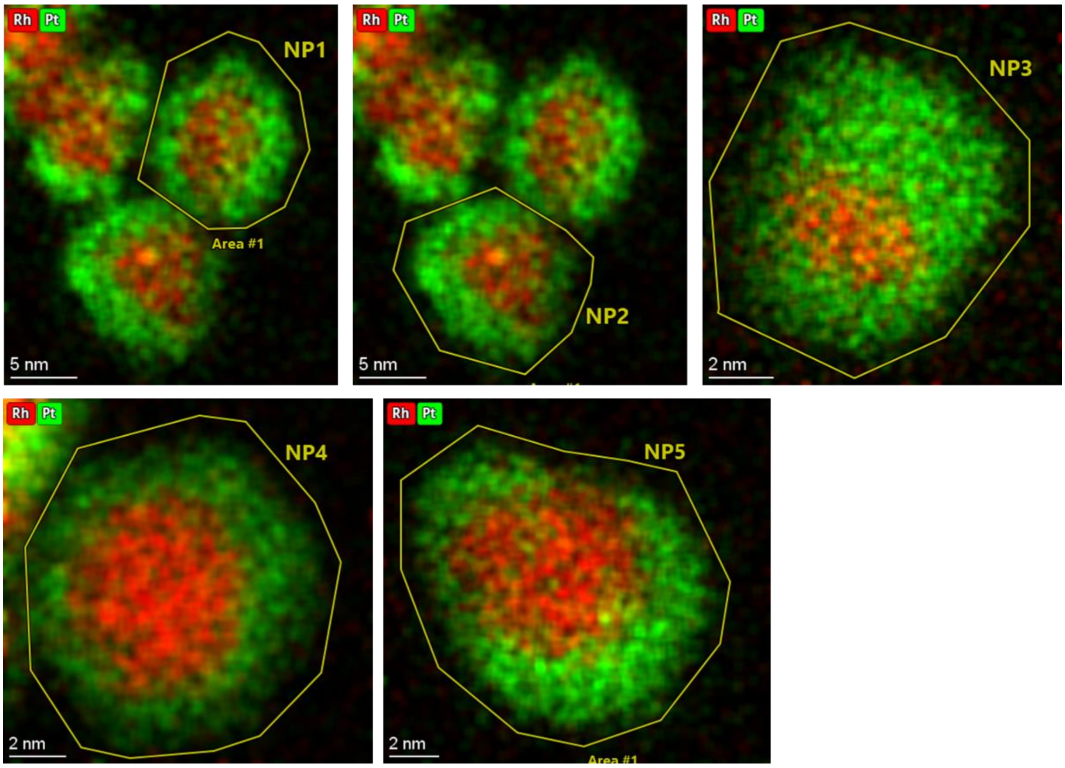

Fig. S23 EDS elemental maps of Rh(core)-Pt(shell) nanoparticles (NP1-5) at room temperature before heating to 600 °C showing the areas selected for quantitative EDS analysis (results in Table S7). Rh and Pt are mapped in red and green, respectively.

Table S7 Results from quantitative EDS analysis of Rh(core)-Pt(shell) nanoparticles (NP1-5) at room temperature before heating to 600 °C (see Fig. S23). The Pt-Rh molar composition is normalized to 100 at. %.

Nanoparticle	Rh [at. %]	Pt [at. %]	Nanoparticle size [nm]
NP1	40 ±2.8	60 ±2.8	13.4
NP2	42 ±2.9	58 ±2.9	13.7
NP3	18 ±1.7	82± 1.7	9.5
NP4	42 ±2.9	58 ±2.9	12.0
NP5	46 ±2.9	54 ±2.9	13.8

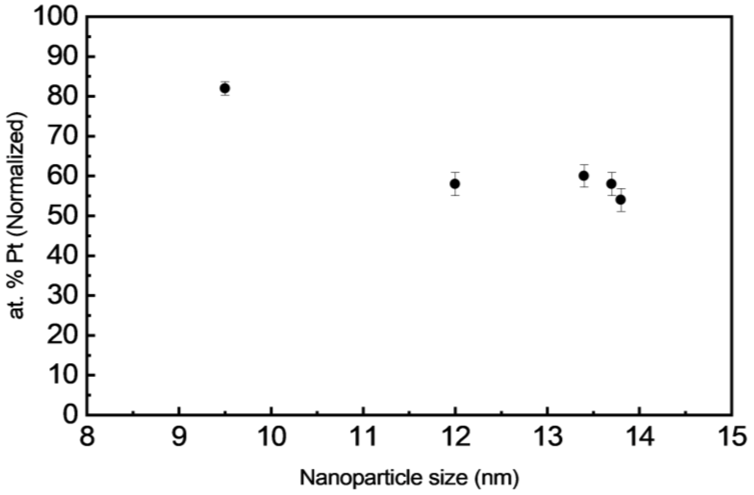

Fig. S24 Normalized Pt-content from quantitative EDS results (Table S7) versus Rh(core)-Pt(shell) nanoparticle size at room temperature before heating to 600 °C. The Pt-Rh molar composition is normalized to 100 at. %.

3.2. Larger Rh(core)-Pt(shell) nanoparticles heated to 600 °C

The Rh(core)Pt-(shell) nanoparticles (batch 2) described in section 3.1 were heated to 600 °C in vacuum (see temperature program in Fig. S21). In this section, we examine the element distribution by HAADF-STEM images and corresponding EDS elemental maps versus dwell time at 600 °C.

In total, 14 nanoparticles (NP6 – 19), with D_{avg} = 14.6 ± 2.7 nm were analyzed. In Fig. S25, the HAADF-STEM image of a Rh(core)-Pt(shell) nanoparticle exposed to 600°C in vacuum at 0:35 is shown, and we observe two darker cores surrounded and separated by a brighter shell. The EDS map verifies this to correspond to two Rh-rich cores and a Pt-rich shell. No such configurations with twin cores were observed in the sample at room temperature. Further, the Rh has diffused into the Pt-rich shell and vice versa to a larger extent than in the sample at room temperature. This proves that already after only 35 min, some mixing of Pt and Rh has occurred. We classify the particle as segregated. After the 1:07 time-point, the HAADF-STEM image and the EDS map still show resemblance to the core-shell configuration (segregated) at room temperature, however the Rh-rich core here is less well-defined, and Rh and Pt have diffused

into the shell and core, respectively. At the 1:23 time-point, we clearly see that the element distribution is no longer in the core-shell configuration from the nanoparticle imaged. Only a small Rh-rich patch within the nanoparticle remains, and thus we classify the particle as partly segregated. In the particle at 1:37, we still see a part of the particle richer in Rh (partly segregated). The particles imaged at 1:51 and 2:13 show patches of Rh-and Pt rich areas (partly segregated). At time-point 2:26, the particle on the far left is overlapping with other particles and is thus hard to interpret. The particle in the center is in the form of a solid solution, whereas the particle on the far right shows the Rh- and Pt-rich patches (partly segregated). Complete mixing (solid solution) is observed in the nanoparticles imaged at the 3:03 time-point. Time-points 3:16 and 3:37 show partial segregation, while the two last time-points, 3:49 and 3:58 show only solid solution. In summary, the sizes of the two fully segregated nanoparticles are 11 and 13 nm, although these correspond to the two first time-points after reaching 600 °C and these particles may not have had sufficient time to mix at this time-point. The average size of the seven partly segregated nanoparticles was D_{avg} = 15.0 ± 2.6 nm (size range 12 – 19 nm). The sizes of the nanoparticles with signs of segregation are thus close to, or above, the lower size limit (~ 13 nm) we observed for segregated particles in the 650 °C experiment (manuscript section 1). The average nanoparticle size of the five solid solution particles was D_{avg} = 15.1 ± 3.0 nm (range 10 – 18 nm).

We would like to point out that the high-resolution HAADF-STEM images showed the nanoparticles at all time-points at 600 °C to be crystalline.

The chemical composition of individual (NP6 – 19) Rh(core)-Pt(shell) nanoparticles at 600 °C was determined by quantitative EDS analysis. The average composition of the nanoparticles (± 1σ) normalized to Pt and Rh was 51.6 ± 7.3 at. % Pt. The areas selected for analysis are shown in Fig. S26 and the results are summarized in Table S8, as a function of time progressed from the point when 600 °C was reached. The Pt content of the individual nanoparticles was plotted versus nanoparticle size (Fig. S27a), and no correlation between the two parameters was identified. The Pt content of the individual nanoparticles was also plotted versus time dwelled at 600 °C (Fig. S27b). The first time point (35 min) corresponds to a higher Pt-content than all later points, but note that this is only one data point. From the data set as a whole, we conclude that no correlation between the chemical composition and time progressed at 600 °C exists. The type of elemental distribution (S = segregated; PS = partly segregated; SS = solid solution) was plotted versus the chemical composition (Fig. S27c). One data point belonging to the segregated (S) category shows a higher Pt content. We conclude there is no clear correlation between the type of elemental distribution and composition in the composition range studied here. We discussed the type of elemental distribution versus nanoparticle size at the beginning of this section, and this is plotted in Fig. S27d.

Overall, we conclude that the Rh(core)-Pt(shell) nanoparticles undergo elemental mixing to either the solid solution or partly segregated configuration at 600 °C, as all nanoparticles studied are clearly different compared to the starting situation at room temperature.

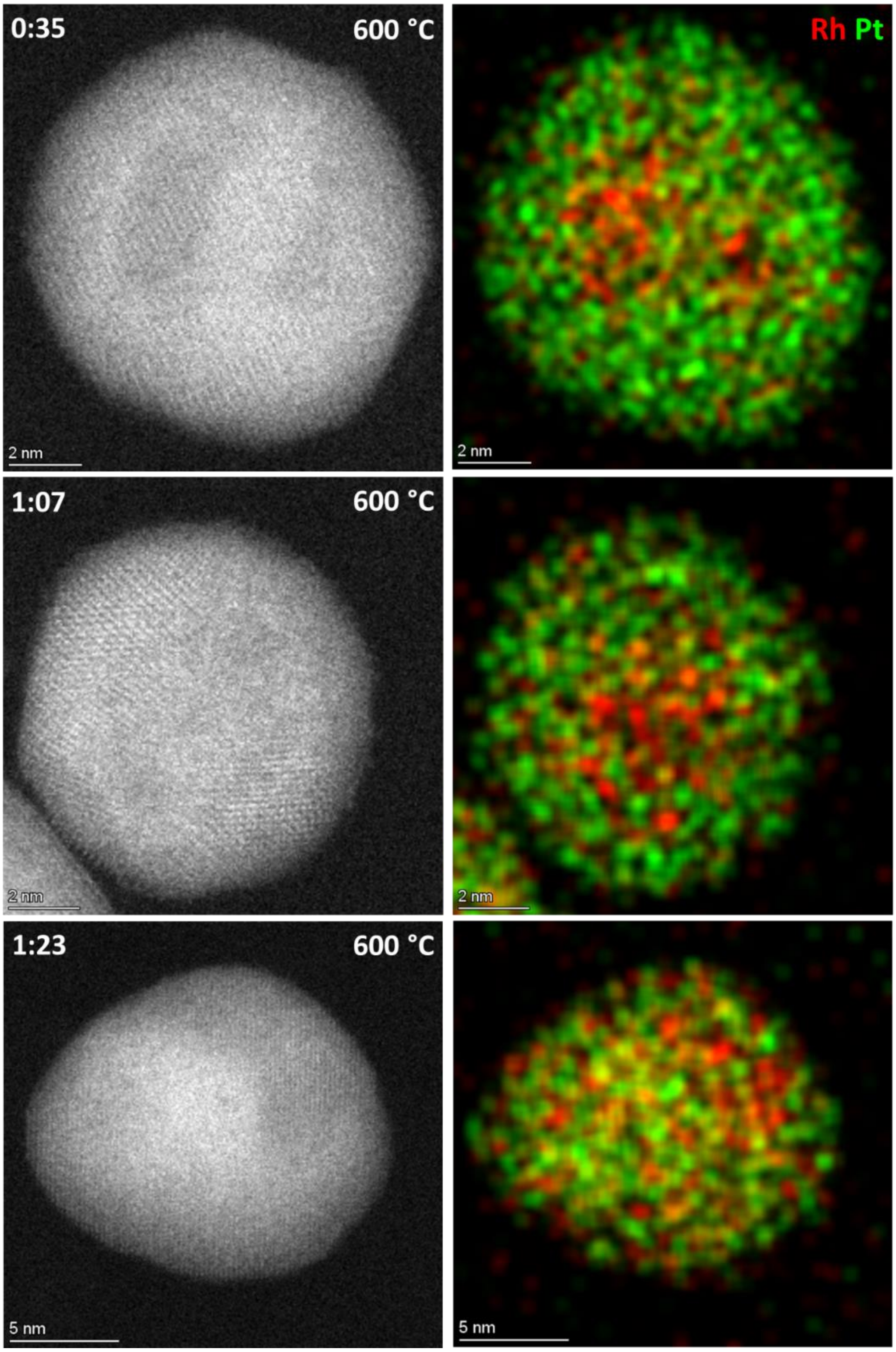

0:35
600 °C
2 nm
Rh Pt
2 nm
1:07
600 °C
2 nm
2 nm
1:23
600 °C
5 nm
5 nm

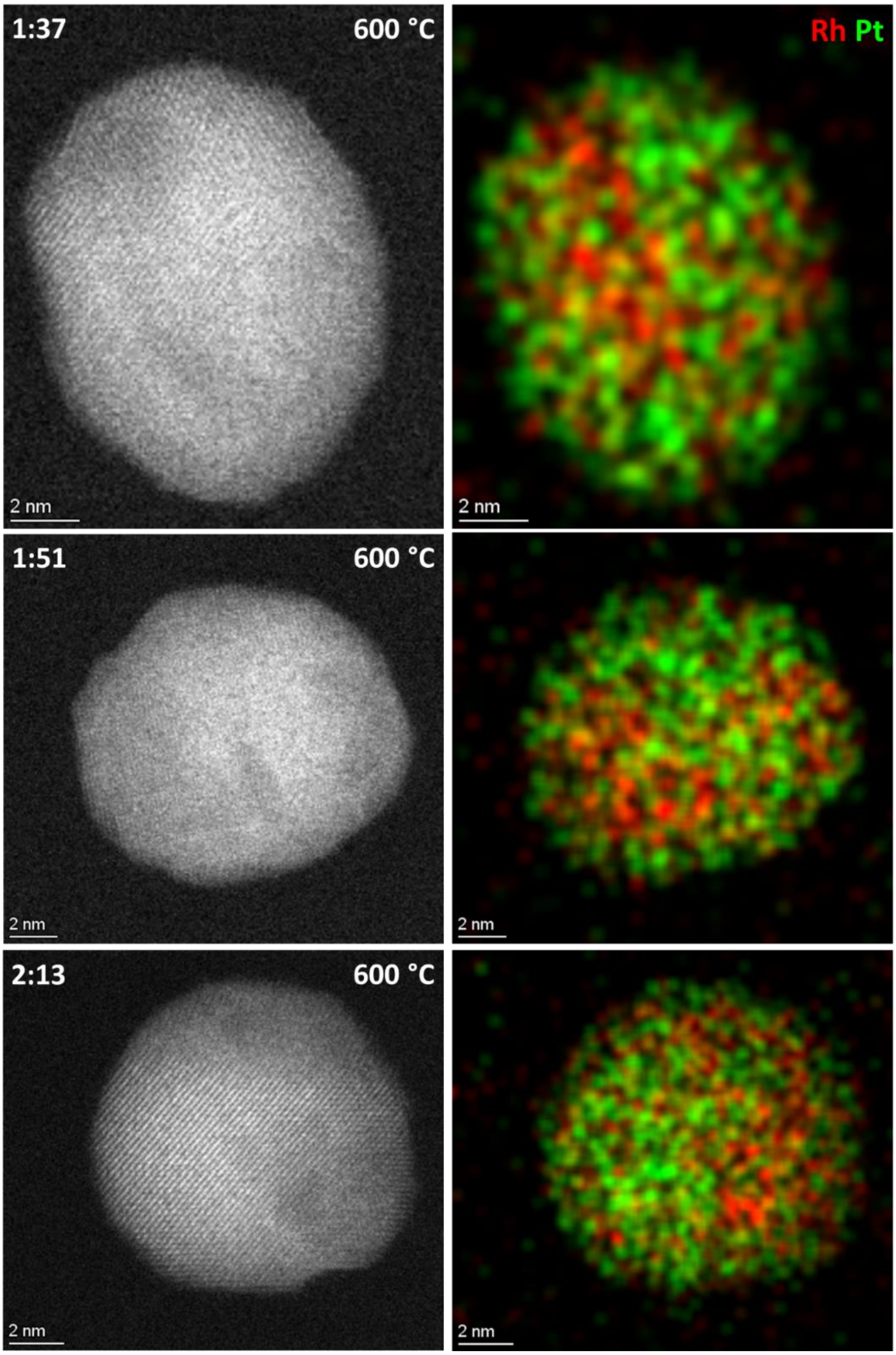

1:37
600 °C
Rh Pt
2 nm
2 nm
1:51
600 °C
2 nm
2 nm
2:13
600 °C
2 nm
2 nm

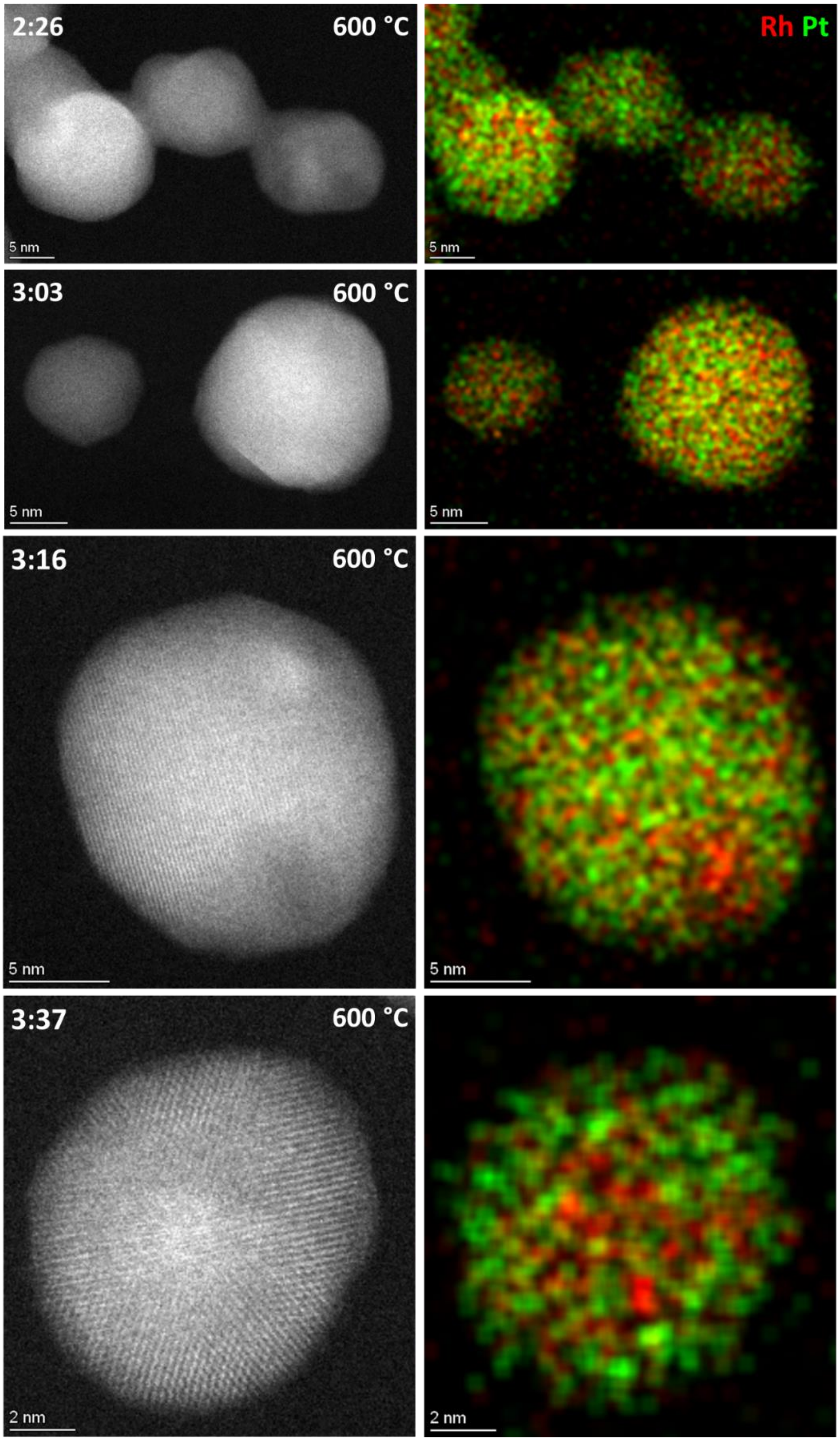
2:26
600 °C
Rh Pt
5 nm
5 nm
3:03
600 °C
5 nm
5 nm
3:16
600 °C
5 nm
5 nm
3:37
600 °C
2 nm
2 nm

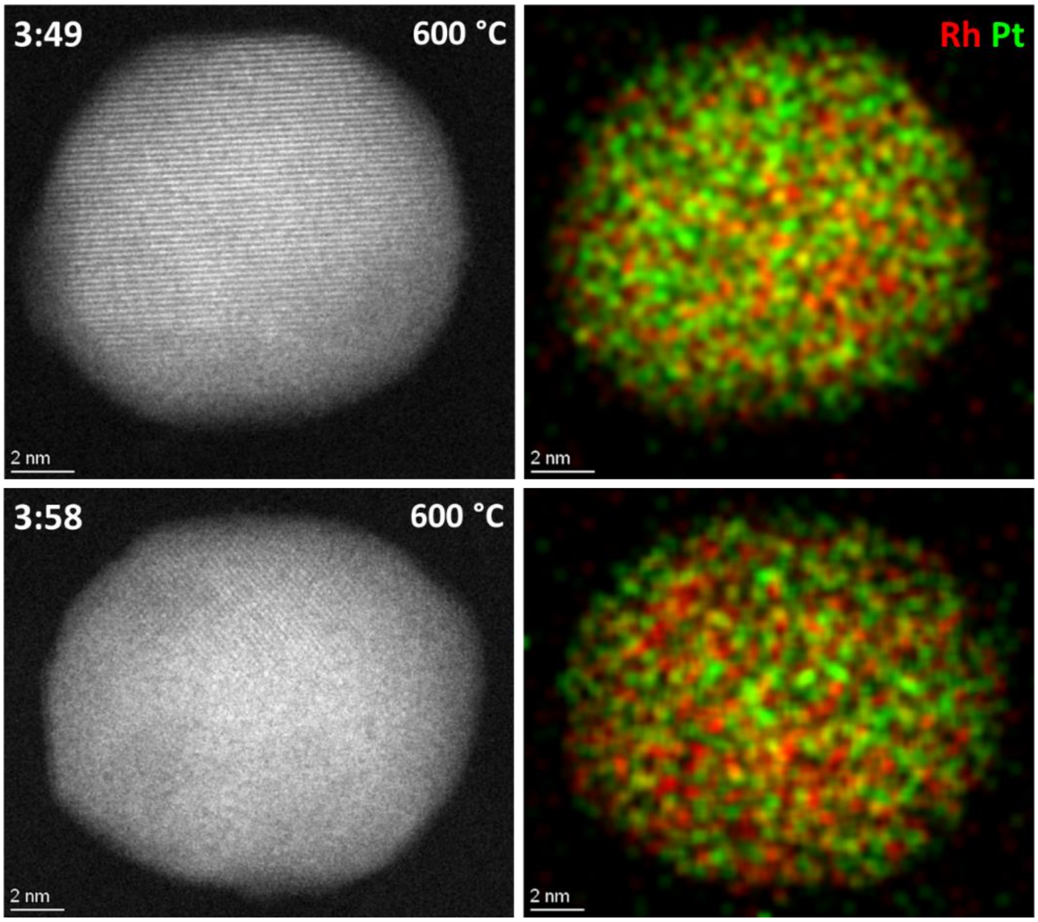

Fig. S25 Rh(core)-Pt(shell) nanoparticles imaged with HAADF-STEM and EDS elemental maps at 600 °C. The time after the temperature has reached 600 °C is indicated by h:min. Rh and Pt are mapped in red and green, respectively.

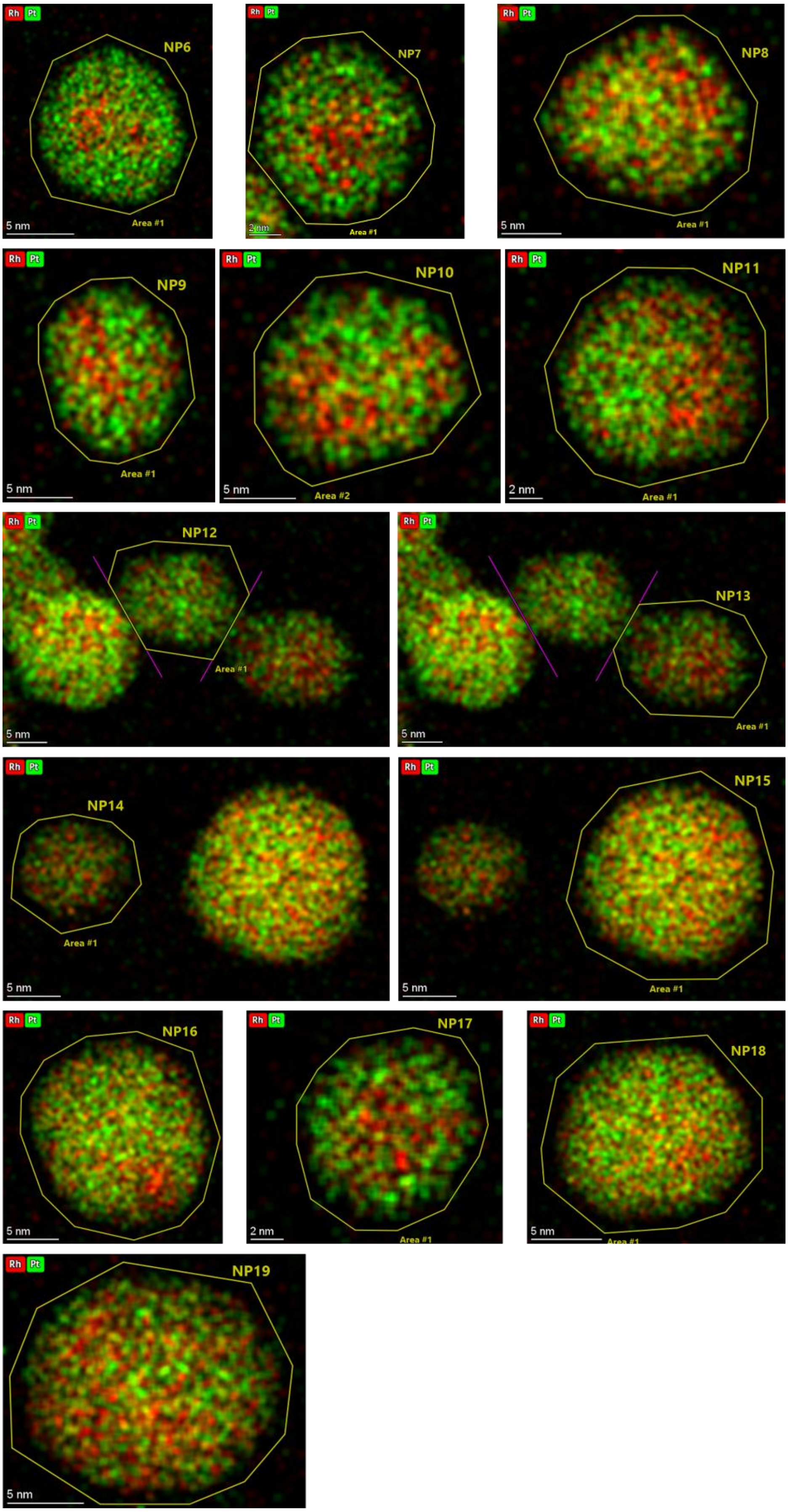

Fig. S26 **EDS elemental maps of Rh(core)-Pt(shell) nanoparticles (NP6-19) at 600 °C showing the areas selected for quantitative EDS analysis (results in Table S8). Rh and Pt are mapped in red and green, respectively.**

Table S8 Results from quantitative EDS analysis of Rh(core)-Pt(shell) nanoparticles (NP6-19) at 600 °C (see Fig. S26). The Pt-Rh molar composition is normalized to 100 at. %.

Nanoparticle	Time at 600 °C [h: mm]	Rh [at. %]	Pt [at. %]	Nanoparticle size [nm]	Element distribution
NP6	0:35	29 ±2.5	71 ±2.5	12.7	Segregated
NP7	1:07	42 ±2.9	58 ±2.9	11.0	Segregated
NP8	1:23	53 ±3.0	47 ±3.0	17.2	Partly segregated
NP9	1:37	49 ±3.0	51 ±3.0	13.6	Partly segregated
NP10	1:51	46 ±2.9	54 ±2.9	14.8	Partly segregated
NP11	2:13	51 ±3.0	49 ±3.0	12.2	Partly segregated
NP12	2:26	44 ±2.9	56 ±2.9	15.5	Solid solution
NP13	2:26	55 ±2.9	45 ±2.9	16.2	Partly segregated
NP14	3:03	48 ±3.0	52 ±3.0	10.4	Solid solution
NP15	3:03	50 ±3.0	50 ±3.0	18.1	Solid solution
NP16	3:16	48 ±3.0	52 ±3.0	18.9	Partly segregated
NP17	3:37	60 ±2.9	40 ±2.9	12.0	Partly segregated
NP18	3:49	48 ±3.0	52 ±3.0	14.3	Solid solution
NP19	3:58	55 ±2.9	45 ±2.9	17.2	Solid solution

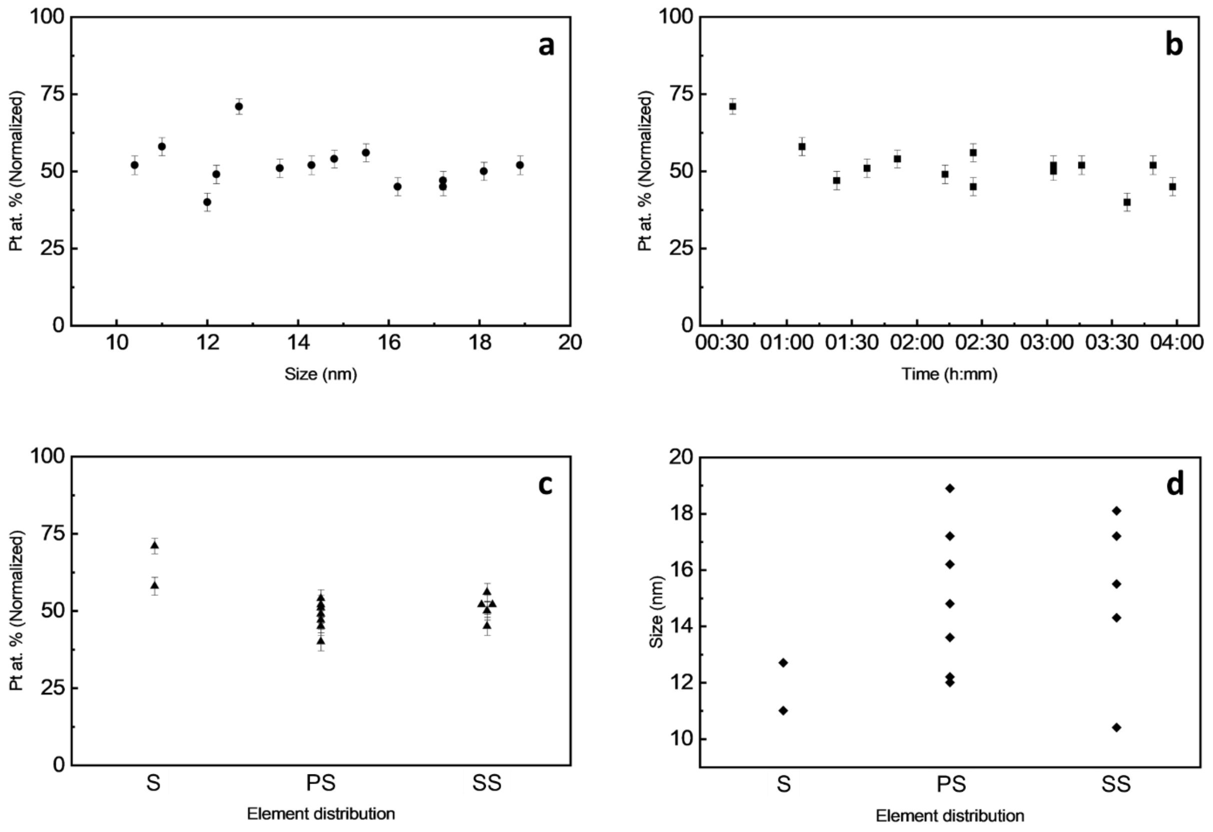

Fig. S27 Normalized Pt content from quantitative EDS results (Table S8) versus Rh(core)-Pt(shell) nanoparticle size (a), time (hh:mm) dwelled at the targeted temperature (b), elemental distribution type (c), and nanoparticle size versus elemental distribution type (d) at 600 °C. The Pt-Rh molar composition is normalized to 100 at. %. The elemental distribution type is categorized as segregated (S), partly segregated (PS), and solid solution (SS).

3.3. Larger Rh(core)-Pt(shell) nanoparticles at 300 °C after cooling from 600 °C

The analysis of Rh(core)Pt-(shell) nanoparticles (batch 2) cooled (during 1.5 h) from 600 °C to the current temperature of 300 °C in vacuum are described in this section. As done in the previous section (3.2) at the higher temperature, we examine the element distribution by HAADF-STEM images and corresponding EDS elemental maps as time progresses at 300 °C.

In total, 15 nanoparticles (D_{avg} = 15.2 ± 2.9 nm) were analyzed, and the HAADF-STEM images and EDS maps are reported in Fig. S28. In the 1:12 time-point after reaching 300 °C, we observe one nanoparticle (far right) which has a distinct Rh-rich component (segregated). Importantly, this component is defined more clearly than observed in the sample at 600 °C after the time-point of 1:37. The two other particles at time-point 1:12 at 300 °C, however, show complete mixing (solid solution) of Pt and Rh. The same distinct Rh-rich patch can be observed in one of the two particles in the image captured at 1:25 (segregated), while the other is in the form of a solid solution. In the slightly zoomed-out image at 1:38, similar small patches of Rh-rich areas can be seen inside at least three of the particles (the top one, and the two on the left in the cluster of three at the bottom right) when viewing the dark areas in the HAADF-STEM image in conjunction with the EDS map, which is the reason why they classify as segregated. The other two particles in the image at 1:38 show complete mixing of Pt and Rh (solid solution). The particles at time-points 1:49 and 1:57 are solid solutions, as evident from the uniform contrast in the HAADF-STEM images and the absence of Rh- or Pt-rich areas in the EDS maps. At time-point 2:09, one of the two nanoparticles again shows a Rh rich area (partly segregated). The other nanoparticle at this time-point is in the solid solution configuration. Overall, we observed nine solid solution nanoparticles (D_{avg} = 15.8 ± 3.3 nm, size range 11 – 21 nm), one partly segregated nanoparticle (17.0 nm) and five segregated nanoparticles (13.7 ± 1.8 nm, size range 12 – 16 nm).

The chemical composition of a selection (NP20 – 30) of individual Rh(core)-Pt(shell) nanoparticles at 300 °C after cooldown from 600 °C was determined by quantitative EDS analysis. The areas selected for analysis are shown in Fig. S29 and the results are summarized in Table S9. The average composition of the nanoparticles (± 1σ) normalized to Pt and Rh was 53.6 ± 4.8 at. % Pt. The Pt content of the individual nanoparticles was plotted versus nanoparticle size (Fig. S30a). No correlation between nanoparticle size and Pt content was identified. The individual nanoparticle Pt content was also plotted versus dwell time at 300 °C (Fig. S30b). No correlation was identified between these two parameters. In Fig. S30c, the type of elemental distribution (S = segregated; PS = partly segregated; SS = solid solution) was plotted versus Pt content. No correlations between the two parameters were identified. The type of elemental distribution versus nanoparticle size was discussed at the beginning of the section and is plotted for NP20 – 30 in Fig. S30d.

Overall, the nanoparticles have started to show signs of more distinct segregation of Pt and Rh at 300 °C, as compared to 600 °C. Further, these observations are independent of the Pt content and nanoparticle sizes studied.

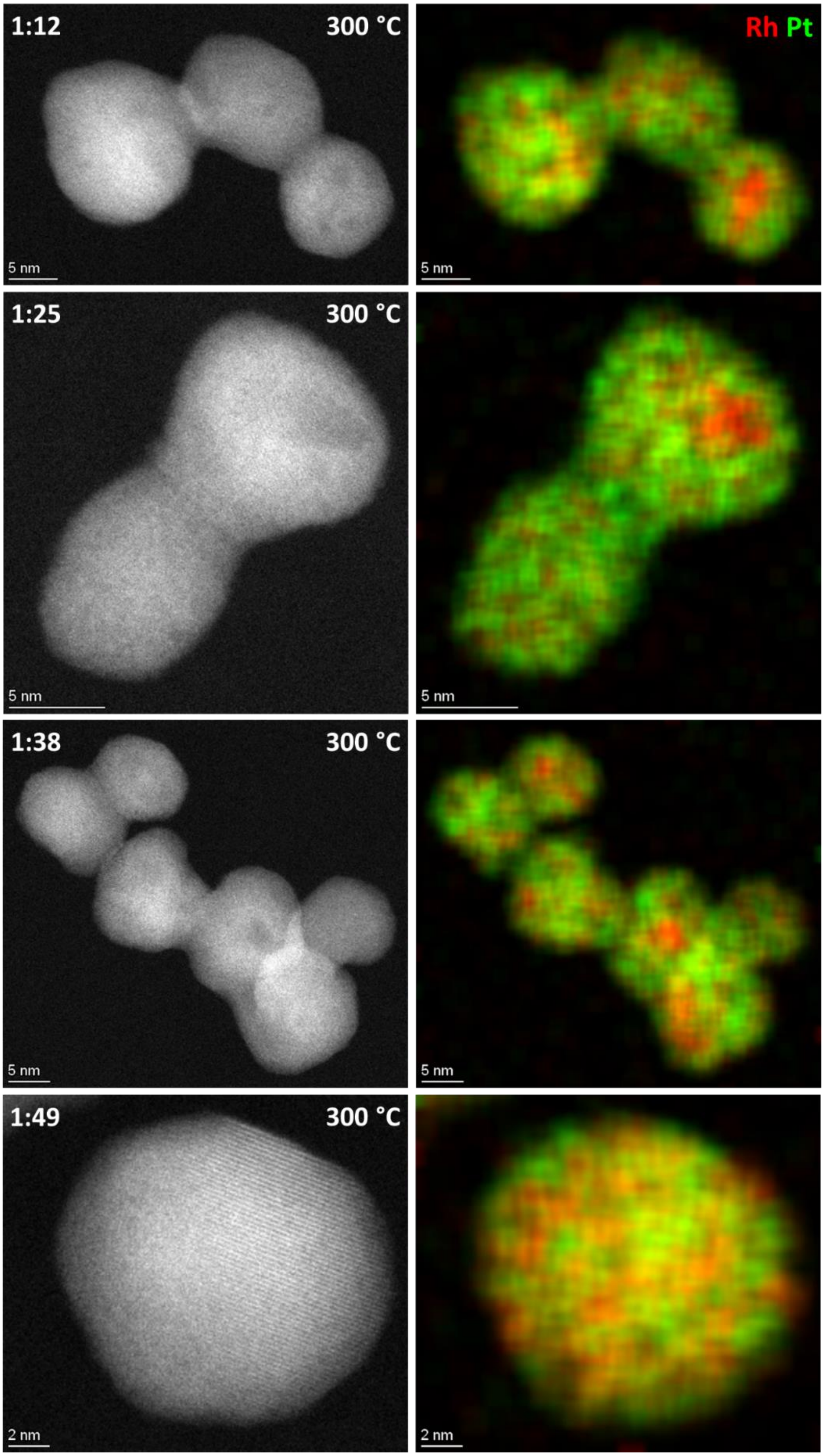
1:12
300 °C
Rh Pt
5 nm
5 nm
1:25
300 °C
5 nm
5 nm
1:38
300 °C
5 nm
5 nm
1:49
300 °C
2 nm
2 nm

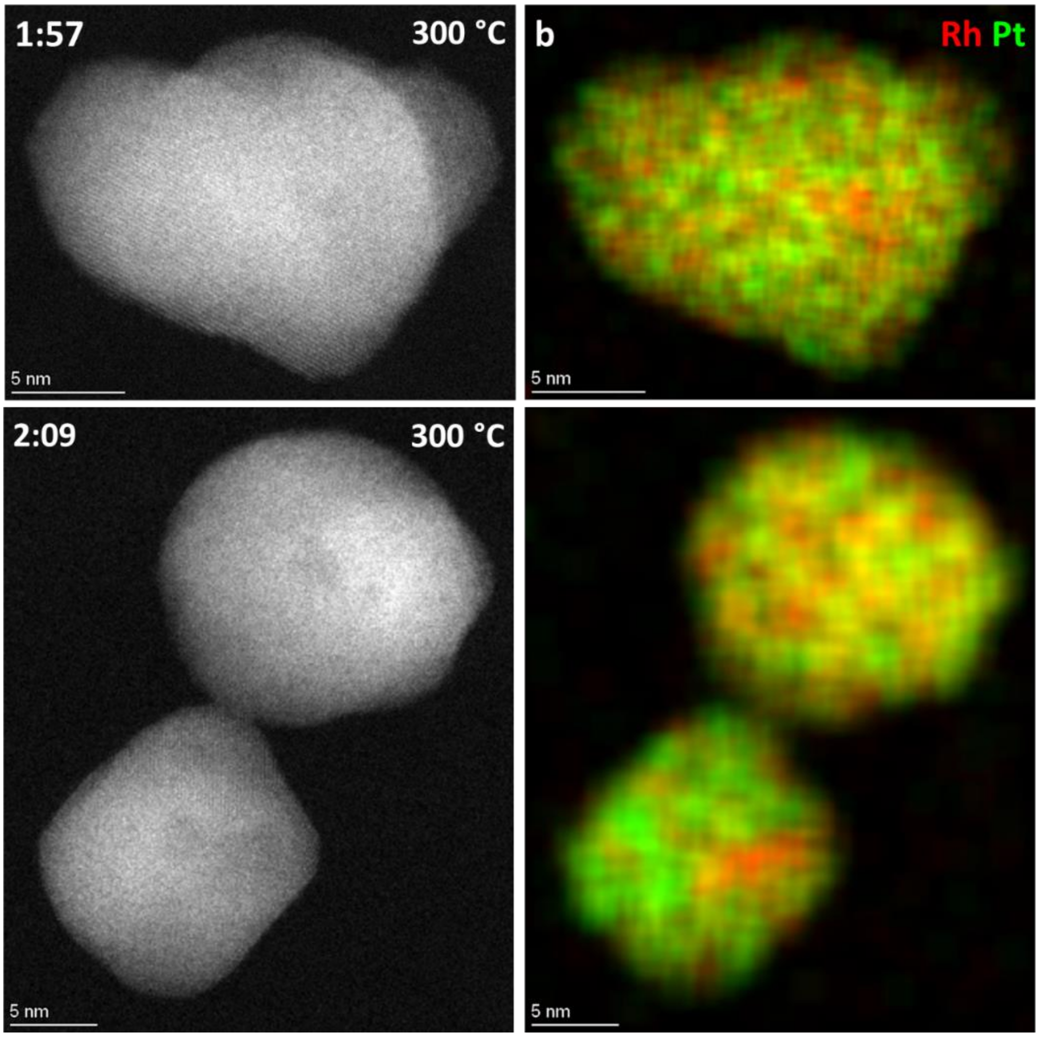

Fig. S28 **Rh(core)-Pt(shell) nanoparticles imaged with HAADF-STEM and EDS elemental maps at 300 °C after cooldown from 600 °C. The time after the temperature has reached 300 °C is indicated by h:min. Rh and Pt are mapped in red and green, respectively.**

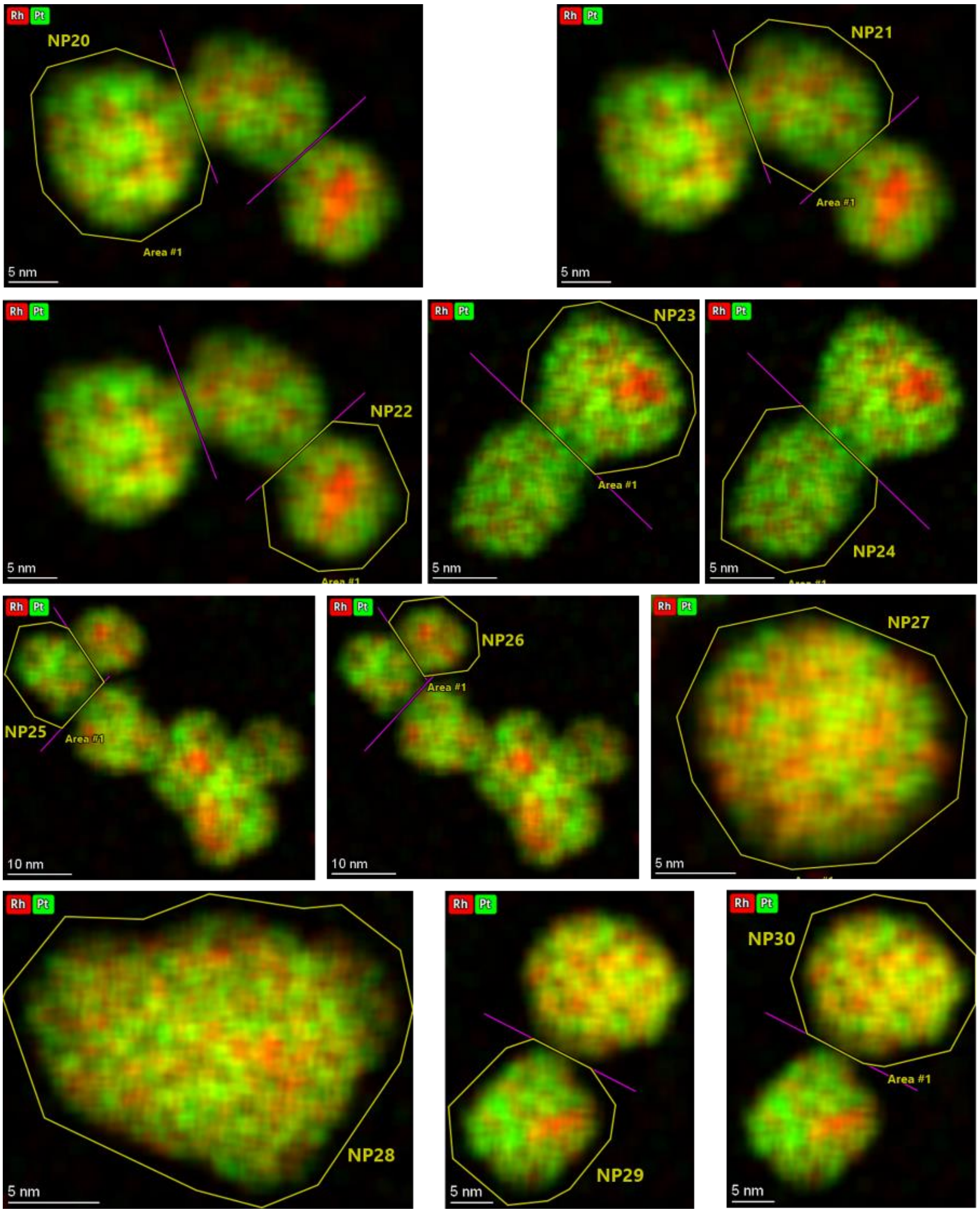

Fig. S29 EDS elemental maps of Rh(core)-Pt(shell) nanoparticles (NP20-30) at 300 °C after cooldown from 600 °C showing the areas selected for quantitative EDS analysis (results in Table S9). Rh and Pt are mapped in red and green, respectively.

Table S9 Results from quantitative EDS analysis of Rh(core)-Pt(shell) nanoparticles (NP6-19) at 300 °C after cooldown from 600 °C (see Fig. S29). The Pt-Rh molar composition is normalized to 100 at. %.

Nanoparticle	Time at 300 °C [h: mm]	Rh [at. %]	Pt [at. %]	Nanoparticle size [nm]	Element distribution
NP20	1:12	41 ±2.9	59 ±2.9	18.0	Solid solution
NP21	1:12	41 ±2.9	59 ±2.9	15.8	Solid solution
NP22	1:12	48 ±3.0	52 ±3.0	12.7	Segregated
NP23	1:25	52 ±3.0	48 ±3.0	12.6	Segregated
NP24	1:25	44 ±2.9	56 ±2.9	11.4	Solid solution
NP25	1:38	41 ±2.9	59 ±2.9	14.0	Solid solution
NP26	1:38	53 ±3.0	47 ±3.0	12.0	Segregated
NP27	1:49	49 ±3.0	51 ±3.0	16.6	Solid solution
NP28	1:57	51 ±3.0	49 ±3.0	20.9	Solid solution
NP29	2:09	41 ±2.9	59 ±2.9	17.0	Partly segregated
NP30	2:09	49 ±3.0	51 ±3.0	19.4	Solid solution

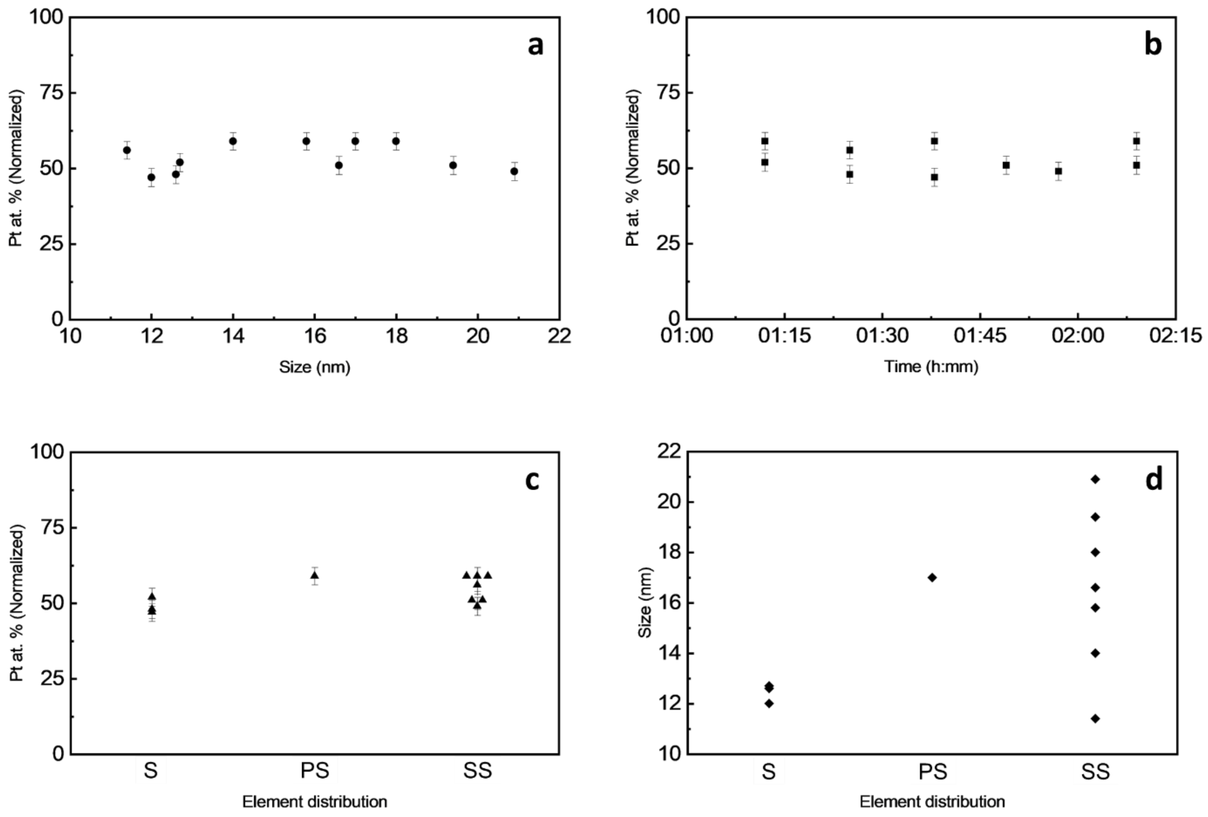

Fig. S30 Normalized Pt content from quantitative EDS results (Table S9) versus Rh(core)-Pt(shell) nanoparticle size (a), time (hh:mm) dwelled at the targeted temperature (b), elemental distribution type (c), and nanoparticle size versus elemental distribution type (d) at 300 °C after cooldown from 600 °C. The Pt-Rh molar composition is normalized to 100 at. %. The elemental distribution type is categorized as segregated (S), partly segregated (PS), and solid solution (SS).

3.4. Rh(core)-Pt(shell) nanoparticles at room temperature after cooling from 300 °C

An analysis of the Rh(core)Pt-(shell) nanoparticles heated to 600 °C, then cooled (during 1.5 h) to 300 °C before finally being cooled (during 1.5 h) to room temperature in vacuum is presented here (Fig. S31). In this section, we examine the element distribution from HAADF-STEM images and EDS elemental maps versus dwell time at room temperature.

In total, 19 nanoparticles (D_{avg} = 15.2 ± 3.7 nm) were analyzed based on the HAADF-STEM data presented in Fig. S31. The two nanoparticles corresponding to the two first time-points, at 0:04 (24.4 nm) and 0:18 (19.3 nm) after reaching room temperature, are both in the solid solution configuration. One nanoparticle (15.6 nm) is partly segregated (2:43). Strikingly, all other (16) nanoparticles are fully segregated with D_{avg} = 14.3 ± 3.0 nm and size range 9 – 21 nm. The Rh-rich areas are notably always surrounded by Pt-rich layers in the segregated nanoparticles. For clarity, in the manuscript, we reported the average nanoparticle size of all particles showing signs of segregation (partly- and fully segregated), with D_{avg} = 14.4 ± 2.9 nm (size range 9 – 21 nm). This is strong evidence that the vast majority of the nanoparticles has returned to their original Rh(core)-Pt(shell) elemental distribution configuration they had before any heat treatment, although with off-centered Rh-cores (see Discussion section in the manuscript). We note that some of the segregated nanoparticles we measured as individual nanoparticles, including the smallest one at 9 nm, are positioned in close vicinity to another nanoparticle and have to some extent sintered to these. Some mass transport has thus occurred between these particles. Therefore, it is unclear whether these should be interpreted as "individual nanoparticles" with respect to the observed elemental distribution versus nanoparticle size.

The chemical composition of a selection (NP31 – 42) of individual nanoparticles in the sample was determined by quantitative EDS analysis. The areas selected for analysis are shown in Fig. S32 and the results are summarized in Table S10. The average composition of the nanoparticles (± 1σ) normalized to Pt and Rh was 48.6 ± 8.9 at. % Pt. The Pt content for the individual nanoparticles was plotted versus nanoparticle size (Fig. S33a) to determine possible correlations between the two parameters. No correlation was identified. The Pt content of the individual nanoparticles was then plotted versus dwell time at room temperature (Fig. S33b). No obvious correlation was identified between these two parameters. Next, the type of elemental distribution (S = segregated; PS = partly segregated; SS = solid solution) was plotted versus the Pt content of the individual nanoparticles (Fig. S33c). No correlations between the two parameters were identified. At the beginning of this section, we discussed the type of elemental distribution versus nanoparticle size. For NP31 – 42, this is plotted in Fig. S33d.

Overall, the HAADF-STEM and EDS elemental maps show clear evidence of segregation of Pt and Rh occurring when the temperature is lowered to room temperature from 300 °C in the larger Pt-Rh nanoparticles. These observations are independent of Pt content in the composition range studied.

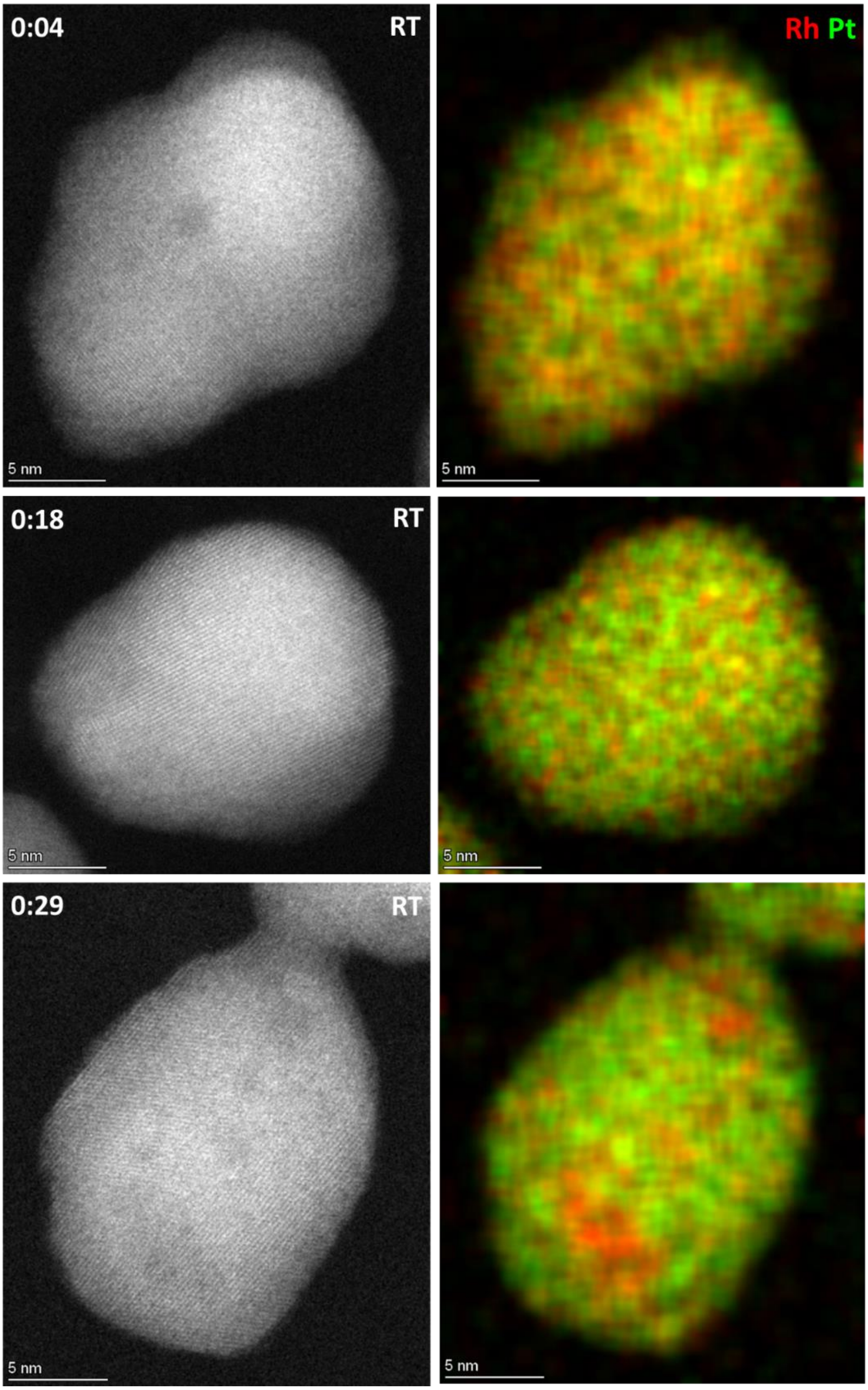
0:04
RT
5 nm
Rh Pt
5 nm
0:18
RT
5 nm
5 nm
0:29
RT
5 nm
5 nm

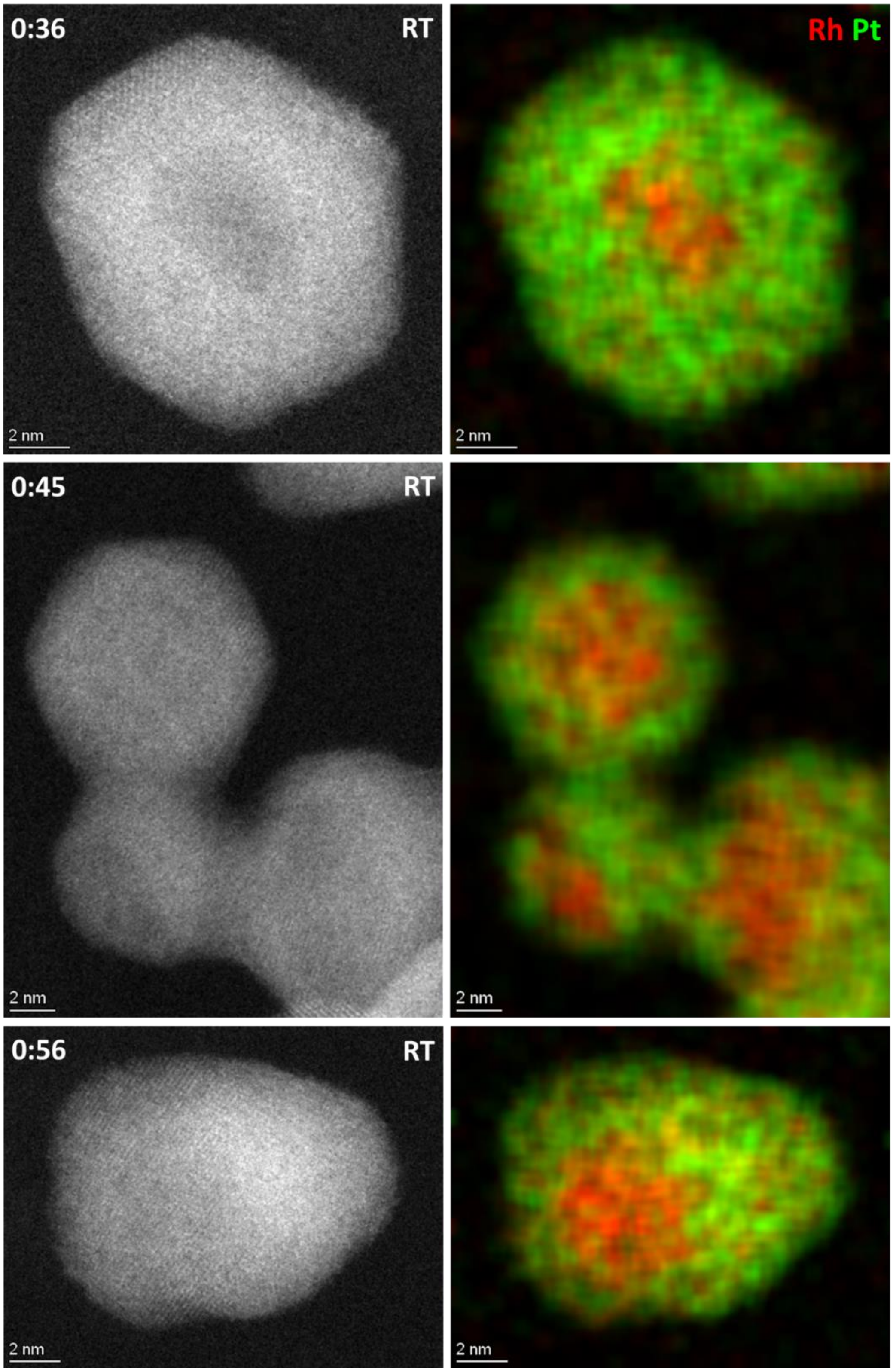
0:36
RT
Rh Pt
2 nm
2 nm
0:45
RT
2 nm
2 nm
0:56
RT
2 nm
2 nm

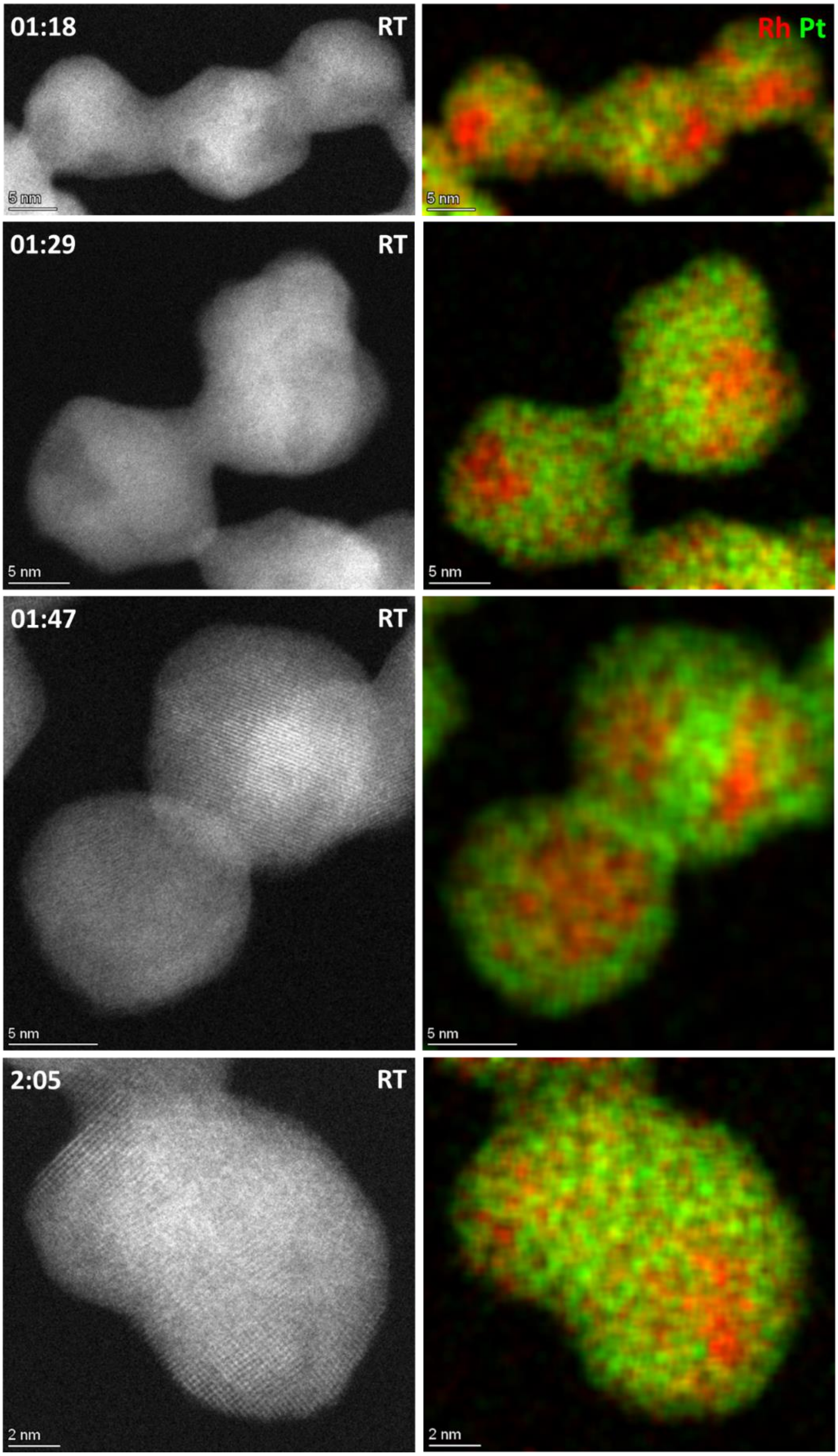
01:18
RT
5 nm
Rh Pt
5 nm
01:29
RT
5 nm
5 nm
01:47
RT
5 nm
5 nm
2:05
RT
2 nm
2 nm

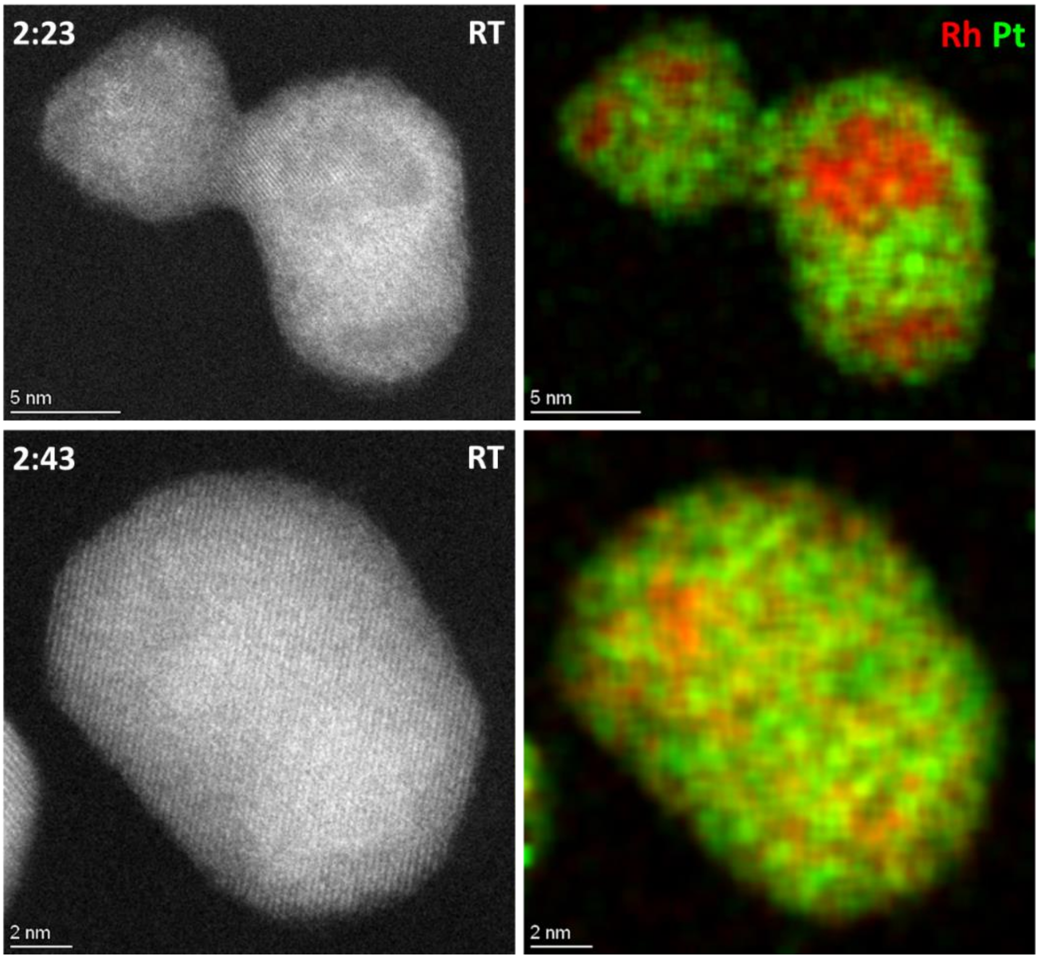

Fig. S31 **Rh(core)-Pt(shell) nanoparticles imaged with HAADF-STEM and EDS elemental maps at room temperature after cooldown from 300 °C. The time after the targeted temperature is reached is indicated by h:min. Rh and Pt are mapped in red and green, respectively.**

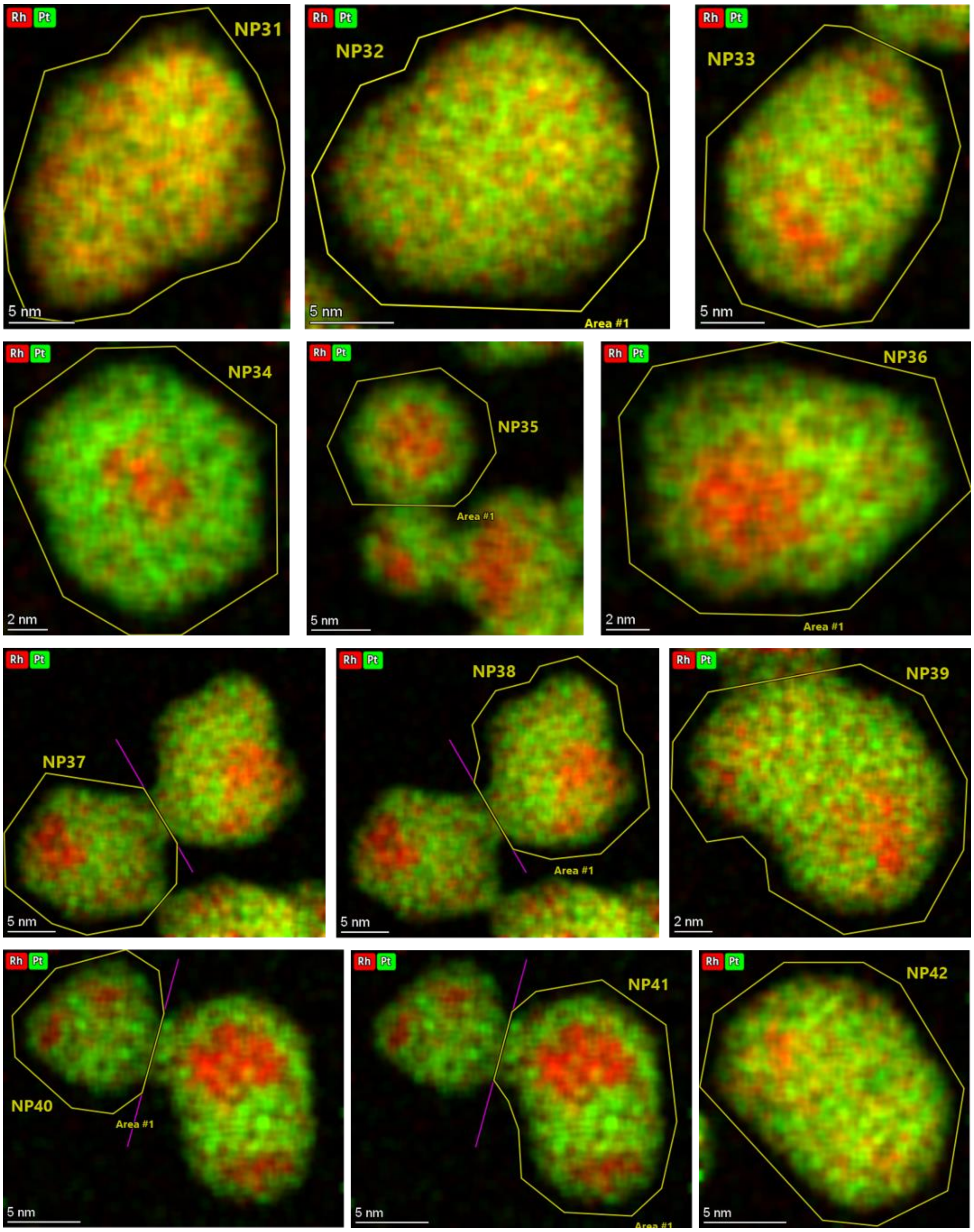

Fig. S32 EDS elemental maps of Rh(core)-Pt(shell) nanoparticles (NP31-42) at room temperature after cooldown from 300 °C showing the areas selected for quantitative EDS analysis (results in Table S10). Rh and Pt are mapped in red and green, respectively.

Table S10 Results from quantitative EDS analysis of Rh(core)-Pt(shell) nanoparticles (NP31-42) at room temperature after cooldown from 300 °C (see Fig. S32). The Pt-Rh molar composition is normalized to 100 at. %.

Nanoparticle	Time at RT [h: mm]	Rh [at. %]	Pt [at. %]	Nanoparticle size [nm]	Element distribution
NP31	0:04	53 ±2.9	47 ±2.9	24.4	Solid solution
NP32	0:18	52 ±3.0	48 ±3.0	19.3	Solid solution
NP33	0:29	45 ±2.9	55 ±2.9	20.5	Segregated
NP34	0:36	32 ±2.6	68 ±2.6	13.3	Segregated
NP35	0:45	53 ±3.0	47 ±3.0	11.9	Segregated
NP36	0:56	53 ±2.9	47 ±2.9	14.2	Segregated
NP37	1:29	51 ±3.0	49 ±3.0	16.4	Segregated
NP38	1:29	51 ±3.0	49 ±3.0	18.8	Segregated
NP39	2:05	50 ±3.0	50 ±3.0	14.5	Segregated
NP40	2:23	60 ±2.8	40 ±2.8	9.1	Segregated
NP41	2:23	70 ±2.5	30 ±2.5	14.6	Segregated
NP42	2:43	47 ±2.9	53 ±2.9	15.6	Partly segregated

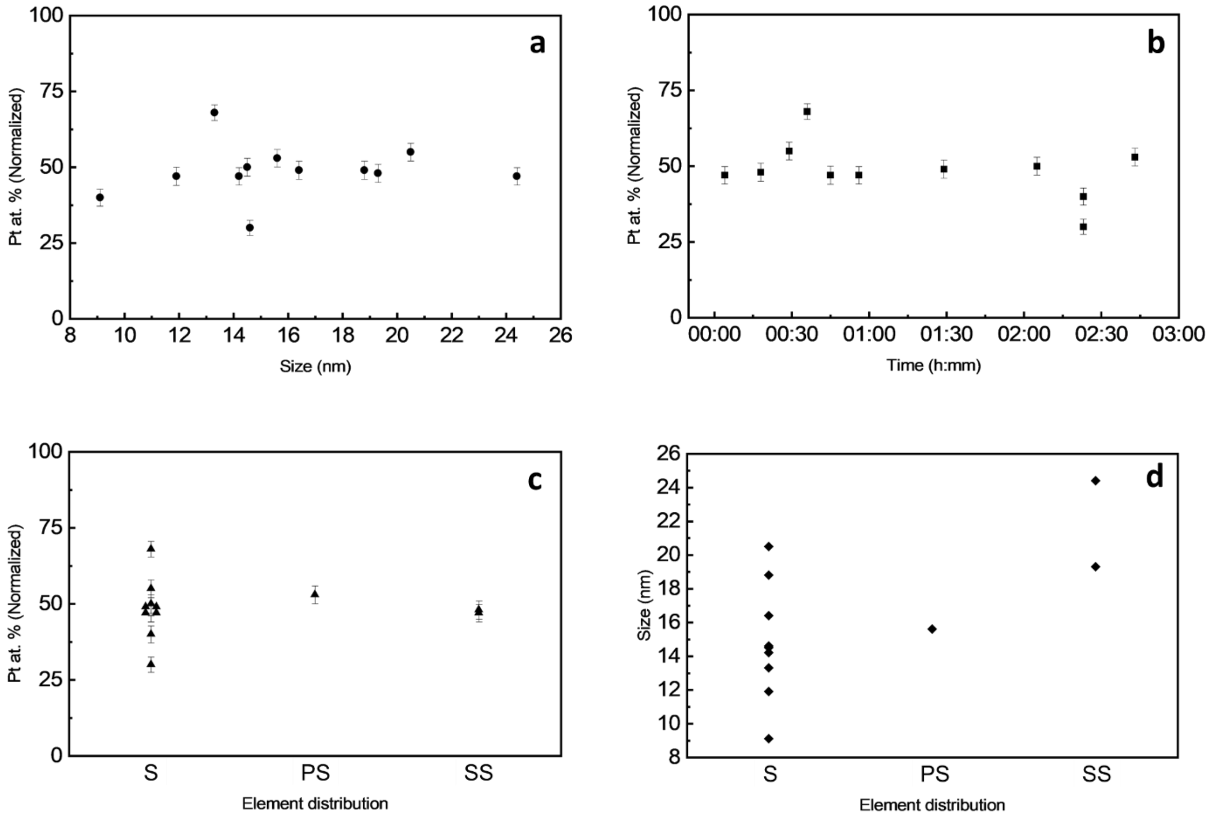

Fig. S33 Normalized Pt content from quantitative EDS results (Table S10) versus Rh(core)-Pt(shell) nanoparticle size (a), dwell time (hh:mm) at the targeted temperature (b), elemental distribution type (c), and nanoparticle size versus elemental distribution type (d) at room temperature after cooldown from 300 °C. The Pt-Rh molar composition is normalized to 100 at. %. The elemental distribution type is categorized as segregated (S), partly segregated (PS), and solid solution (SS).

3.5. Rh(core)-Pt(shell) nanoparticles after heating, then after 11 days at room temperature

We went back to the Rh(core)Pt-(shell) nanoparticle sample (batch 2) after first heating to 600 °C, followed by cooling to room temperature via dwelling at 300 °C (see Fig. S21), then stored under vacuum for 11 days.

We analyzed the 19 nanoparticles presented in Fig. S34. We find clear evidence from both the HAADF-STEM images and the EDS elemental maps that all the nanoparticles (D_{avg} = 14.9 ± 2.9 nm, size range 11 – 22 nm) are in the Rh(core)-Pt(shell) configuration. Note that some of the analyzed nanoparticles were sintered to other nanoparticles, implying mass transport. Therefore, it is unclear whether these should be interpreted as "individual nanoparticles", which is how we measured them, with respect to the observed element distribution versus nanoparticle size.

The chemical composition of a selection (NP43 – 47) of individual Rh(core)-Pt(shell) nanoparticles after being stored in vacuum at room temperature for 11 days was determined using quantitative EDS analysis. The areas selected for analysis are shown in Fig. S35 and the results are summarized in Table S11. The average composition of the nanoparticles (± 1σ) normalized to Pt and Rh was 49.4 ± 5.6 at. % Pt. The Pt content of the individual nanoparticles was plotted versus nanoparticle size (Fig. S36) to look for possible correlations between the two parameters. No correlations were identified.

Overall, the HAADF-STEM and EDS elemental maps show all nanoparticles to be segregated with respect to Pt and Rh, in the form of a Rh(core)-Pt(shell) configuration, after being stored at room temperature in vacuum for 11 days after the heat treatment.

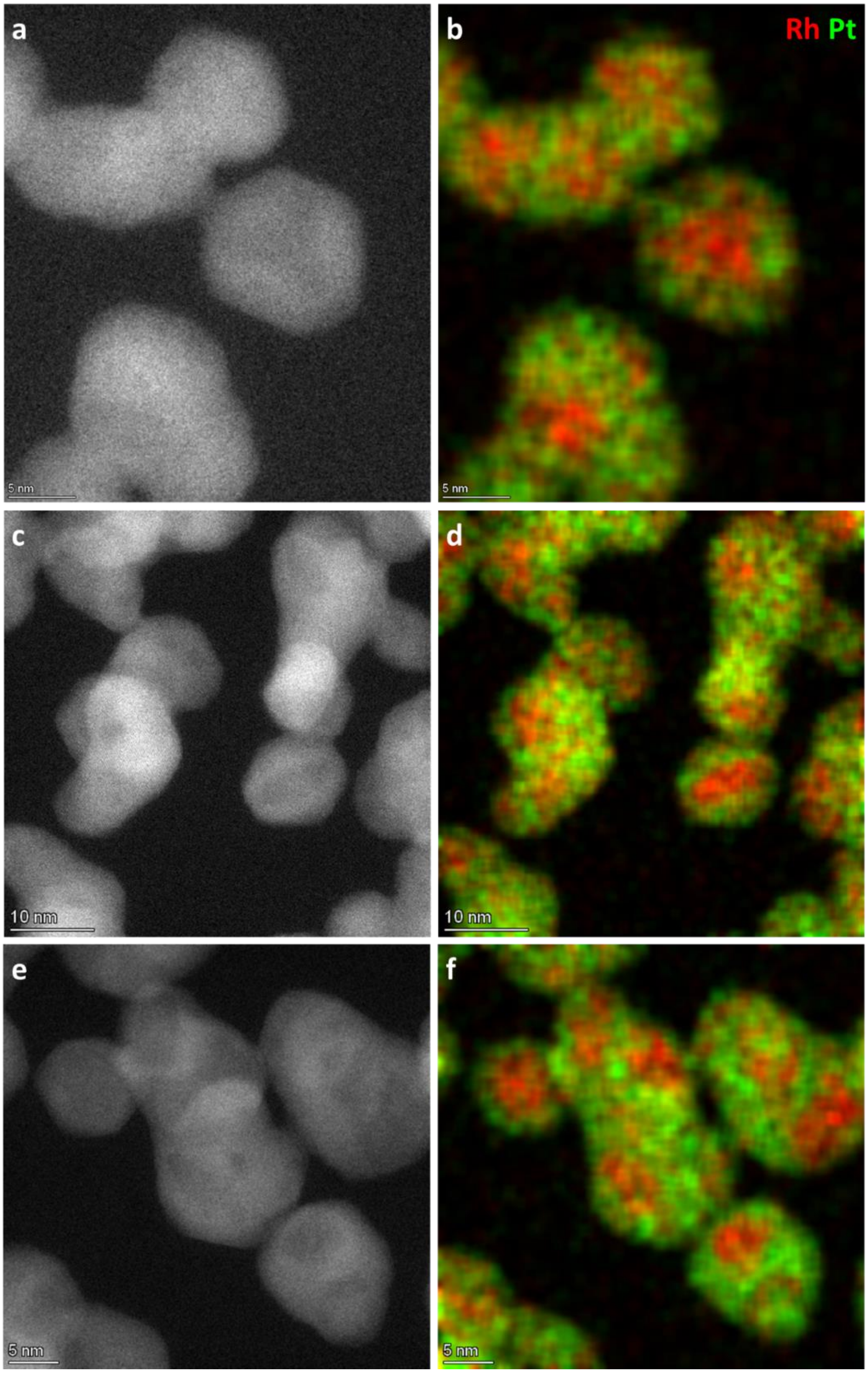
a
b
Rh Pt
5 nm
5 nm
c
d
10 nm
10 nm
e
f
5 nm
5 nm

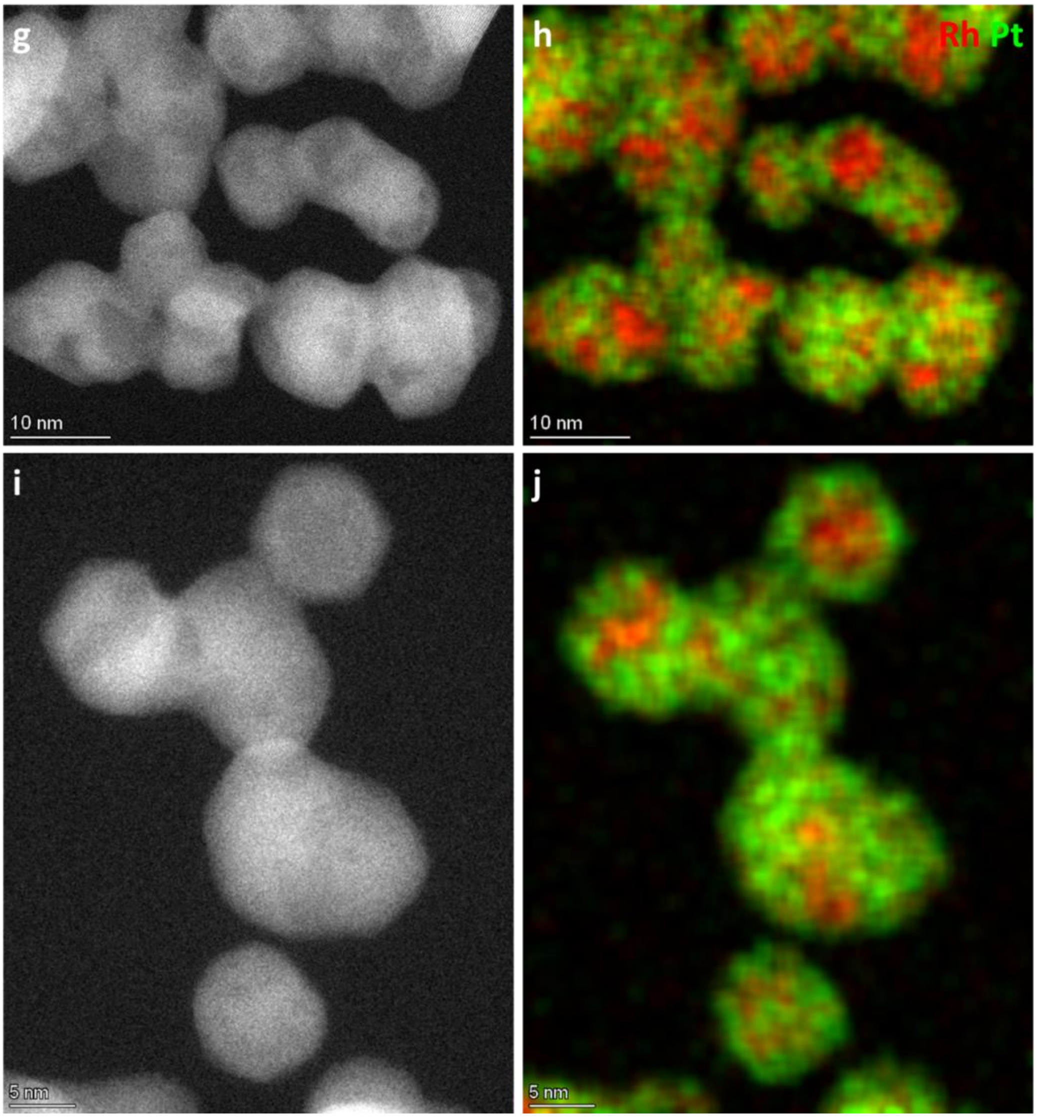

Fig. S34 **Rh(core)-Pt(shell) nanoparticles imaged with HAADF-STEM and EDS elemental maps at room temperature after being stored for 11 days in vacuum after cooldown from 300 °C. Rh and Pt are mapped in red and green, respectively.**

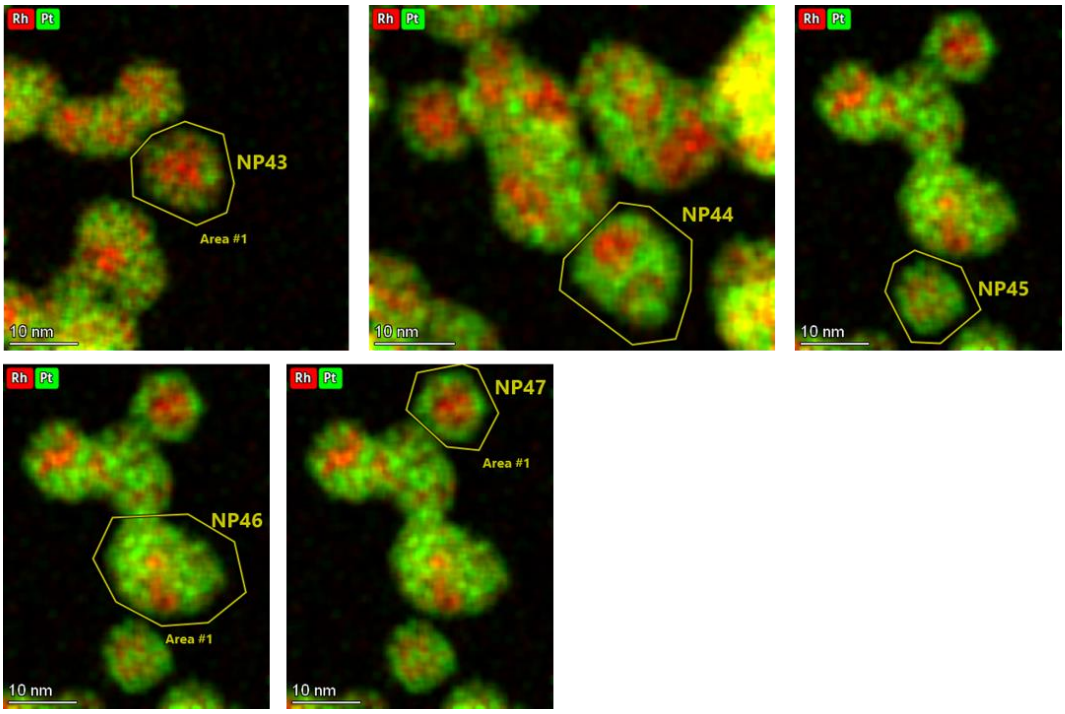

Fig. S35 **EDS elemental maps of Rh(core)-Pt(shell) nanoparticles (NP43-47) at room temperature after cooldown from 300 °C and storing under vacuum for 11 days at room temperature showing the areas selected for quantitative EDS analysis (results in Table S11). Rh and Pt are mapped in red and green, respectively.**

Table S11 **Results from quantitative EDS analysis of Rh(core)-Pt(shell) nanoparticles (NP43-47) at room temperature after cooldown from 300 °C and storing under vacuum for 11 days at room temperature (see Fig. S35). The Pt-Rh molar composition is normalized to 100 at. %.**

Nanoparticle	Time at RT	Rh [at. %]	Pt [at. %]	Nanoparticle size [nm]	Element distribution
NP43	>11 days	57 ±2.9	43 ±2.9	13.1	Segregated
NP44	>11 days	45 ±2.9	55 ±2.9	15.6	Segregated
NP45	>11 days	46 ±3.0	54 ±3.0	11.0	Segregated
NP46	>11 days	49 ±3.0	51 ±3.0	17.8	Segregated
NP47	>11 days	56 ±2.9	44 ±2.9	11.6	Segregated

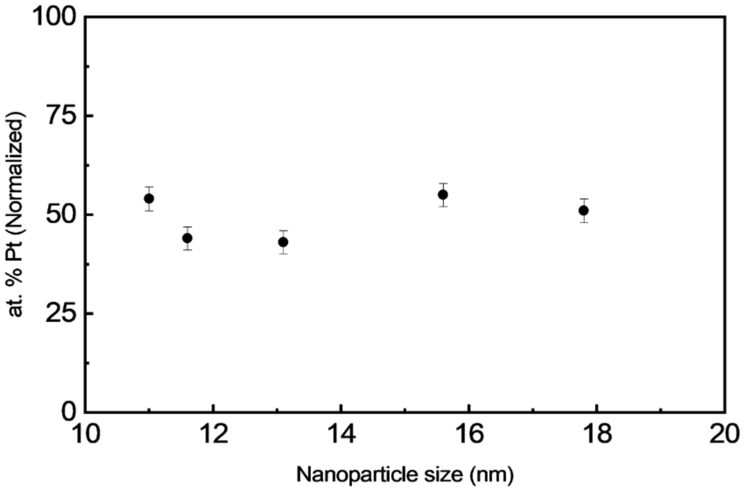

Fig. S36 Normalized Pt content from quantitative EDS results (Table S11) versus Rh(core)-Pt(shell) nanoparticle size (a) and elemental distribution type (b) at room temperature after cooldown from 600 and 300 °C and storing under vacuum for 11 days. The Pt-Rh molar composition is normalized to 100 at. %.

References

1. M. Jensen, B. Gonano, W. Kierulf-Vieira, P. J. Kooyman and A. O. Sjåstad, *RSC Advances*, 2022, **12**, 19717-19725.
